# Anti-nucleocapsid antibody levels and pulmonary comorbid conditions are linked to post–COVID-19 syndrome

**DOI:** 10.1172/jci.insight.156713

**Published:** 2022-07-08

**Authors:** Xiaolin Jia, Shu Cao, Alexandra S. Lee, Monali Manohar, Sayantani B. Sindher, Neera Ahuja, Maja Artandi, Catherine A. Blish, Andra L. Blomkalns, Iris Chang, William J. Collins, Manisha Desai, Hena Naz Din, Evan Do, Andrea Fernandes, Linda N. Geng, Yael Rosenberg-Hasson, Megan Ruth Mahoney, Abigail L. Glascock, Lienna Y. Chan, Sharon Y. Fong, CLIAHUB Consortium, Chan Zuckerberg Biohub, Maira Phelps, Olivia Raeber, Stanford COVID-19 Biobank Study Group, Natasha Purington, Katharina Röltgen, Angela J. Rogers, Theo Snow, Taia T. Wang, Daniel Solis, Laura Vaughan, Michelle Verghese, Holden Maecker, Richard Wittman, Rajan Puri, Amy Kistler, Samuel Yang, Scott D. Boyd, Benjamin A. Pinsky, Sharon Chinthrajah, Kari C. Nadeau

**Affiliations:** 1 Department of Medicine, Stanford University School of Medicine, Stanford, California, USA.; 2 Division of Hospital Medicine,; 3 Sean N. Parker Center for Allergy and Asthma Research,; 4 Division of Primary Care and Population Health Stanford University, Stanford, California, USA.; 5 Chan Zuckerberg Biohub, San Francisco, California, USA.; 6 Division of Infectious Diseases, Stanford University, Stanford, California, USA.; 7 Department of Microbiology and Immunology, Stanford University, Stanford, California, USA.; 8 Department of Emergency Medicine, Stanford University, Stanford, California, USA.; 9 Quantitative Sciences Unit, Stanford University, Stanford, California, USA.; 10 See Supplemental Acknowledgments for consortium details.; 11 Department of Pathology, Stanford University School of Medicine, Stanford, California, USA.; 12 Division of Pulmonary, Allergy, and Critical Care Medicine, Stanford University, Stanford, California, USA.; 13 Institute for Immunity, Transplantation, and Infectious Diseases, Stanford University, Stanford, California, USA.

**Keywords:** Infectious disease, Immunoglobulins

## Abstract

**BACKGROUND:**

Prolonged symptoms after SARS-CoV-2 infection are well documented. However, which factors influence development of long-term symptoms, how symptoms vary across ethnic groups, and whether long-term symptoms correlate with biomarkers are points that remain elusive.

**METHODS:**

Adult SARS-CoV-2 reverse transcription PCR–positive (RT-PCR–positive) patients were recruited at Stanford from March 2020 to February 2021. Study participants were seen for in-person visits at diagnosis and every 1–3 months for up to 1 year after diagnosis; they completed symptom surveys and underwent blood draws and nasal swab collections at each visit.

**RESULTS:**

Our cohort (*n* = 617) ranged from asymptomatic to critical COVID-19 infections. In total, 40% of participants reported at least 1 symptom associated with COVID-19 six months after diagnosis. Median time from diagnosis to first resolution of all symptoms was 44 days; median time from diagnosis to sustained symptom resolution with no recurring symptoms for 1 month or longer was 214 days. Anti-nucleocapsid IgG level in the first week after positive RT-PCR test and history of lung disease were associated with time to sustained symptom resolution. COVID-19 disease severity, ethnicity, age, sex, and remdesivir use did not affect time to sustained symptom resolution.

**CONCLUSION:**

We found that all disease severities had a similar risk of developing post–COVID-19 syndrome in an ethnically diverse population. Comorbid lung disease and lower levels of initial IgG response to SARS-CoV-2 nucleocapsid antigen were associated with longer symptom duration.

**TRIAL REGISTRATION:**

ClinicalTrials.gov, NCT04373148.

**FUNDING:**

NIH UL1TR003142 CTSA grant, NIH U54CA260517 grant, NIEHS R21 ES03304901, Sean N Parker Center for Allergy and Asthma Research at Stanford University, Chan Zuckerberg Biohub, Chan Zuckerberg Initiative, Sunshine Foundation, Crown Foundation, and Parker Foundation.

## Introduction

As of November 2021, SARS-CoV-2 has been responsible for more than 248 million infections and over 5 million deaths worldwide (1). Though there are currently no established definitions, some recent publications, as well as the CDC, define postacute sequelae of SARS-CoV-2 infection (PASC) — or long COVID — as the constellation of symptoms occurring 4 or more weeks after acute COVID-19 infection (2, 3). A recent systematic review showed that an average of 73% of study participants reported at least 1 persistent symptom 60 days after diagnosis; the most common symptoms were fatigue, shortness of breath, and sleep disturbance (4). A metaanalysis of 57 studies showed that more than half of COVID-19 survivors experienced PASC 6 months after recovery (5). These patients can develop a wide range of new sequelae attributable to SARS-CoV-2 infection months after the acute illness, including chronic respiratory failure, cardiac arrhythmias, and gastrointestinal and neuropsychiatric symptoms (6). Even younger adults with no preexisting medical history and milder acute disease are at risk of developing these clinical sequelae (7).

Although there is growing knowledge regarding the long-term symptoms of COVID-19, there is a lack of systematic evaluations on which factors, or serological markers, influence the development and persistence of these symptoms. There is also limited information regarding whether certain commonly used treatments (such as remdesivir) can reduce long-term sequelae of COVID-19. Here, we present data from a long-term study that investigated the continuing symptoms and serologic responses to SARS-CoV-2 to comprehensively characterize a cohort of ethnically diverse COVID-19 patients.

## Results

**Figure 1:**
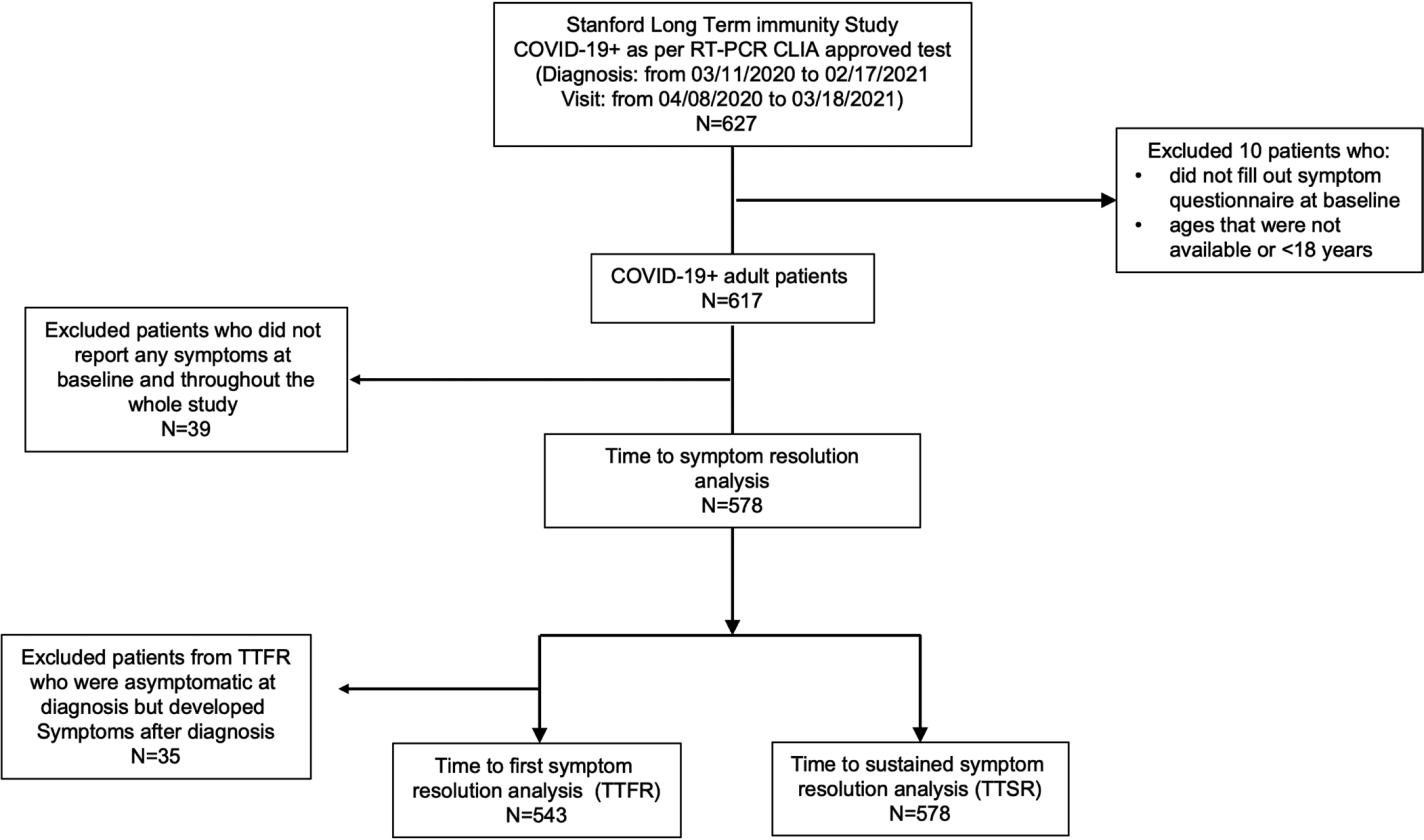
Consort diagram.

A total of 627 participants who had a positive SARS-CoV-2 reverse transcription PCR (RT-PCR) test at Stanford Health Care were recruited from the inpatient and outpatient settings from March 2020 to February 2021 (Figure 1 and Supplemental Table 1; supplemental material available online with this article; https://doi.org/10.1172/jci.insight.156713DS1). A total of 617 SARS-CoV-2^+^ participants were included for analysis, as well as 20 SARS-CoV-2^–^ controls. Participants’ demographic and clinical characteristics are summarized in Table 1. A total of 105 participants were enrolled for 6 months or more after diagnosis (Supplemental Table 2). Sixteen participants died as of March 19, 2021, 9 of whom had critical COVID-19 disease. The demographics for the negative control group are summarized in Table 2.

### Symptoms at diagnosis.

**Figure 2:**
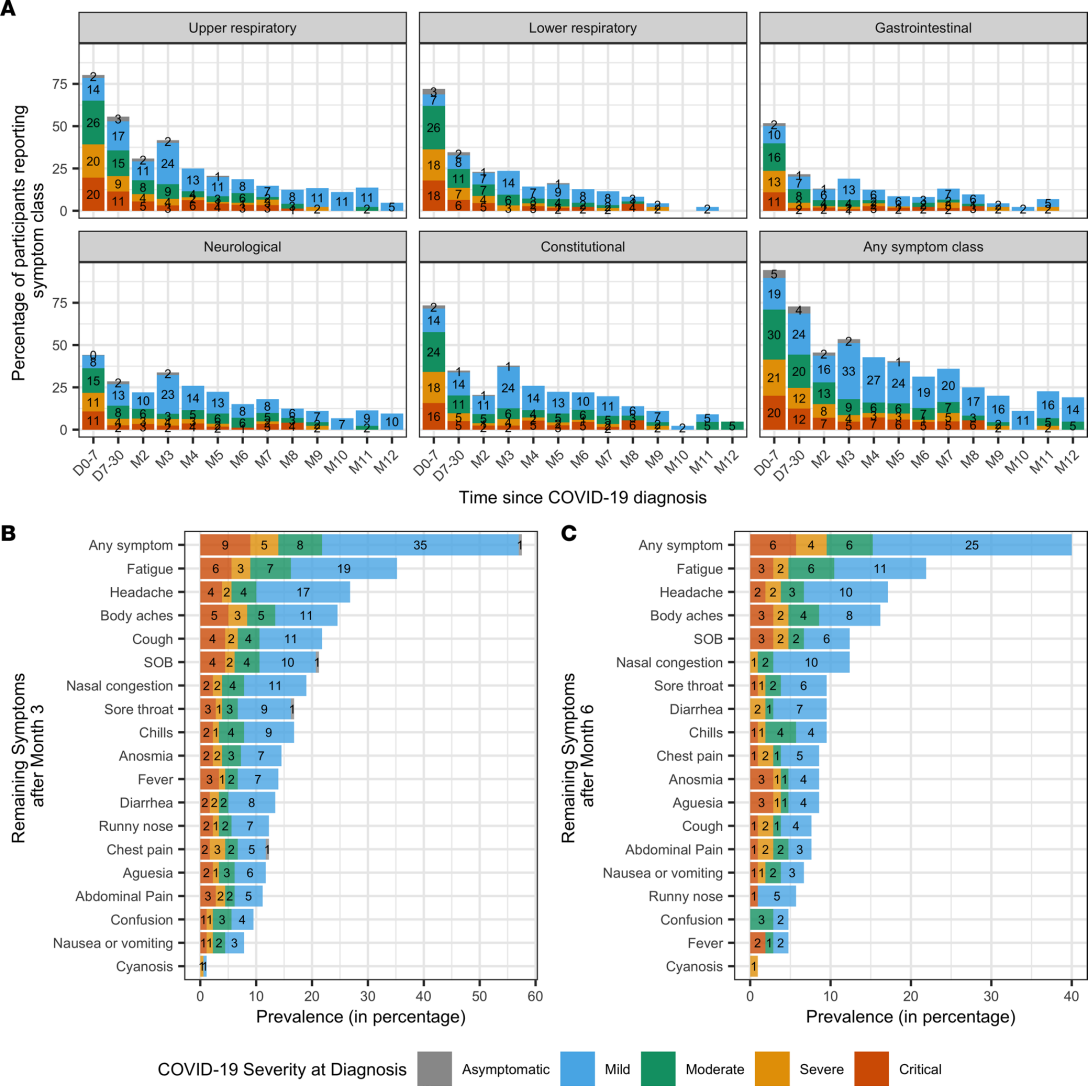
Symptom distribution over time. (**A**) Symptom distribution by symptom class over time since COVID-19 diagnosis, stratified by disease severity. We grouped the symptoms into 5 classes based on the organ systems: 1, upper respiratory symptoms included cough, nasal congestion, runny nose, and sore throat; 2, lower respiratory symptoms included cyanosis, chest pain, and shortness of breath; 3, gastrointestinal symptoms included abdominal pain, diarrhea, nausea, or vomiting; 4, neurological symptoms included ageusia, anosmia, headache, and confusion; and 5, constitutional symptoms included body aches, chills, fatigue, and fever. (**B** and **C**) Prevalence of symptoms persisting > 3 months (*n* = 179) (**B**) and prevalence of symptoms persisting > 6 months (*n* = 105) since COVID-19 diagnosis by disease severity (**C**). D, day; M, month; SOB, shortness of breath.

Hispanic ethnicity, older age, comorbid diabetes, and hypertension were associated with more severe disease at diagnosis (*P* < 0.001, *P* = 0.003, *P* = 0.001, *P* = 0.001, respectively) (Supplemental Figure 1, A–D). There was no significant association between preexisting lung disease and acute COVID-19 disease severity (*P* = 0.29, Supplemental Figure 1E). The most common symptoms reported at diagnosis were cough (74.4%), shortness of breath (65.6%), fever (58.6%), and nausea/vomiting (>50%) (Supplemental Figure 2). Overall, participants had higher rates of respiratory and constitutional symptoms (fever, chills, fatigue, body aches) at diagnosis compared with other symptom categories (Figure 2A and Supplemental Figure 2).

### Long-term symptoms.

After 3 months, 57.5% of 179 participants who filled out questionnaires reported persistent symptoms. Even participants who were asymptomatic at initial diagnosis (*n* = 49) could continue to develop long-term symptoms (Figure 2). The most common symptoms were fatigue (35.2%), followed by headache (26.8%) and body aches (24.6%) (Figure 2B). After 6 months, of 105 participants who filled out questionnaires, 40% reported persistent symptoms, the most common being fatigue (21.9%), followed by headache (17.1%) and body aches (16.2%) (Figure 2C). Notably, 4 of 21 participants reported upper respiratory, neurologic, and constitutional symptoms 12 months after diagnosis. Among 105 participants with symptom data 6 months or longer after diagnosis and where adequate data were available for statistical analysis, there was no significant difference in demographic characteristics, disease severity at diagnosis, or age between those reporting symptoms (*n* = 42) and those who did not (*n* = 63) (Supplemental Table 2 and Supplemental Figure 3).

**Figure 3:**
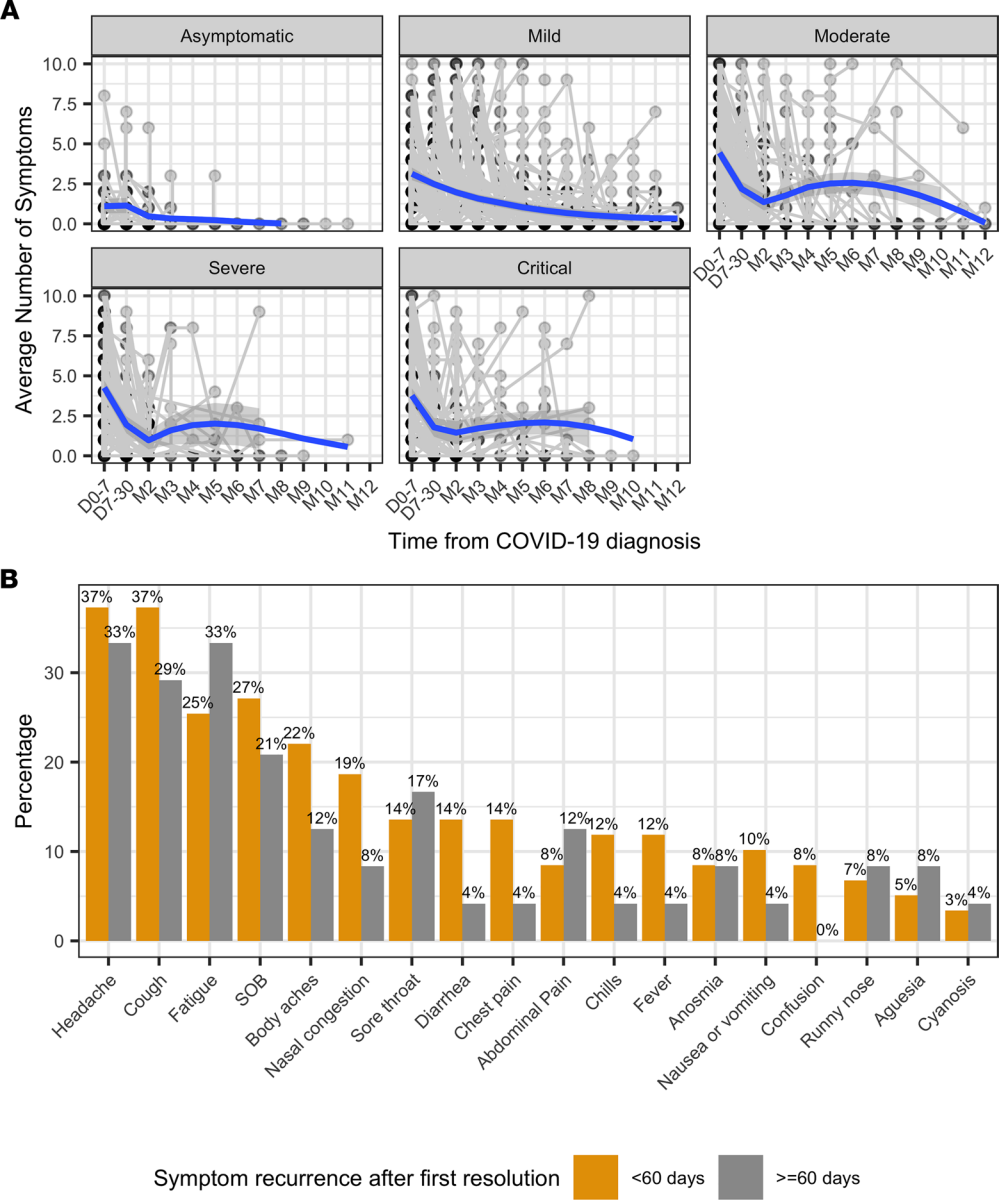
Number of symptoms over time and recurring symptoms. (**A**) Number of symptoms over time stratified by severity at diagnosis. Gray dots indicate the average number of symptoms per person at each time range. Loess curves (shown in blue) present the average number of symptoms over time. (**B**) Numbers and percentages of recurring symptoms stratified by time from first resolution to recurrence within 60 days (*n* = 59) versus ≥ 60 days (*n* = 24). D, day; M, month; SOB, shortness of breath.

The number of symptoms reported within the first week, weeks 2–4, and each month afterward — stratified by severity at diagnosis — is shown in Figure 3A. The number of symptoms diminished significantly over time (*P* < 0.001). Notably, the number of reported symptoms increased after the second month in participants with moderate, severe, or critical disease at diagnosis, indicating recurrence of symptoms after initial resolution. The distribution of symptoms that recurred before and after 2 months since first resolution is shown in Figure 3B. There were 83 participants with symptom recurrence after the first resolution of all symptoms, 59 of whom had recurrence < 60 days after the first resolution. The most frequently reported recurring symptoms were headache, cough, fatigue, and shortness of breath. There was no significant association between the recurrence time and type of symptom.

To evaluate the potential risk factors associated with symptoms > 30 days after diagnosis, we performed a logistic regression model on the proportion of participants reporting any symptom after 30 days, as a function of COVID-19 disease severity, age, ethnicity, lung disease, diabetes, and hypertension. Lung disease and critical COVID-19 disease were identified to be significantly associated with higher risk of reporting symptoms after 30 days (OR [95% CI]: 1.22 [1.07–1.39], *P* = 0.003; OR [95% CI]:1.24 [1.06–1.44], *P* = 0.006, respectively). Additionally, Hispanic ethnicity had lower risk of reporting symptoms after 30 days (OR [95% CI]: 0.88 [0.78–1.00], *P* = 0.022).

### Time to symptom resolution.

Among 617 participants, 578 (93.7%) reported at least 1 symptom. Participants with COVID-19 who were asymptomatic at diagnosis (*n* = 35) were not included in the time to first symptom resolution (TTFR) analysis (Figure 1). The median TTFR (performed on the 543 participants) was 44 days (95% CI, 41–49), and median time to sustained symptom resolution (TTSR) (performed on the 578 participants) was 214 days (95% CI, 178–238).

**Figure 4:**
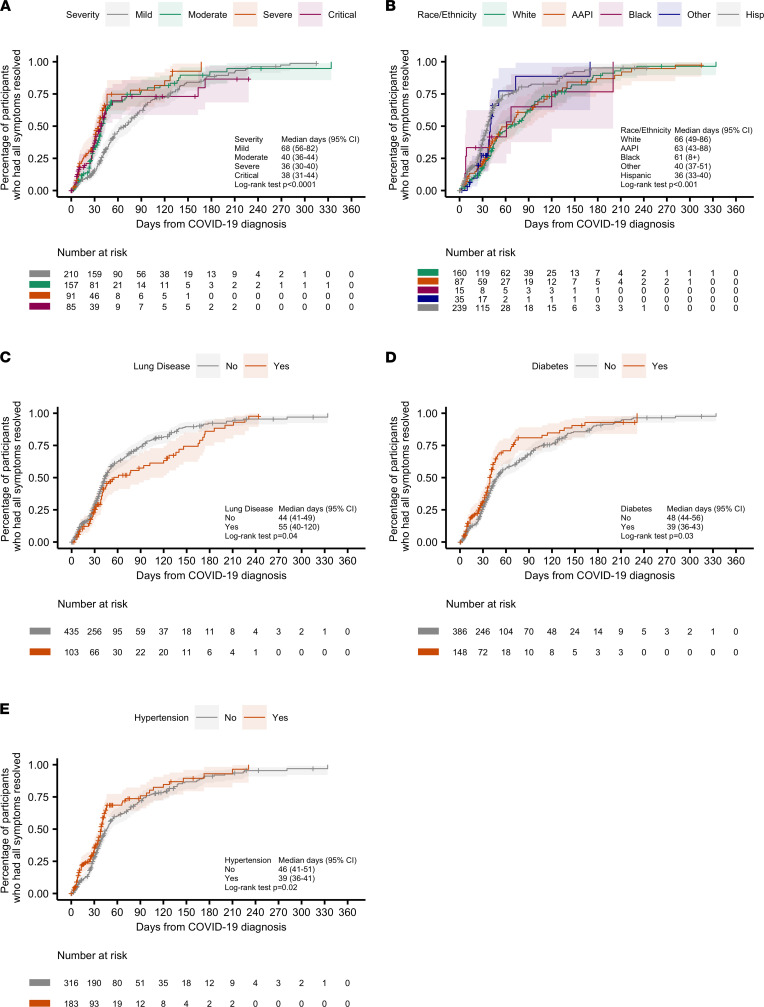
Time to first symptom resolution. (**A**–**E**) Kaplan-Meier curves of time to first symptom resolution and corresponding 95% CI bands by COVID-19 severity at diagnosis (**A**), race and ethnicity (**B**), lung disease (either asthma or chronic obstructive pulmonary disease as a historical condition prior to diagnosis of COVID-19) (**C**), diabetes (historical condition prior to diagnosis of COVID-19) (**D**), and hypertension (historical condition prior to diagnosis of COVID-19) (**E**). Participants who did not reach the endpoint are censored at their last visit (represented by the + symbol).

Kaplan-Meier plots and log-rank tests were used to evaluate the associations between time to symptom resolution and participant characteristics (Figure 4, A–E). Certain ethnic groups had significantly different TTFR (*P* < 0.001, Figure 4B). Additionally, participants who reported lung disease as a comorbid condition (asthma or COPD) had a longer TTFR (55 versus 44 days; *P* = 0.04, Figure 4C). There was no significant association between TTFR and age group (51 and 44 versus 41 days for ages 18–40 and 41–64 versus age 65+ groups; *P* = 0.20), or remdesivir treatment (38 versus 40 days for who received versus those who did not receive remdesivir treatment; *P* = 0.18).

The differences in TTSR were not statistically significant across severity groups or ethnic groups. Patients with prior lung disease experienced longer TTSR (188 versus 200 days; *P* = 0.009), while patients with hypertension or diabetes did not. There was no significant association between TTSR and age (200 and 216 versus 214 days for ages 18–40 and 41–64 versus age 65+ groups; *P* = 0.50), or remdesivir treatment (198 versus 197 days for those who received versus those who did not receive remdesivir treatment; *P* = 0.2). We further analyzed the potential correlations between lung disease and other demographic characteristics and found that significantly less lung disease was reported among Hispanic participants compared with other race and ethnicity group (14% versus 23%; *P* = 0.007, Supplemental Figure 4), while there were no significant interactions between lung disease and ethnicity when predicting the TTFR or TTSR. The incidence of lung disease was not associated with age group (proportion of participants who had lung disease for 18–40, 41–64, 65+ years was 14%, 18%, and 24%, respectively; *P* = 0.087).

### Associations between symptoms.

**Figure 5:**
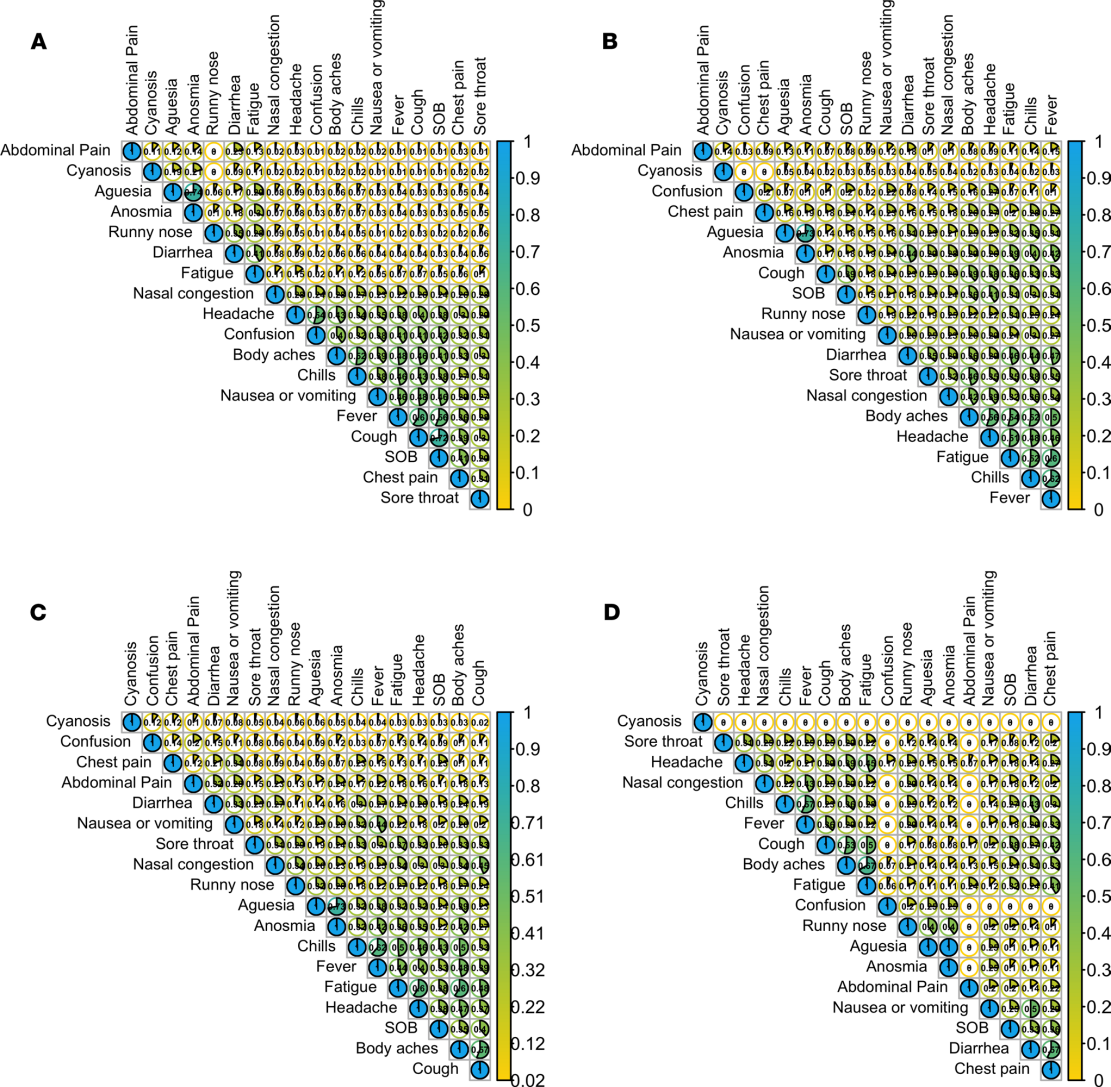
Cooccurrence of symptoms. (**A**–**D**) Cooccurrence of symptoms at month 1 (**A**), month 2 (**B**), month 3 (**C**), and month 6 (**D**) since COVID-19 diagnosis. The value and color filled pie in each cell represents the Jaccard similarity index that was used to determine the prevalence of cooccurrence of symptoms among the entire cohort, accounting for the different number of participants presenting each symptom. The index is defined as the number of participants who are positive for both symptoms divided by the number of people who are positive for either symptom. Higher values with larger pie sizes indicate more similarity between the 2 symptoms.

The most frequently reported cooccurring symptom at month 1 was ageusia (loss of taste) and anosmia (loss of smell) with a Jaccard similarity index — defined as the number of participants who are positive for both symptoms divided by the number of people who are positive for either symptom — of 0.74, followed by cough and shortness of breath (0.72) (Figure 5A). At month 2–3, there was high cooccurrence between ageusia and anosmia, and between chills, fever, fatigue, headache, shortness of breath, body aches, and cough (Figure 5, B and C). The Jaccard similarity index between anosmia and ageusia remained high even 6 months after diagnosis (Figure 5D).

**Figure 6:**
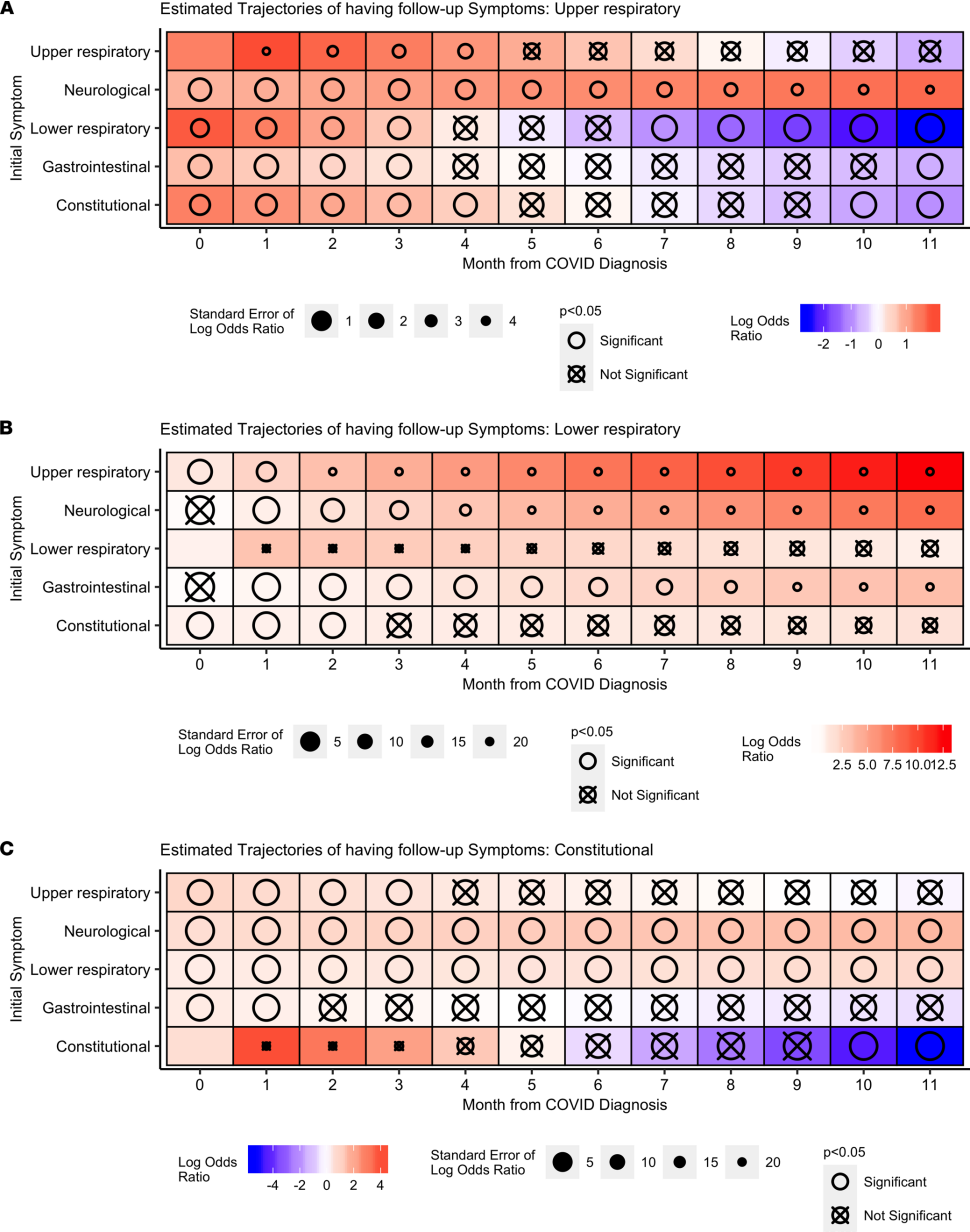
Associations between symptoms at diagnosis and at follow-up months. Association between the presence of each symptom class at diagnosis and follow-up months from COVID diagnosis. (**A**–**C**) upper respiratory symptoms, lower respiratory symptoms, and constitutional symptoms.

The presence of neurological symptoms (ageusia, anosmia, headache, and/or confusion) at diagnosis was strongly associated with upper respiratory symptoms up to 11 months after diagnosis (Figure 6A). Upper respiratory, neurological, and gastrointestinal symptoms at diagnosis were strongly associated with lower respiratory symptoms at follow-up (Figure 6B). The presence of neurological and lower respiratory symptoms at diagnosis was associated with constitutional symptoms at follow-up (Figure 6C).

### Antigen and antibody to SARS-CoV2.

**Figure 7:**
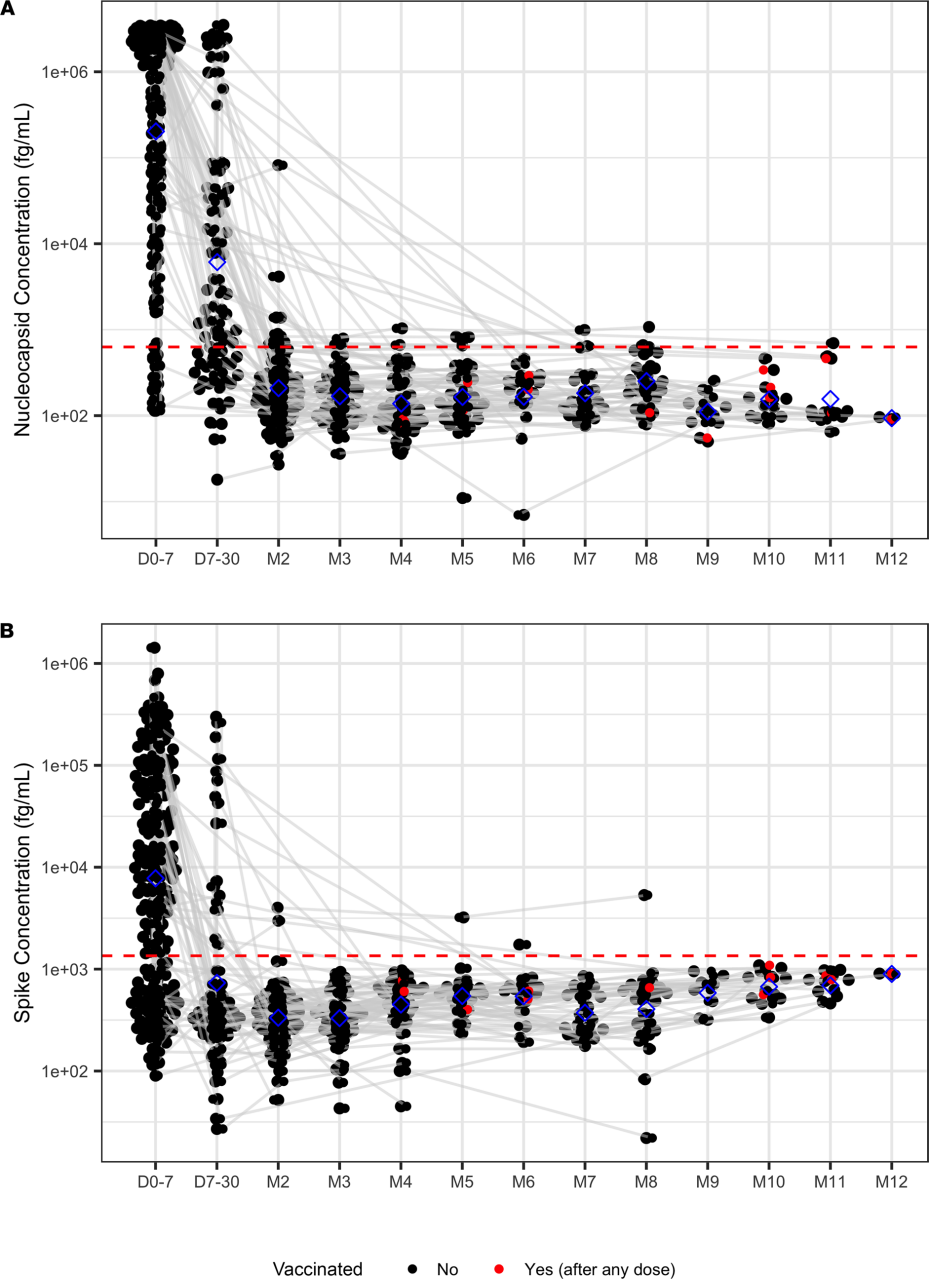
Nucleocapsid and spike antigen protein concentrations. (**A**) Nucleocapsid protein concentrations over time. (**B**) Spike protein concentrations over time. Red dashed horizontal lines indicate the thresholds for positive or negative that are defined as the mean ± 3 SD signal from *n* = 80 prepandemic (obtained before October 2019) healthy control samples. Blue diamonds indicate the means of concentration. Day 0 is the day of diagnosis. Gray lines connect the same participants. D, day; M, month.

The plasma antigen data for SARS-CoV-2 nucleocapsid (N) and spike (S) proteins were available for 304 participants who were positive for COVID-19, who were similar in demographic profiles as the entire cohort (Supplemental Table 3), and for 20 negative controls. SARS-CoV-2 antigenemia is highly sensitive for detection of COVID-19 infection, and levels of antigenemia, similar to RNAemia, have been shown to correlate with disease severity (8). N and S antigen concentrations significantly decreased over time (coefficient [95% CI]: N: –0.67 [–0.76 to –0.59], *P* < 0.001; S: –0.23 [–0.29 to –0.17], *P* < 0.001, Figure 7) in COVID-19^+^ participants, and participants with more severe disease at baseline had higher N and S antigen concentrations (coefficient [95% CI]: N: 0.66 [0.44–0.89], *P* < 0.001; S: 0.29 [0.15–0.43], *P* < 0.001). Cox regression showed no significant associations between antigen values at the first week of diagnosis with TTFR (HR [95% CI]: N: 0.96 [0.89–1.03], *P* = 0.23; S: 0.99 [0.90–1.10], *P* = 0.92) or TTSR (HR [95% CI]: N: 1.0[0.82–1.30], *P* = 0.78; S: 1.03 [0.79–1.35], *P* = 0.83). The sensitivity analysis identified the optimal cut-points for N antigen reported symptoms level in TTFR. Higher antigen N level at the first week of diagnosis showed association with longer TTFR (cutoff: 2153 fg/mL; hazard ratio [HR] [95% CI]: 0.41 [0.22–0.79]; log-rank test, *P* = 0.005) but not TTSR; S antigen levels in the first week of diagnosis were not significantly associated with TTFR or TTSR (Supplemental Figure 5). There were 9 participants with detectable plasma N antigen 60 days after diagnosis, though levels were low — a mean of 146 fg/mL above the threshold for positivity (630 fg/mL). One of these individuals also had detectable plasma S antigen above the threshold for positivity (1352 fg/mL) even 8 months after the initial COVID-19 diagnosis. In addition, 1 participant was negative for plasma N antigen but had detectable plasma S antigen 60 days after diagnosis. Five of these 10 participants reported symptoms 3 months after initial COVID-19 diagnosis. Their median TTFR and TTSR were 77 and 237 days, respectively; this was longer than that of the cohort, though not statistically significant. The control group had no SARS-CoV-2 plasma antigens detected.

**Figure 8:**
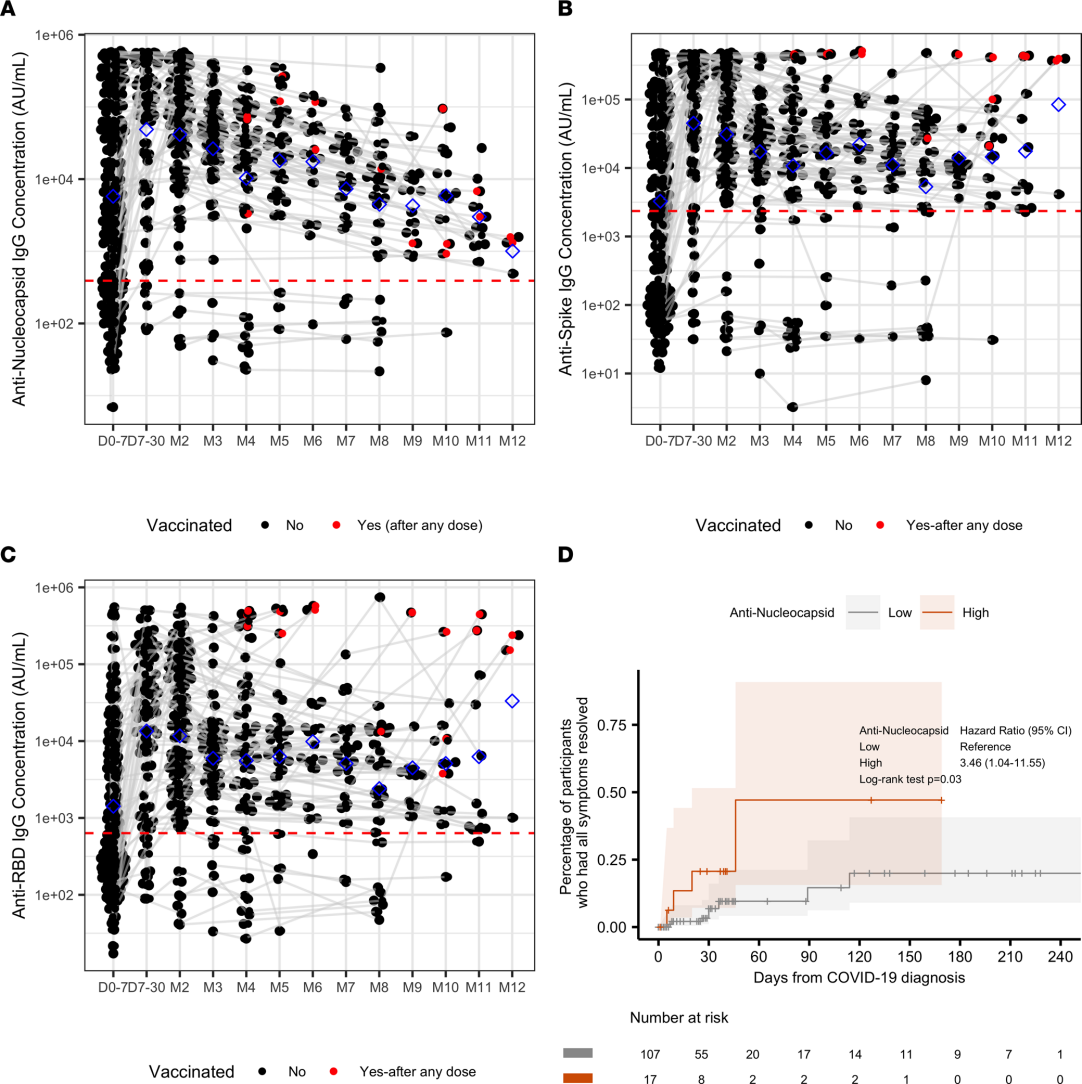
Anti-N, anti-S, and anti-RBD IgG concentrations. (**A**) Anti-N IgG concentrations over time. (**B**) Anti-S IgG concentrations over time. (**C**) Anti-RBD IgG concentrations over time. Red dashed horizontal lines indicate the thresholds for positive or negative that are defined as the mean ± 3 SD signal from *n* = 37 prepandemic (obtained prior to October 2019) samples. Blue diamonds indicate the means of concentration. Day 0 is the day of diagnosis. Gray lines connect the same participants. D, day; M, month. (**D**) Kaplan-Meier curves of time to sustained symptom resolution and corresponding 95% CI bands by anti-N IgG within 7 days after COVID-19 diagnosis. Log-rank test *P* value is shown. Low versus high groups were determined by the optimal point using the maximally selected rank statistics method. Low ≤ 149,452.067 AU/mL; High > 149,452.067 AU/mL.

Anti-N, anti-S, and anti-RBD IgG data were available for the same 304 participants COVID-19^+^ participants and 20 controls at the same time points. The threshold for positive IgG level was defined as above the mean ± 3 SD from 37 prepandemic samples (red line; Figure 8, A–C). In general, IgG concentrations increased in the first month (*P* < 0.001 for anti-N, anti-S, and anti-RBD) and then significantly decreased afterwards (anti-N: *P* < 0.001; anti-S: *P* = 0.037; anti-RBD: *P* = 0.051) in COVID-19^+^ participants (Figure 8, A–C). More severe disease at diagnosis was associated with higher antibody levels (*P* < 0.001 for anti-N, anti-S, and anti-RBD) during the entire study follow-up period. Antibody levels remained positive for most participants 6 months (92% of 59 participants) and 9 months (96% of 26 participants) after diagnosis. The antibody values at the first week of diagnosis were not associated with TTFR or TTSR. The sensitivity analysis identified the optimal cut-points for anti-N IgG level in TTSR. Higher anti-N IgG concentrations in the first week were associated with shorter TTSR (cutoff: 149452.067 AU/mL; HR [95% CI]: 3.46 [1.04–11.55]; log-rank test, *P* = 0.03, Figure 8D). We further determined whether anti-N IgG was associated with odds of reporting symptoms > 30 days after diagnosis. Though there was a trend toward lower probability of reporting symptoms with higher anti-N IgG level, this did not reach statistical significance (36% versus 55%, *P* = 0.43). Anti-S IgG and anti-RBD IgG levels were not significantly associated with TTFR or TTSR (Supplemental Figure 6). Higher N and S antigen concentrations were significantly correlated with lower anti-N and anti-S IgG concentrations, respectively (Spearman’s correlation coefficient: N antigen versus anti-N: –0.43 with *P* < 0.001; S antigen versus anti-S: –0.29 with *P* < 0.001) (Supplemental Figure 7). Additionally, higher anti-S IgG concentration was associated with higher anti-N IgG (*P* < 0.001, Supplemental Figure 8). The control group had no SARS-CoV-2–specific antibodies detected.

### Associations with coinfections.

Impact of potential upper respiratory coinfections was evaluated through metatranscriptomics Next Generation Sequencing (mNGS) analysis of libraries generated using participants’ nasal swab-derived RNA.

In our cohort, 141 of 617 COVID-19^+^ participants, and 7 of 20 COVID-19^–^ participants consented to longitudinal nasal swab specimen collections after their initial diagnostic nasal swabs. A total of 309 subsequent longitudinal nasal swab specimens were collected and underwent RNA extraction for COVID-19 RT-PCR assay to determine if SARS-CoV-2 RNA remained detectable or appeared over the course of follow-up and to determine how such detection related to symptom severity and long COVID outcomes. SARS-CoV-2 was detected in 12 of the COVID-19^+^ individuals in the 2–7 months after their initial COVID-19 diagnosis by RT-PCR (Supplemental Table 4). Among these postdiagnosis COVID-19^+^ patients, the level of SARS-CoV-2 was generally low (C_t_ range = 31.58–37.3) and was typically detected in only 1 of the longitudinal follow-up nasal swab samples collected from a participant. However, SARS-CoV-2 was detected in a single participant more than once: at 2 months and at 5 months after initial COVID-19 diagnosis, but not in their intervening nasal swabs sampled at 3 and 4 months, or their nasal swabs sampled at 6 and 7 months. SARS-CoV-2 amplicon sequencing, mNGS, or combined analysis of both these assays were performed to investigate which SARS-CoV-2 variants were detected and if the participant with 2 positive samples harbored the same or distinct variants; however, the low yield of SARS-CoV-2 genome coverage acquired from all these samples was insufficient to elucidate these questions.

After COVID-19 RT-PCR, 203 of the 309 nasal swab samples had sufficient remaining RNA to yield RNA-Seq libraries of sufficient quality for mNGS analysis (see Bioinformatics pipeline section in Supplemental Methods and Supplemental Table 5). Among these, statistically significant genus-level detection of microbes was feasible in 137 samples, including 7 of the 13 COVID-19^+^ samples. The sparseness of this sampling limited our ability to investigate the association of additional viral, bacterial, or eukaryotic pathogens and long COVID-19 symptoms. Despite this, several notable findings emerged from the mNGS analysis. First, viral pathogens were detectable, albeit in a small fraction of the mNGS samples (Supplemental Table 6). Statistically significant numbers of reads derived from human rhinoviruses (HRV) were detected in 3 of 203 mNGS samples. Sufficient numbers and diversity of HRV reads were present in each of these samples to infer species level information, indicating that 3 distinct HRV species were detected (HRVA 28, HRVB 91, and HRVC 56). These HRV^+^ samples mapped to 3 different study participants, all of whom were diagnosed as COVID-19^+^ at baseline, and exhibited a range of COVID-19 symptom severity: mild for 1 participant (HRVB 91 detection), critical condition for another (HRVC 56 detection), and moderate symptoms for the third participant (HRVA 28 detection). Of these 3, the participant with HRVA 28 detection reported symptoms beyond 6 months from initial diagnosis. Interestingly, statistically significant numbers of SARS-CoV-2–derived reads were detected in 1 of the 7 COVID-19^+^ participants’ follow-up visit swab, and this participant also tested positive for SARS-CoV-2 mRNA by RT-PCR. This participant, too, reported symptoms beyond 6 months from initial diagnosis.

In contrast to the relatively rare detection of human viral pathogens in the mNGS samples, detection of bacterial sequences was fairly common, with 137 of the 203 samples found to harbor at least 1 high-confidence bacterial genera call. A diverse set of bacterial genera were identified, including *Corynebacterium*, *Moraxella*, *Streptococcus*, *Staphylococcus*, *Klebsiella*, *Haemophilus*, and *Pseudomonas*(Supplemental Figure 9 and Supplemental Table 7). Here, the sparesness of data limited our ability to identify beyond the genus level. These bacterial genera were detected across mNGS samples derived from both COVID-19^+^ and COVID-19^–^ participants. Moreover, bacterial genera were also detected in some of the samples where viral agents had been identified. For instance, 4 of 7 COVID-19^+^ nasal swabs that were included in the mNGS analysis harbored statistically significant numbers of reads derived from diverse bacterial genera. Likewise, 1 of 3 HRV^+^ samples (the HRVA 28^+^ sample) had a statistically significant number of reads derived from a single bacterial genera (*Pseudomonas*). However, the role of these bacterial genera as pathogens or commensals in the upper respiratory tract compartment remains unclear. As such, it was not possible to infer associations for bacteria or virus or bacterial/viral coinfections with long COVID symptoms.

In addition, since EBV and CMV infection has been linked with PASC, (9, 10) we estimated anti-CMV and anti-EBV antibodies in participants’ plasma samples by Luminex assay (Supplemental Methods). In total, 260 plasma samples from 186 COVID-19^+^ participants from baseline to 8 months after COVID-19 diagnosis were tested. Raw MFI readouts for IgG and IgM were grouped into high versus low based on empirically determined cut-offs. A high (>1000) anti-CMV IgG–associated MFI was interpreted to suggest CMV preexposure, whereas > 200 anti-EBV IgM–associated MFI was interpreted to imply a recent EBV infection. Among 186 COVID-19^+^ participants, 30 had a higher anti-EBV IgM readout. Of these 30, 13 and 5 participants reported sustained symptoms after 3 months and 6 months respectively after COVID diagnosis. Higher anti-CMV IgG or anti-EBV IgM readout for a given participant was not associated with longer TTFR (anti-CMV IgG: *P* = 0.18; anti-EBV IgM: *P* = 0.15) or TTSR (anti-CMV IgG: *P* = 0.17; anti-EBV IgM: *P* = 0.36).

## Discussion

In this prospective cohort study, we characterized the manifestations, trajectory, and variability of COVID-19 in a diverse group of participants, ranging from asymptomatic outpatients to those hospitalized for critical disease. An advantage of our study was the collection of longitudinal symptom data in combination with serologic data for antigen and antibody levels up to 12 months after diagnosis. We also provide longitudinal symptom data on a demographically diverse population, a heretofore understudied aspect of the pandemic.

Consistent with prior reports, Hispanic participants made up a larger percentage of severe and critical cases in our cohort (11). In total, 57.5% of participants reported persistent symptoms after 3 months, and 40% reported persistent symptoms after 6 months. The most common persistent symptoms were fatigue, headache, body aches, and nasal congestion. These findings are consistent with previous studies (4–6, 12–16). Patients with mild COVID-19 infections who were never hospitalized can develop prolonged symptoms (16–18). We confirmed that all disease severities had a similar risk of long-term symptoms after COVID, and importantly, participants with no symptoms or mild disease at initial diagnosis were still at risk for long-term symptoms with COVID-19.

We used the terminology of TTFR and TTSR to distinguish between the first resolution of COVID-19–related symptoms and the resolution of these symptoms without recurrence for at least 1 month. This is important, as we noticed a significant number of participants, especially those with moderate to severe disease, had recurrence of symptoms after initial symptom resolution. This aspect of long-term symptoms with COVID-19 has not been examined in previous studies, and we are one of the first studies to our knowledge to look at what symptoms recurred most frequently up to a year after diagnosis. Headache, cough, fatigue, and shortness of breath were the most frequently recurring symptoms in our cohort, usually within 60 days of initial resolution. These are also some of the most commonly reported long-term symptoms, so the recurrent nature of these symptoms would be important and can provide practical information for both clinicians and patients.

While we did not establish a long-term control group without COVID-19 infection for comparison, literature on long-term effects after recovery from other viral infections, such as influenza and SARS-CoV-1, suggests that some of the persistent symptoms associated with SARS-CoV-2 are similar (19–21). For instance, a SARS follow-up study found that more than 40.3% of patients experienced chronic fatigue and 27.1% met the CDC’s definition for chronic fatigue syndrome 4 years after hospital discharge (21).

Though there have been studies describing long-term symptoms with COVID-19, most are for shorter durations and only included either severe/hospitalized patients or mild/moderate disease patients. Only a few studies have examined factors that may predict the development of long-term symptoms. Previous studies showed that older age, higher BMI, female sex, and higher number of symptoms at disease onset were associated with greater risk of long-term symptoms (15–17, 22). A study by Augustin et al. reported that long-term symptoms were observed in nonhospitalized COVID-19 patients 7 months after infection. Presence of anosmia and diarrhea during acute infection were associated with higher risk of developing long-term symptoms (18). Another recent study showed that diabetes, SARS-CoV-2 RNAemia, EBV viremia, and presence of autoantibodies at diagnosis may predict long COVID symptoms at 2–3 months after diagnosis (10). Our study looked at some of these clinical and laboratory variables, but not all, and confirmed some of these findings. However, we report here additional features that could predict long COVID. We found that higher symptom frequency early in diagnosis was associated with long-term symptoms out to 1 year. In addition, we report on a more ethnically diverse population than most other studies. Moreover, one of the most unique aspects of our study was that we followed highly dimensional features in our cohort to predict long COVID for over a year. In summary, we were able to identify new factors that could affect time to resolution of symptoms, specifically anti-N antibodies and prior lung disease. Critical disease at diagnosis and non-Hispanic ethnicity were also associated with higher risk of having symptoms > 30 days after diagnosis; however, these factors were not significant when evaluating time to sustained resolution of symptoms. Hispanic ethnicity’s association with lower risk of symptoms > 30 days after diagnosis is likely due to significantly less lung disease reported among Hispanic participants compared with other ethnicity groups. More studies are needed to further understand our findings in the context of global multisite cohorts.

Underlying lung disease (either asthma or COPD) was associated with both longer TTFR and TTSR. Our previous study in asthma patients alone did not include those with COPD or long-term symptoms with COVID-19 (23). Long-term respiratory complications of COVID-19 infection are well documented and include abnormal lung imaging, lung diffusion impairment, and pulmonary fibrosis (24, 25). These additional insults on the lung may exacerbate the underlying lung disease and result in more protracted respiratory symptoms.

We found that COVID-19^+^ participants who had more severe disease had higher plasma N and S antigen concentrations through the duration of the study (8, 26, 27). There was a trend to longer TTFR and TTSR in the group of participants with prolonged antigen positivity after 2 months, though this was not statistically significant, possibly due to the small sample size. Some viral coinfections, such as EBV, have persistent viral or antigen positivity leading to chronic diseases (11, 28, 29), although higher EBV IgM or CMV IgG readout by luminex or mNGS-based microbial signature identification performed in a subset of participants among our cohort were not found to be associated with long-term symptoms. One proposed mechanism for long-term symptoms after COVID-19 is prolonged inflammation from persistent low-level infection (28). Prolonged SARS-CoV-2 viral shedding and persistence of viral RNA in the small bowel has been observed and may be linked to prolonged symptoms (29, 30). The recent report by Su et al. suggests that immune dysregulation is the main driver behind PASC (10). More studies are needed to understand the underlying pathophysiology of PASC.

We found that higher anti-N antibody levels at diagnosis were significantly associated with faster symptom resolution for COVID-19 patients. This is a potentially novel finding, since this test could potentially be applied to clinical settings and may be correlated with post–COVID-19 symptom duration. Furthermore, N protein of SARS-CoV-2 is an important B cell immunogen and can also elicit broad-based cellular immune responses. This protein is believed to be more conserved than other proteins of the virus, such as S and membrane glycoprotein. Results indicate that the N protein may be of potential value in vaccine development for specific prophylaxis and treatment against SARS-CoV-2 (31, 32). These data were obtained from a subgroup with similar demographic features compared with the overall study cohort (Supplemental Table 3). Our findings showed that anti-N IgG levels may possibly be an independent predictor of COVID-19 symptom duration and may be considered for use clinically to predict the risk of long-term symptoms after COVID-19 infection.

Though IgG concentrations significantly decreased after the first month, more than 90% of participants had positive IgG 9 months after diagnosis, and there were no recurrent COVID-19 infections in our cohort. These results confirm previous findings that antibodies confer some protection against COVID-19 infection for a prolonged period after initial illness (33, 34). Though previous studies raised concerns that levels of SARS-CoV-2 antibodies decrease rapidly in the first few months after infection, several recent studies report that antibodies persist for at least 8–11 months after infection, albeit at decreased levels (35–37).

Our study has some limitations. First, our study used questionnaires, which can have potential bias and inaccuracies, given reliance on participant recall. To address this, we verified participant information during in-person visits, and real-time documentation of symptoms occurred at that visit. Secondly, it may be difficult to fully distinguish between symptoms that are related to COVID-19 versus those related to a different etiology. Here, mNGS analysis of RNA isolated from nasal swabs identified a small fraction of samples with evidence of additional viral infections (HRV, SARS-CoV-2) and a high prevalence of bacterial genera present in more than half of the samples. Due to a combination of sparse data available from the mNGS analysis (low number of sequenced samples and low yield of reads for those samples that were sequenced), the limited serologic determination of other infections, and the lack of clarity on the role of bacteria genera in the upper respiratory tract (pathogen or commensal) using research-based assays, these data do not rigorously support — but also do not rule out — a role for coinfection in long COVID. Thirdly, participant follow-up decreased over time after 1 month in the study. To address this, we used similar statistical approaches to account for attrition during long-term follow up as those used by other studies examining long-term symptoms of viral illnesses (19–21, 38, 39). Interestingly, we found that patients with mild disease at baseline had differences in their ability to resolve symptoms of COVID-19. This could be due to ascertainment bias, since we found mild patients more frequently visited our clinical research unit compared with other disease severities. To address any potential bias in which symptomatic participants were more likely to return for visits, we actively encouraged follow-up visits in all participants, including those with no long-term symptoms. On that point, our cohort had similar numbers of participants with and without long-term symptoms of COVID-19. Additional limitations of our study include lack of routine laboratory data for participants, such as blood counts and inflammatory markers, and lack of cellular immunity investigation.

### Conclusion.

In our cohort, we found that 40% of patients reported symptoms at 6 months after diagnosis, and 4 of 21 surveyed still reported persistent symptoms up to 1 year after diagnosis. Patients with severe disease had no significant difference in duration of symptoms compared with those with milder disease. Comorbid lung disease was associated with more prolonged symptoms after infection. Remdesivir use had no effect on time to symptom resolution. Antibodies to SARS-CoV-2 persisted for up to 9 months after diagnosis. Higher anti-N IgG in the first week of SARS-CoV-2 RT-PCR positivity was associated with shorter time to sustained symptom resolution.

Larger studies with longer follow-up periods are needed to further characterize the factors that influence the development of long COVID.

## Methods

### Study design and participants.

Our study is a prospective longitudinal cohort study conducted at Stanford Health Care. We recruited adult (≥18 years old) inpatients and outpatients with known positive RT-PCR SARS-CoV-2 tests performed in a Clinical Laboratory Improvement Amendments–accredited clinical laboratory from March 11, 2020, to February 17, 2021. Outpatient participants were recruited by phone; inpatient participants were recruited in-person in the hospital as close to admission as possible. Exclusion criteria included age < 18 or pregnancy at the time of enrollment. We did enroll patients who were immunocompromised, critically ill, or intubated.

The median time from RT-PCR test to the last follow-up visit was 40 days (IQR: 29–124 days). The clinical case severities of COVID-19 at diagnosis are defined as asymptomatic, mild, moderate, severe, and critical according to the guidelines released by the NIH (40). Study participants were seen for in-person visits at diagnosis and every 1–3 months after diagnosis. Participants were asked to fill out surveys at diagnosis; day 3, 5, 7, and 30; and every 1–3 months; responses were entered directly into a secure RedCap database (https://redcap.stanford.edu/). During study visits, patients completed surveys, nasal swabs, and blood draws. Surveys consisted of questionnaires on current health conditions, COVID-19 symptoms, and testing history. Those who did not fill out the symptom questionnaire at baseline (*n* = 5) were excluded from our analysis (Figure 1 and Supplemental Table 2). Blood samples were tested for presence of SARS-CoV-2 antigens including the N and S protein, as well as antibodies specific to the N and S proteins (see Supplemental Methods). Reported clinical sensitivity and specificity of the MSD IgG SARS-CoV-2 S antigen assay are 97.9% and 97.4%, respectively, at 21 days after positive RT-PCR test in infected patients (41, 42). We also included a control group of 20 participants who were negative for RT-PCR SARS-CoV-2 tests but positive for CLIA-approved influenza testing from September 15 to September 30, 2020. The control group was tested for presence of SARS-CoV-2–specific antigens and antibodies.

There is currently no clear definition for PASC, and we preferred not to define this until global consensus guidelines are written. Instead, we followed long-term symptoms in our cohort, with the main endpoint of this study defined as time to symptom resolution after acute COVID-19 illness, which included the following definitions: (a) TTFR is defined as time from COVID-19 diagnosis to the first visit where no symptoms were documented; (b) TTSR is defined as time from diagnosis to the visit where participants reported resolution of all symptoms and no recurring symptoms for the remainder of the study period (i.e., 12 months). The definitions of TTFR and TTSR were uniform across participants (Figure 1). Participants who did not reach the endpoint were censored at the last visit. Participants who had no symptom questionnaire filled or had no symptoms reported during the study were excluded from TTFR and TTSR analysis, as noted in Figure 1.

### Statistics.

Descriptive statistics were used for participant demographics and clinical characteristics, including median, range, and IQR for continuous variables and for counts and percentages for categorical variables. The distributions of symptoms and symptom classes over time were illustrated using bar graphs. The cooccurrence between symptoms at months 1, 2, 3, and 6 after diagnosis were estimated by Jaccard similarity index. Generalized linear mixed-effects model (GLMM) for the negative binomial family was fitted to estimate the average number of symptoms change over time. Kaplan-Meier curves and log-rank test were used to assess the association between the time to symptom resolution with demographic and clinic characteristics. For each antigen and for antibody data, the GLMM was fitted to estimate its concentration change over time. Other association analyses included χ^2^ test, Kruskal-Wallis test, and Spearman’s correlation test, when appropriate. Logistic regression and odds ratios were used to explore potential risk factors for reporting symptoms after 30 days. Complete case was used to handle missing data that only includes participants for which we have no missing data on the variables of interest. Tests were 2 sided and conducted at the 0.05 level of significance. All analyses were conducted using R software v4.0.3. For further detailed statistical analysis methods, see Supplemental Methods.

Analysis sets are defined as per Figure 1 and further detailed below*.* TTFR: COVID-19^+^ adult patients who were diagnosed as symptomatic at diagnosis and reported at least 1 symptom throughout the whole study, *n* = 543. TTSR: COVID-19^+^ adult who reported at least 1 symptom throughout the whole study, *n* = 578. Cross-sectional analysis at 1, 2, 3, 6 months: COVID-19^+^ adult participants who filled symptom questionnaire at month 1 (<30 days, *n* = 488), month 2 (30-59 days, *n* = 366), month 3 (60-89 days, *n* = 127), and month 6 (150-179 days, *n* = 86), respectively. Cross-sectional analysis after 3 months: COVID-19^+^ adult participants who filled symptom questionnaire after 3 months (≥90 days), *n* = 179. Cross-sectional analysis after 6 months: COVID-19^+^ adult participants who filled symptom questionnaire after 6 months (≥180 days), *n* = 105.

### Study approval.

All protocols used for subject recruitment, enrollment, blood collection, sample processing, and immunological assays with human samples were approved by the Stanford Administrative Panel on Human Subjects in Medical Research. All participants voluntarily enrolled in the study by signing an informed consent form after receiving detailed information of the clinical study.

## Author contributions

KCN, S. Chinthrajah, SY, MM, AJR, and CAB designed the study; ASL, HND, TS, and XJ ran study visits and helped with data collection; KR, BAP, and SDB performed antigen and antibody analysis; S. Cao, MD, and NP performed data and statistical analysis for the paper; XJ and S. Chinthrajah shared in manuscript writing, with input and feedback from all other authors. All other authors conducted experiments on the human samples. SBS, NA, MA, ALB, IC, WJC, ED, AF, LNG, YRH, MRM, AG, LYC, SYF, CZB, MP, OR, TTW, DS, LV, MV, HM, RW, and RP assisted in the writing and editing of the paper. Consortiums helped obtain and store samples. Order of co–first authors was determined based on proportion of contribution.

## Supplementary Material

Supplemental data

ICMJE disclosure forms

Supplemental table 4

Supplemental table 5

Supplemental table 7

## Figures and Tables

**Table 1:**
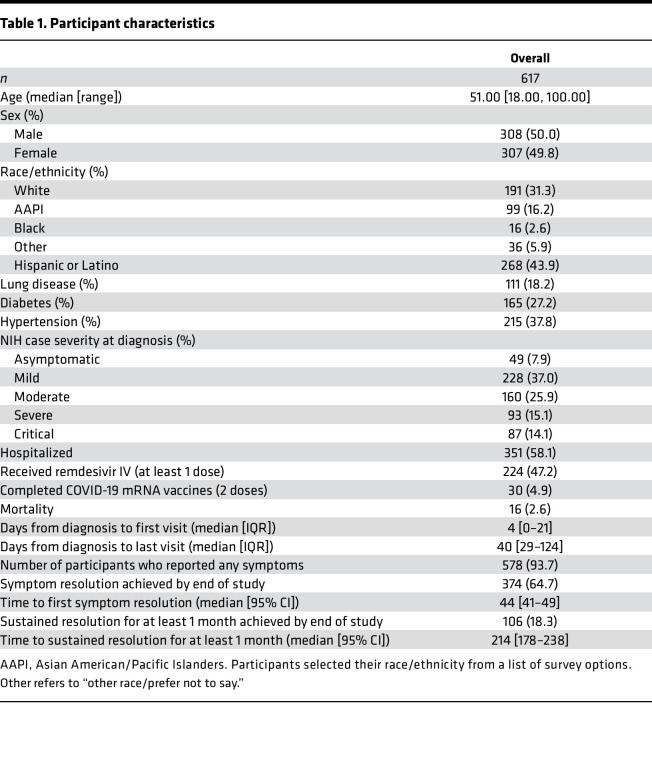
Participant characteristics

**Table 2:**
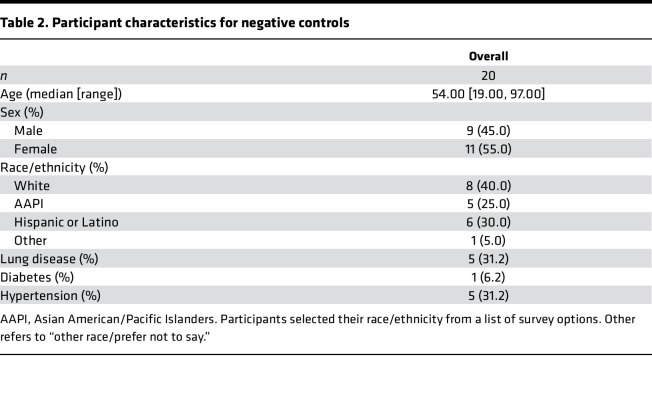
Participant characteristics for negative controls

## Version 1 07/08/2022

Electronic publication

## References

[1] https://covid19.who.int/.

[2] https://www.nih.gov/about-nih/who-we-are/nih-director/statements/nih-launches-new-initiative-study-long.

[3] https://www.cdc.gov/coronavirus/2019-ncov/hcp/clinical-care/post-covid-conditions.html.

[4] Nasserie T (2021). Assessment of the frequency and variety of persistent symptoms among patients with COVID-19: a systematic review. JAMA Netw Open.

[5] Groff D (2021). Short-term and long-term rates of postacute sequelae of SARS-CoV-2 infection: a systematic review. JAMA Netw Open.

[6] Nalbandian A (2021). Post-acute COVID-19 syndrome. Nat Med.

[7] Lund LC (2021). Post-acute effects of SARS-CoV-2 infection in individuals not requiring hospital admission: a Danish population-based cohort study. Lancet Infect Dis.

[8] Wang H (2021). SARS-CoV-2 nucleocapsid plasma antigen for diagnosis and monitoring of COVID-19. Clin Chem.

[9] Simonnet A (2021). High incidence of Epstein-Barr virus, cytomegalovirus, and human-herpes virus-6 reactivations in critically ill patients with COVID-19. Infect Dis Now.

[10] Su Y (2022). Multiple early factors anticipate post-acute COVID-19 sequelae. Cell.

[11] Rentsch CT (2020). Patterns of COVID-19 testing and mortality by race and ethnicity among United States veterans: a nationwide cohort study. PLoS Med.

[12] Graham EL (2021). Persistent neurologic symptoms and cognitive dysfunction in non-hospitalized Covid-19 “long haulers”. Ann Clin Transl Neurol.

[14] Logue JK (2021). Sequelae in adults at 6 months after COVID-19 infection. JAMA Netw Open.

[15] Sudre CH (2021). Attributes and predictors of long COVID. Nat Med.

[16] Bliddal S (2021). Acute and persistent symptoms in non-hospitalized PCR-confirmed COVID-19 patients. Sci Rep.

[18] Augustin M (2021). Post-COVID syndrome in non-hospitalised patients with COVID-19: a longitudinal prospective cohort study. Lancet Reg Health Eur.

[19] Magnus P (2015). Chronic fatigue syndrome/myalgic encephalomyelitis (CFS/ME) is associated with pandemic influenza infection, but not with an adjuvanted pandemic influenza vaccine. Vaccine.

[20] Tansey CM (2007). One-year outcomes and health care utilization in survivors of severe acute respiratory syndrome. Arch Intern Med.

[21] Lam MH (2009). Mental morbidities and chronic fatigue in severe acute respiratory syndrome survivors: long-term follow-up. Arch Intern Med.

[22] Sigfrid L (2021). Long Covid in adults discharged from UK hospitals after Covid-19: a prospective, multicentre cohort study using the ISARIC WHO Clinical Characterisation Protocol. medRxiv.

[23] Eggert LE (2022). Asthma phenotypes, associated comorbidities, and long-term symptoms in COVID-19. Allergy.

[24] Huang C (2021). 6-month consequences of COVID-19 in patients discharged from hospital: a cohort study. Lancet.

[25] Al-Aly Z (2021). High-dimensional characterization of post-acute sequelae of COVID-19. Nature.

[26] Hogan CA (2021). High frequency of SARS-CoV-2 RNAemia and association with severe disease. Clin Infect Dis.

[27] Pinsky BA, Hogan CA (2021). Carving out a niche for severe acute respiratory syndrome coronavirus 2 (SARS-CoV-2) plasma RNA testing. Clin Infect Dis.

[28] Schmidt C (2021). COVID-19 long haulers. Nat Biotechnol.

[29] Gaebler C (2021). Evolution of antibody immunity to SARS-CoV-2. Nature.

[30] Tarhini H (2021). Long-term severe acute respiratory syndrome coronavirus 2 (SARS-CoV-2) infectiousness among three immunocompromised patients: from prolonged viral shedding to SARS-CoV-2 superinfection. J Infect Dis.

[31] Van Elslande J (2021). Longitudinal follow-up of IgG anti-nucleocapsid antibodies in SARS-CoV-2 infected patients up to eight months after infection. J Clin Virol.

[32] Zhao P (2005). Immune responses against SARS-coronavirus nucleocapsid protein induced by DNA vaccine. Virology.

[33] Addetia A (2020). Neutralizing antibodies correlate with protection from SARS-CoV-2 in humans during a fishery vessel outbreak with a high attack rate. J Clin Microbiol.

[34] Lumley SF (2021). Antibody status and incidence of SARS-CoV-2 infection in health care workers. N Engl J Med.

[35] Choe PG (2021). Antibody responses 8 months after asymptomatic or mild SARS-CoV-2 infection. Emerg Infect Dis.

[36] Ibarrondo FJ (2020). Rapid decay of anti-SARS-CoV-2 antibodies in persons with mild Covid-19. N Engl J Med.

[37] Turner JS (2021). SARS-CoV-2 infection induces long-lived bone marrow plasma cells in humans. Nature.

[38] Chen J (2017). Long term outcomes in survivors of epidemic Influenza A (H7N9) virus infection. Sci Rep.

[39] Suzuki M (2007). Identification of viruses in patients with postviral olfactory dysfunction. Laryngoscope.

[40] https://www.covid19treatmentguidelines.nih.gov/overview/clinical-spectrum/.

[41] Grandjean L (2021). Long-term persistence of spike antibody and predictive modeling of antibody dynamics following infection with SARS-CoV-2. Clin Infect Dis.

[42] Johnson M (2020). Evaluation of a novel multiplexed assay for determining IgG levels and functional activity to SARS-CoV-2. J Clin Virol.

