# Supplementary material for: Anti-nucleocapsid antibody levels and pulmonary comorbid conditions are linked to post–COVID-19 syndrome
Source: JCI Insight. 2022 Jul 8;7(13):e156713. doi: 10.1172/jci.insight.156713 (PMC9310538; doi:10.1172/jci.insight.156713)
Supplement: Supplemental data [file jciinsight-7-156713-s081.pdf]

Supplementary Figures

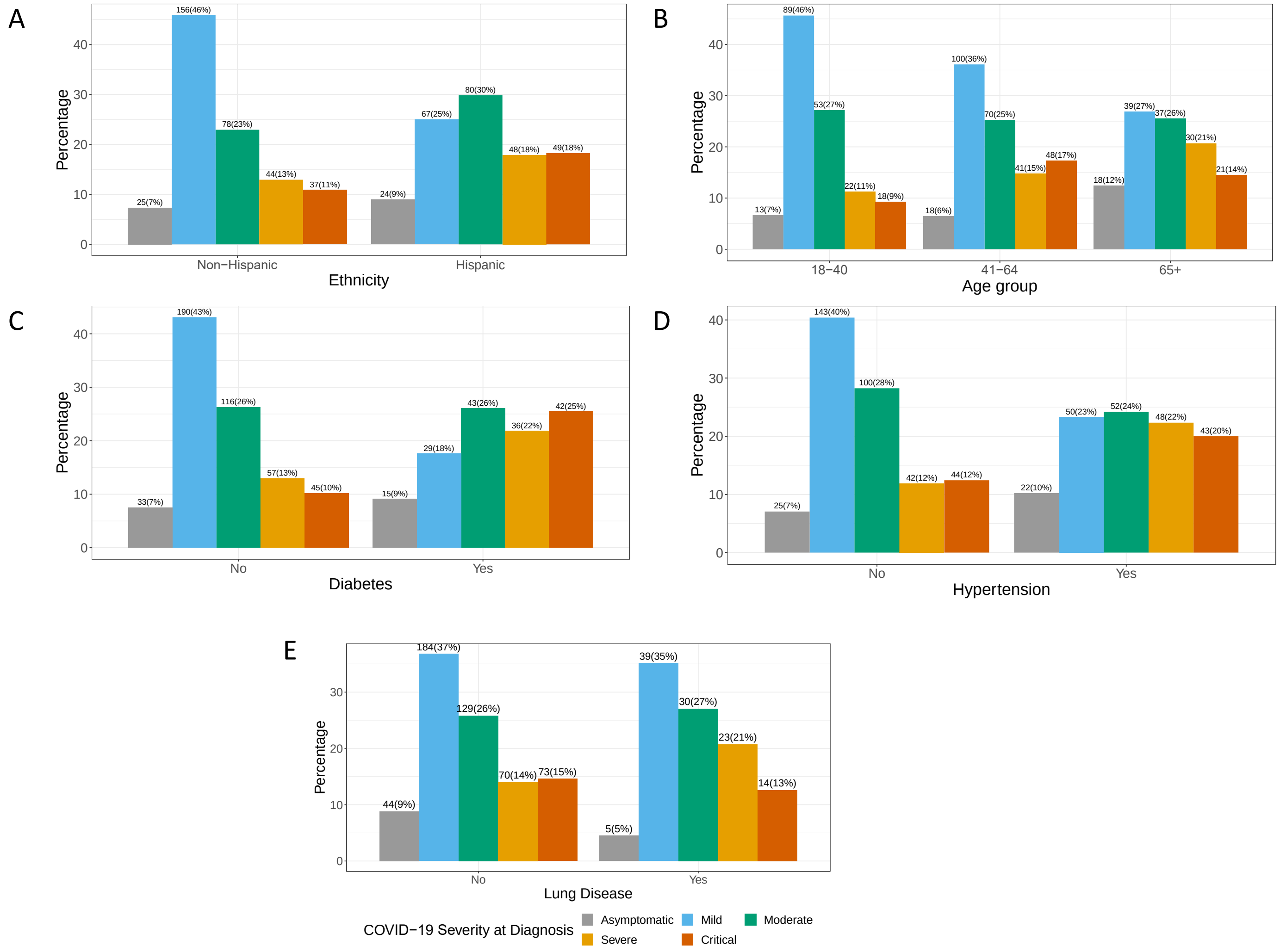

Supplemental Figure 1. Association between COVID-19 case severity at diagnosis and baseline demographics and comorbidities. Numbers and percentages of COVID-19 case severity at diagnosis stratified by (A) race/ethnicity, (B) age group, (C) diabetes, (D) hypertension, and (E) lung disease (i.e. asthma or chronic obstructive pulmonary disease prior to the diagnosis of COVID)

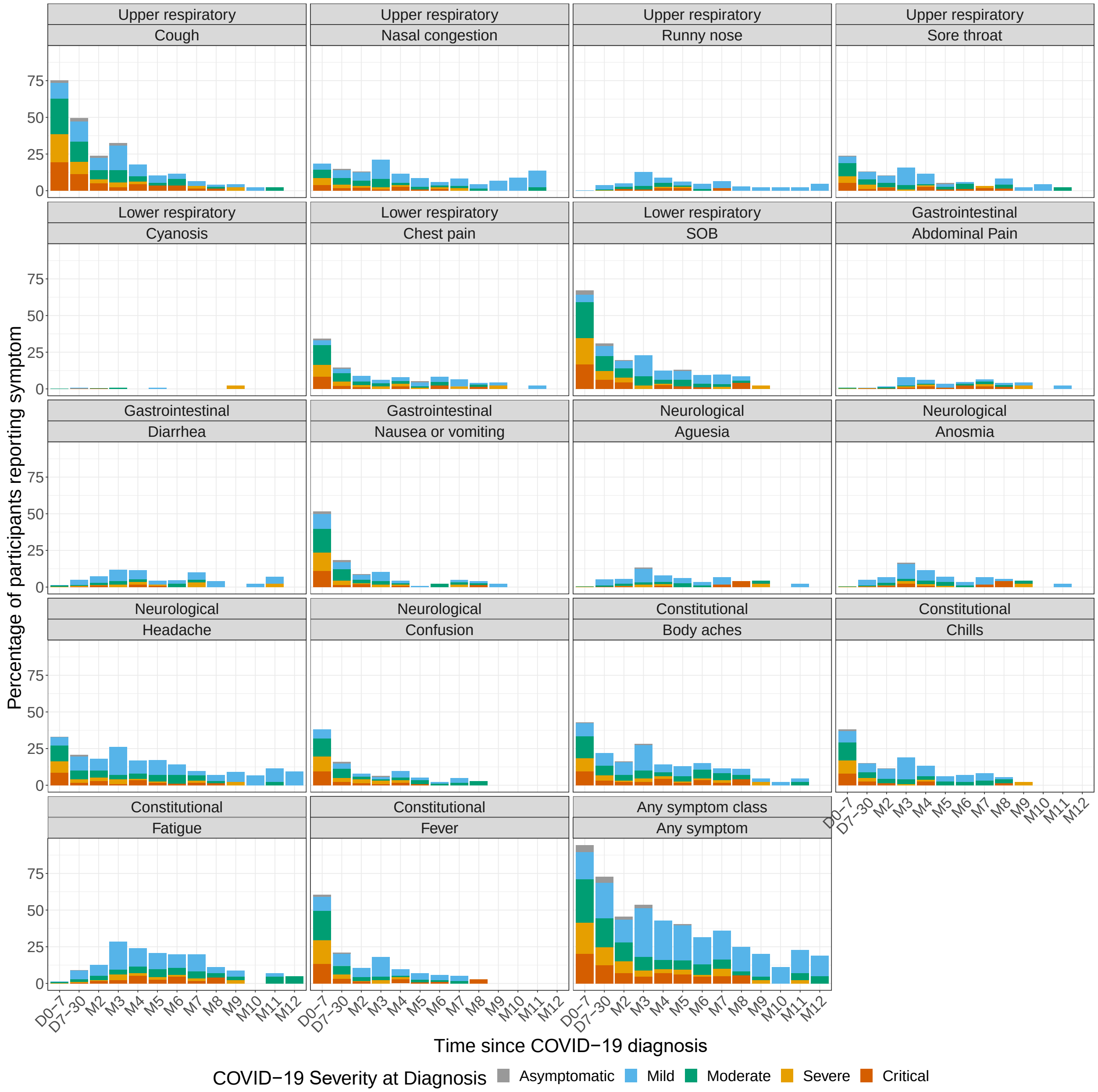

Supplemental Figure 2. Symptom distribution over time since COVID-19 diagnosis stratified by disease severity. We grouped the symptoms into 5 classes based on the organ systems. (1) Upper respiratory symptoms included cough, nasal congestion, runny nose, and sore throat; (2) lower respiratory symptoms included cyanosis, chest pain, and shortness of breath; (3) gastrointestinal symptoms included abdominal pain, diarrhea, nausea or vomiting; (4) neurological symptoms included ageusia, anosmia, headache, and confusion; and (5) constitutional symptoms included body aches, chills, fatigue, and fever. Disease severity was determined by NIH scoring (1). Abbreviations: D: day, M: month, SOB: shortness of breath.

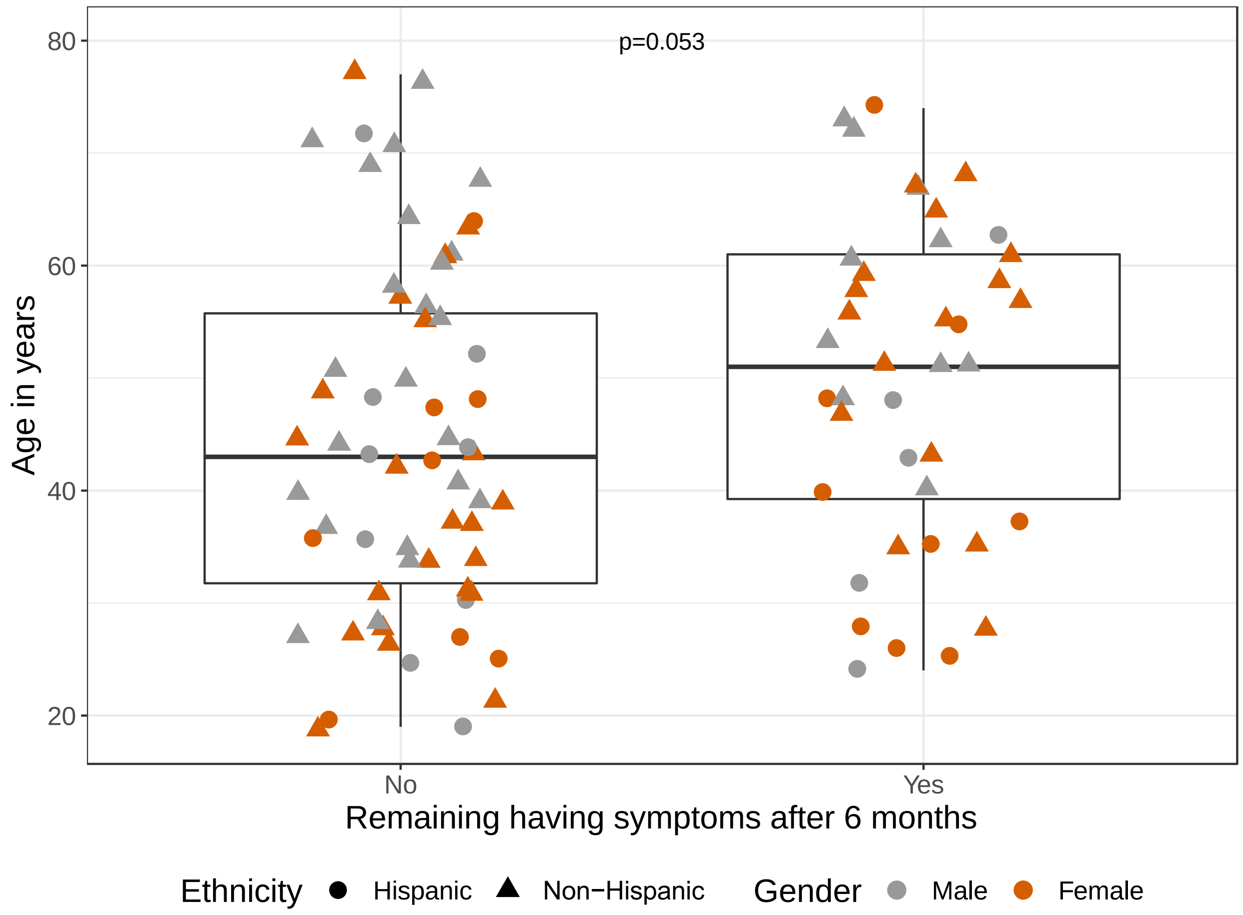

Supplemental Figure 3: Comparison of age groups with and without symptoms after 6 months. P value was based on non-parametric Kruskal-Wallis test.

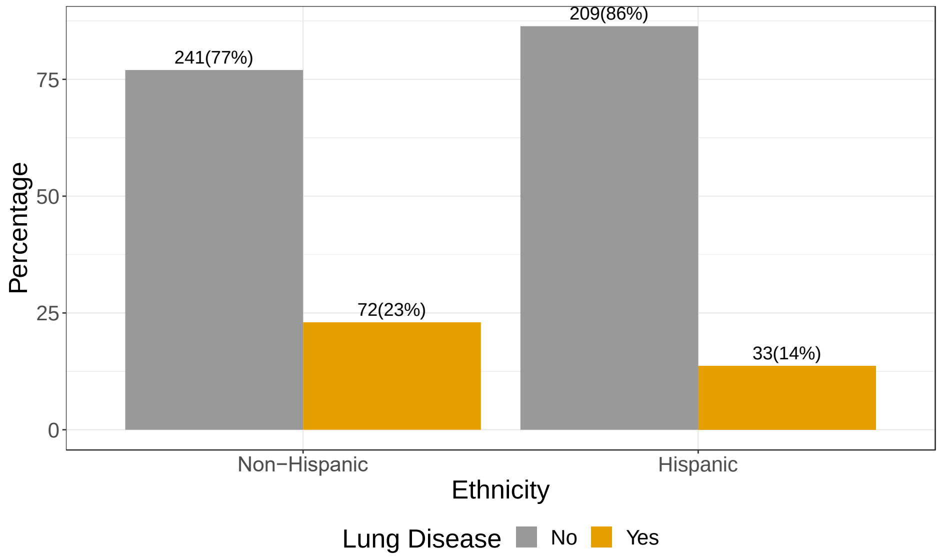

Supplemental Figure 4: Association between Ethnicity and lung disease (i.e. asthma or chronic obstructive pulmonary disease prior to the diagnosis of COVID).

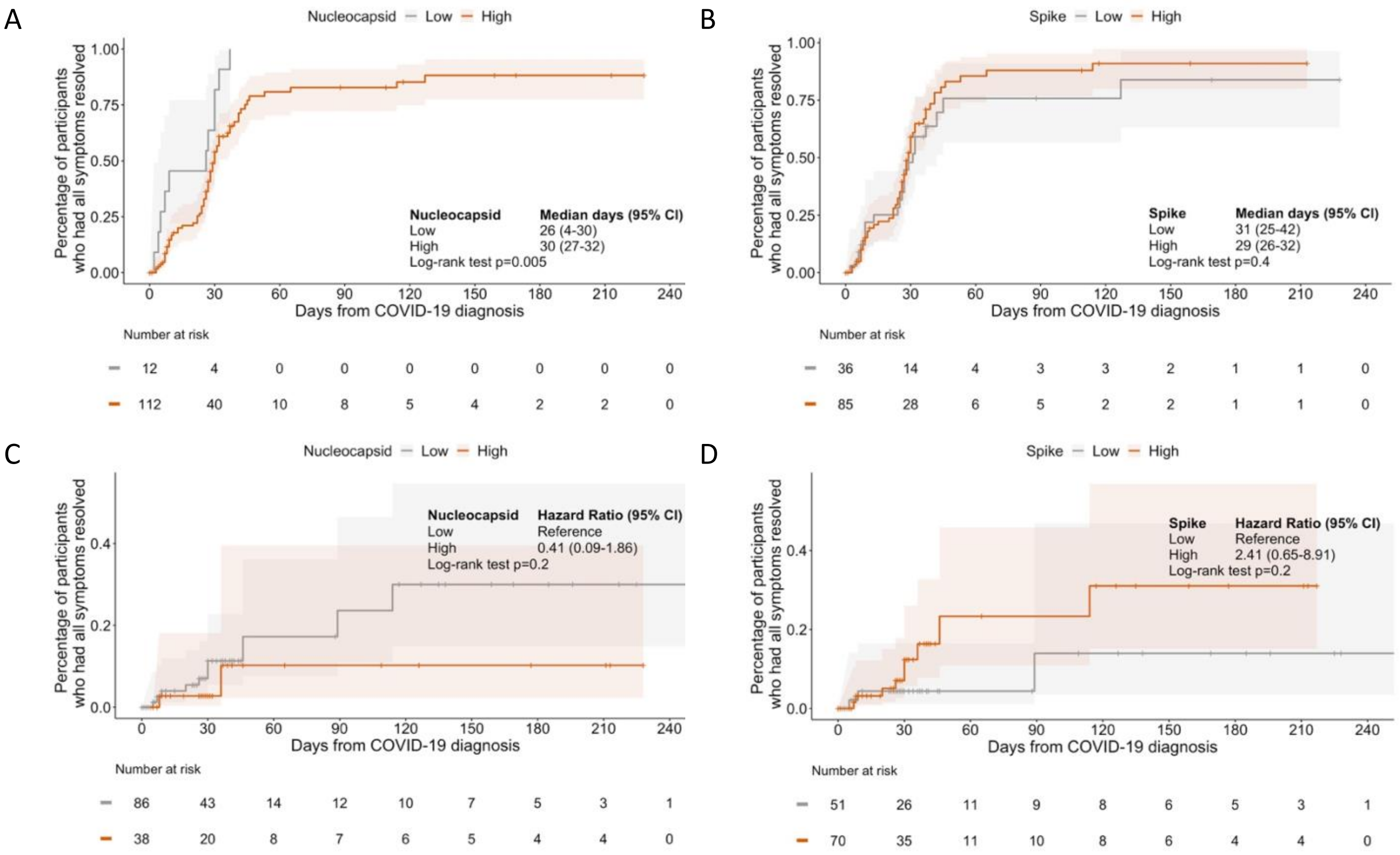

Supplementary Figure 5. Association between Nucleocapsid and Spike protein concentration with time to first and sustained resolution. (A) Kaplan-Meier curves of time to first symptom resolution and corresponding 95% confidence interval bands by antigen nucleocapsid. Low:<= 2153 fg/ml; High: > 2153 fg/ml. (B) Kaplan-Meier curves of time to first symptom resolution and corresponding 95% confidence interval bands by antigen spike. Low:<= 5884 fg/ml; High: >5884 fg/ml. (C) Kaplan-Meier curves of time to sustained symptom resolution and corresponding 95% confidence interval bands by antigen nucleocapsid. Low:<= 2339248 fg/ml; High: > 2339248 fg/ml. (D) Kaplan-Meier curves of time to sustained symptom resolution and corresponding 95% confidence interval bands by antigen spike. Low:<=11546 fg/ml; High: >11546 fg/ml. Participants who did not reach the endpoint are censored at their last visit (represented by the “+”). P values were based on the log-rank test.

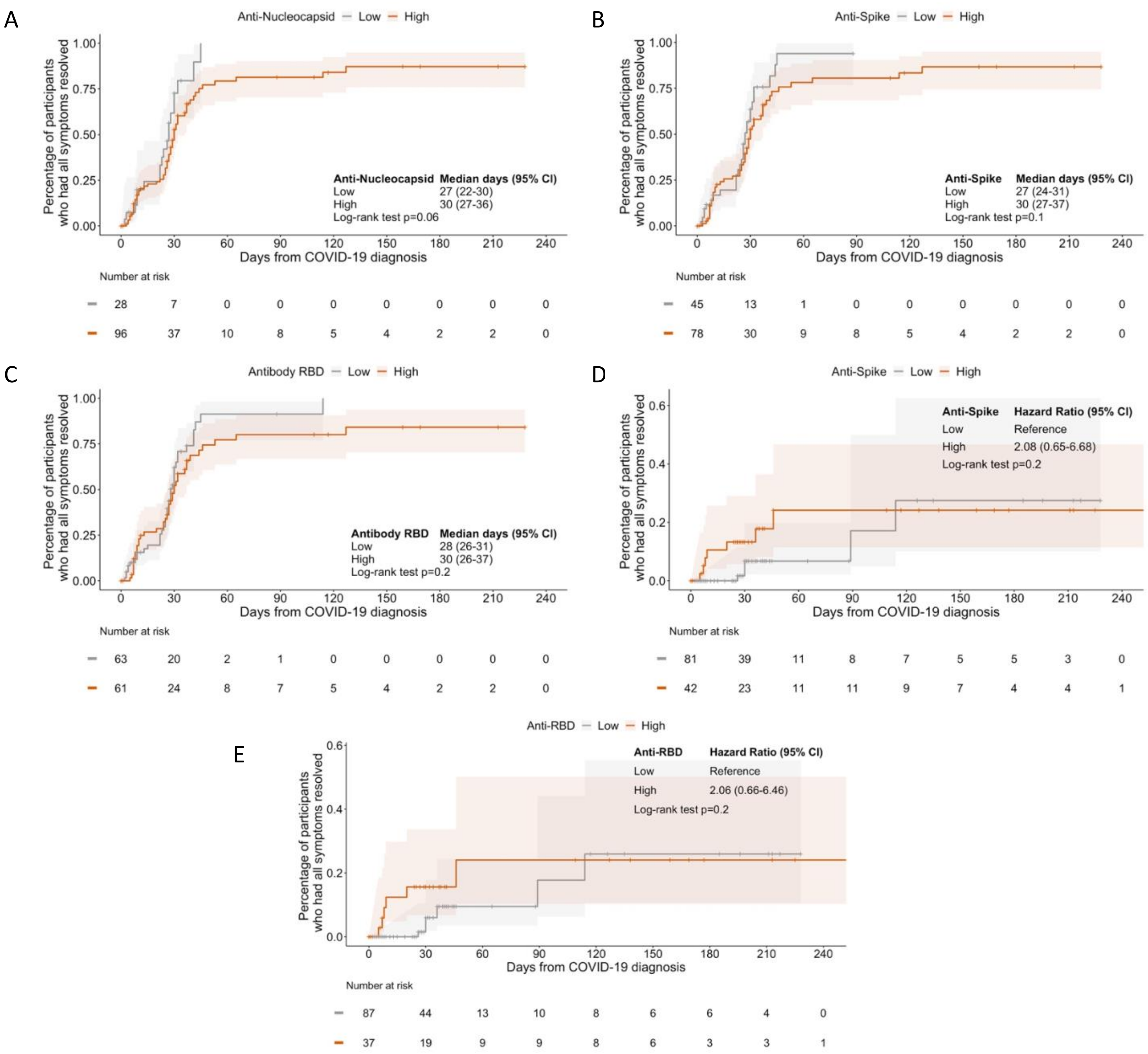

Supplementary Figure ~~5~~6. Association between anti-nucleocapsid, anti-spike, and anti-RBD IgG with time to first and sustained resolution. (A) Kaplan-Meier curves of time to first symptom resolution and corresponding 95% confidence interval bands by anti-nucleocapsid IgG. Low:<=252.222 AU/ml; High: >252.222 AU/ml. (B) Kaplan-Meier curves of time to first symptom resolution and corresponding 95% confidence interval bands by anti-spike IgG. Low:<=440.1426 AU/ml; High: >440.1426 AU/ml. (C) Kaplan-Meier curves of time to first symptom resolution and corresponding 95% confidence interval bands by anti-RBD. Low:<=482.433 AU/ml; High: >482.433 AU/ml. (D) Kaplan-Meier curves of time to sustained symptom resolution and corresponding 95% confidence interval bands by anti-spike. Low:<=6695.098 AU/ml; High: >6695.098 AU/ml. (E) Kaplan-Meier curves of time to

sustained symptom resolution and corresponding 95% confidence interval bands by anti-RBD. Low:<=2279.689 AU/ml; High: >2279.689 AU/ml. Participants who did not reach the endpoint are censored at their last visit (represented by the "+"). P values were based on the log-rank test.

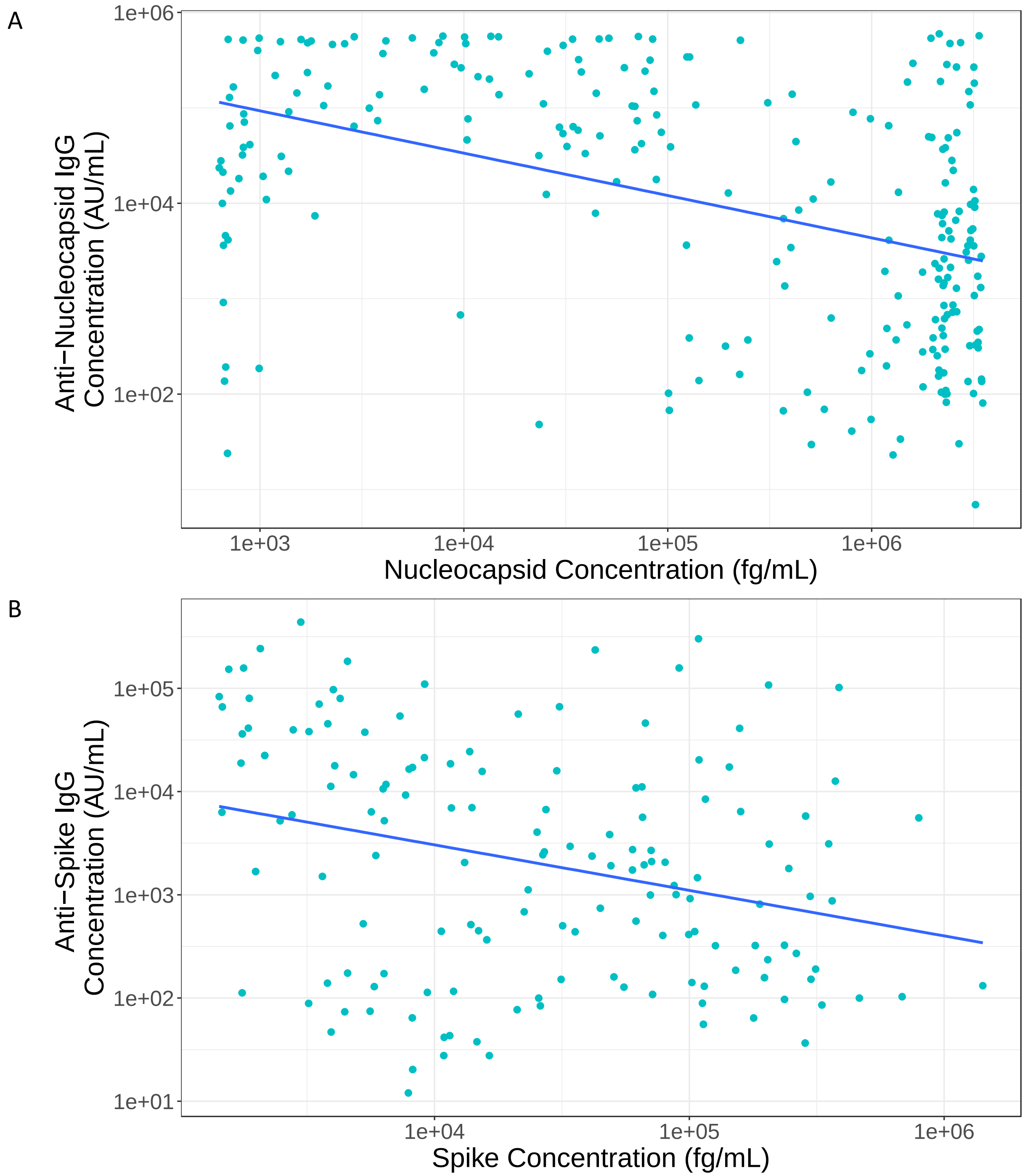

Supplementary Figure ~~6~~7. Association between antigens and antibodies. Correlations between (A) Nucleocapsid protein concentration and anti-Nucleocapsid IgG concentration, and (B) spike protein concentration and anti-Spike IgG concentration. Linear regression lines and corresponding 95% confidence interval bands are shown.

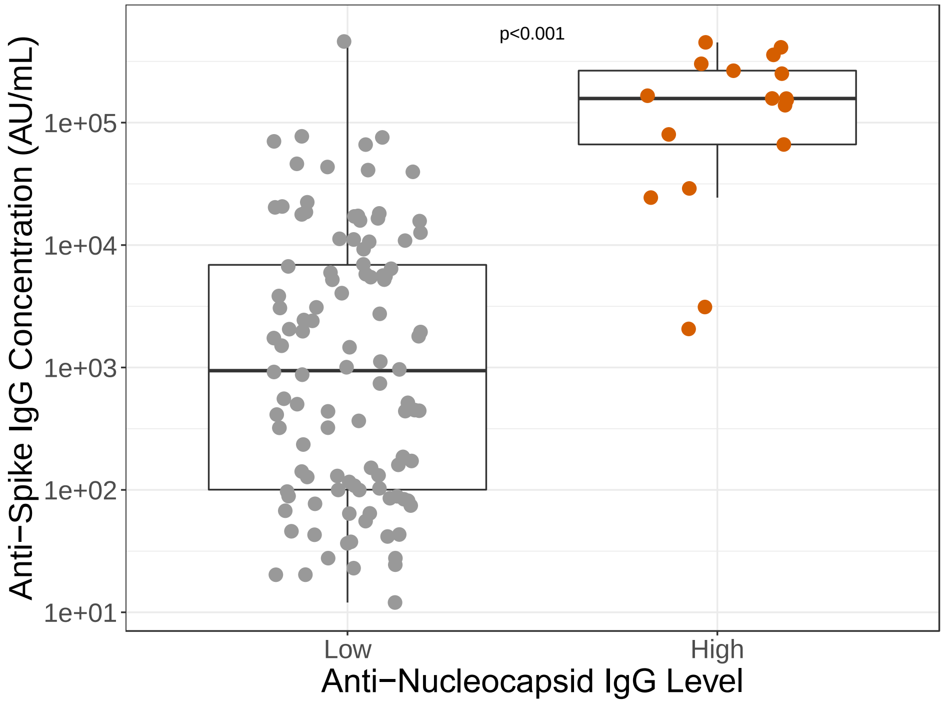

Supplementary Figure 8: Comparison of Anti-Spike IgG concentration between high vs low anti-nucleocapsid IgG levels. Low: <=149452.067AU/ml, high: >149452.067AU/ml. P value was based on non-parametric Kruskal-Wallis test.

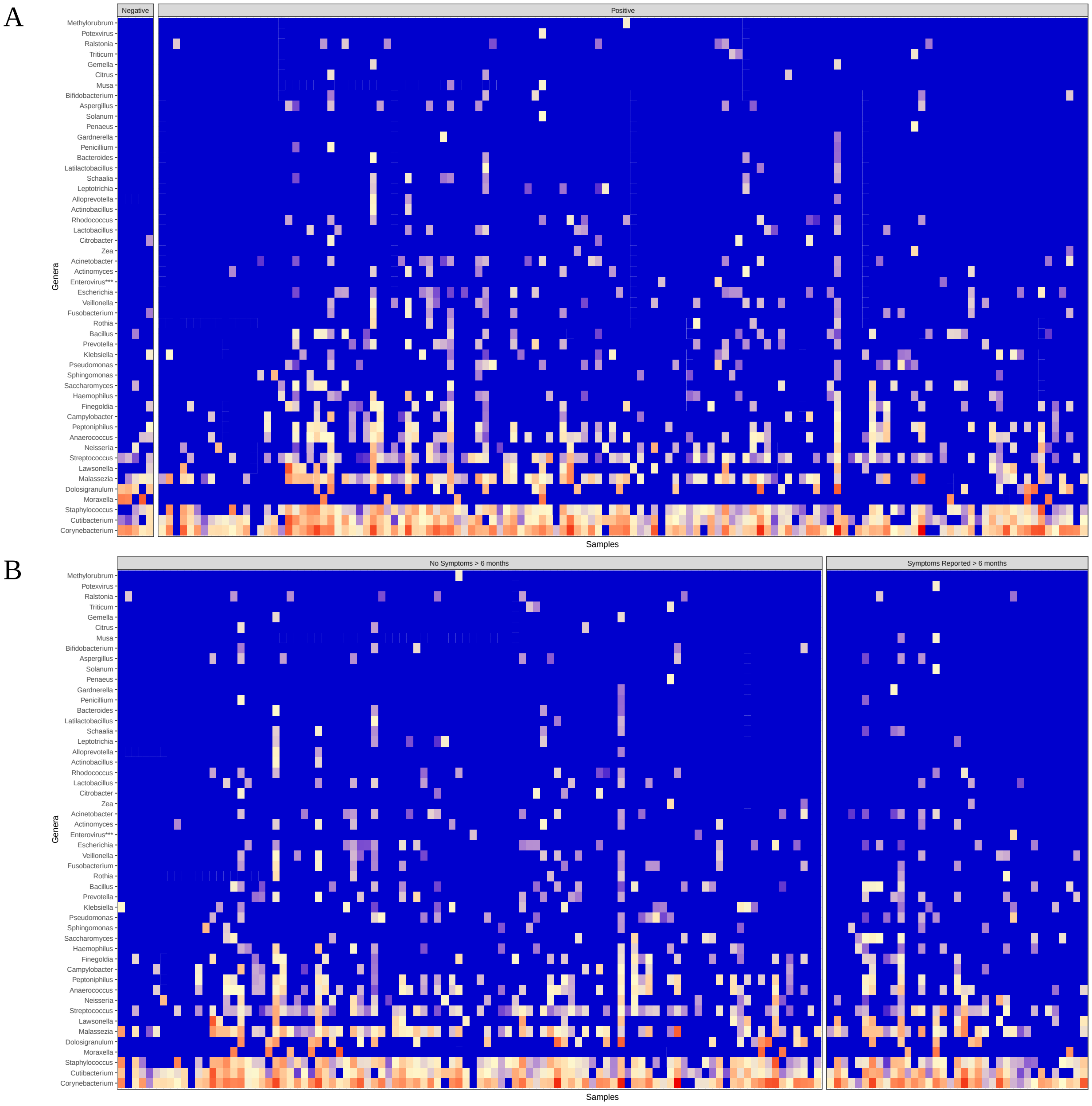

Supplementary Figure 9. A. Heatmap of metagenomic microbiome profiles from COVID-positive and COVID-negative participants. Genus-level microbiome profiles of nasal swabs collected from participants with a diagnosis of COVID-19 (n=132) and individuals with no diagnosis of COVID-19 (n=5) are displayed. Log base 10 transformed NT RPM values for the fifty most abundant genera are shown. Each column represents a single microbiome profile. Each row shows the presence of a specific taxon. The prevalence of each taxon in each sample is shown on a blue:yellow:red ascending scale, with blue representing low or no abundance, yellow representing intermediate abundance and red representing high abundance. Figures were generated using the R package 'ggplot2'. The plot is split into two panels based on COVID-19 status (Positive/Negative), which is annotated in a single row at the top of the heatmap. The single viral genus in the plot is highlighted using "***". **B.** Heatmap of metagenomic microbiome profiles from participants with and without long COVID. Genus-level microbiome profiles of nasal swabs

collected from COVID positive participants experiencing symptoms of COVID-19 for longer than 6 months (long COVID) (n=100) and those whose COVID-19 symptoms lasted less than 6 months (n=37) are displayed. Log base 10 transformed NT RPM values for the fifty most abundant genera are shown. Each column represents a single microbiome profile. Each row shows the presence of a specific taxon. The prevalence of each taxon in each sample is shown on a blue:yellow:red ascending scale, with blue representing low or no abundance, yellow representing intermediate abundance and red representing high abundance. Figures were generated using the R package 'ggplot2'. The plot is split into two panels based on long COVID status (No Symptoms > 6 months/ Symptoms Reported > 6 months), which is annotated in a single row at the top of the heatmap. The single viral genus in the plot is highlighted using "***".

REFERENCES

1. Health NIo. Clinical Spectrum of SARS-CoV-2 Infection. 2021; https://www.covid19treatmentguidelines.nih.gov/overview/clinical-spectrum/. Accessed August 1, 2021.

Supplementary Tables

Supplementary Tables

Supplementary Table 1. Number of participants per each sequential follow-up period. Participants could have completed multiple blood draws and/or questionnaires within a given time period. *=those who consented for blood draw with sufficient volume for testing.

Days since diagnosis	Number of individual participants with completed questionnaires	Median age and range (years)	Percentage of tho who were asymptomatic at diagnosis	Percentage with mild, moderate, severe, and critical symptoms	Number of completed questionnaires	Number of individual participants for biomarker testing based on convenience sample*	Number of antigen/anti body tests based on convenience sample*
[0, 7]	369	51 (19-100)	7%	21%, 30%, 22%, 20%	552	136	301
(7, 30)	361	51 (19-100)	8%	29%, 29%, 18%, 16%	477	67	104
[30, 90)	411	51 (18-100)	8%	40%, 25%, 16%, 11%	690	131	196
[90, 180)	166	48 (18-90)	4%	66%, 13%, 9%, 8%	742	90	127
[180, 270)	103	45 (19-77)	2%	67%, 14%, 9%, 9%	466	53	98
[270, 370)	59	45 (20-77)	2%	86%, 8%, 2%, 2%	280	26	29

Supplemental Table 2. Participant characteristics stratified by presence or lack of symptoms at 6 months post diagnosis.

	**Participants with symptoms data 6 months post diagnosis**		
	**No**	**Yes**	**P***
n	63	42	
Age (median [range])	43.00 [19.00, 77.00]	51.00 [24.00, 74.00]	0.053
Female	31 (49.2)	27 (64.3)	0.186
Race/Ethnicity (%)			0.474
White	25 (40.3)	19 (47.5)	
AAPI	18 (29.0)	6 (15.0)	
Black	1 (1.6)	0 (0.0)	
Other	1 (1.6)	1 (2.5)	
Hispanic	17 (27.4)	14 (35.0)	
NIH Case Severity at Diagnosis (%)			0.347
Asymptomatic	2 (3.2)	0 (0.0)	
Mild	45 (71.4)	26 (61.9)	
Moderate	8 (12.7)	6 (14.3)	
Severe	5 (7.9)	4 (9.5)	
Critical	3 (4.8)	6 (14.3)	

*P value was based on Chi-square test and Kruskal-Wallis test for categorical and numeric variables respectively.

Supplemental Table 3: Participants characteristics from the convenience sample for those who had a blood draw with sufficient blood volume for biomarker testing.

	**Variable**	**Overall**
n		304
Age (median [range])		48.00 [19.00, 94.00]
Gender (%)	Male	153 (50.5)
	Female	150 (49.5)
Race (%)	White	102 (34.0)
	AAPI	51 (17.0)
	Black	7 (2.3)
	Other	8 (2.7)
	Hispanic	132 (44.0)
NIH Case Severity at Diagnosis (%)	Asymptomatic	18 (5.9)
	Mild	132 (43.4)
	Moderate	64 (21.1)
	Severe	46 (15.1)
	Critical	44 (14.5)

Supplemental Table 6: Summary of viral signatures detected through mNGS analysis

Stanford ID	COVID status	Total Reads	Non-host Reads	Genus	NT RPM
55689-0093-Month2*	Positive	100396386	3875134	Propionibacterium virus P1.1	8.124537879
55689-0035-Month3	Positive	11881906	1108722	Malassezia restricta virus MrV40S	75.27901666
55689-0010-Month2*	Positive	315530	80588	Staphylococcus virus SEP9	135.2744138
55689-0150 V1	Negative	1849152	23420	Youcai mosaic virus	13.01411164
55689-0010 V6*	Positive	2907170	306572	Malassezia restricta virus MrV40S	15.87433052
55689-0032 V6	Positive	2690072	67890	Bell pepper alphaendornavirus	23.42382233
				Tomato mosaic virus	6.800464547
55689-0022 V6	Positive	4467578	3820	Tomato brown rugose fruit virus	9.4095001
55689-0056 V6*	Positive	3131042	321388	Pepino mosaic virus	457.2414565
55689-0009 V7	Positive	2775996	88504	Tomato brown rugose fruit virus	11.61682203
55689-0074 V6*	Positive	355860	7026	Shenzhen dicistro-like virus	159.0764476
55689-0105 V6	Positive	190752	118	Rhinovirus C	138.3067004
55689-0058 V7*	Positive	2778356	22386	Rhinovirus A	1757.19866
55689-0185-Month3*	Positive	4054606	1160	Severe acute respiratory syndrome-related coronavirus	5.928133241
55689-0222-Month2	Positive	2942450	11320	uncultured Caudovirales phage	8.184210203
55689-0072-V8	Positive	4024180	157070	Gammapapillomavirus 27	67.91281616
55689-0217-D0	Positive	2969004	1381816	Panicum mosaic virus	6.261623138
55689-0172-D7	Positive	2372658	1254364	Malassezia restricta virus MrV40L	12.14167952
55689-0211-D0	Positive	2228410	2402	Rhinovirus B	13.48772886
55689-0033 V6	Positive	1173184	153090	Prokaryotic dsDNA virus sp.	27.6454062
OHC007-V06	Positive	3438828	148578	Malassezia restricta virus MrV40S	17.49331318
OHC018-V09	Positive	134352	3568	Tomato brown rugose fruit virus	245.8701498
				Tomato mottle mosaic virus	107.5681905
				Shenzhen reo-like virus 1	291.9708029

*These samples belong to participants who reported symptoms beyond 6 months
FDR-adjusted P value for each <1e-314

Anti-Nucleocapsid antibody levels and pulmonary co-morbid conditions are linked to Post COVID-19 syndrome

SUPPLEMENTARY MATERIALS

Statistical analysis

Participant's characteristics

Descriptive statistics were used for participant demographics and clinical characteristics, including median, range, and interquartile range for continuous variables and counts and percentages for categorical variables. The associations between COVID-19 severity and ethnicity and comorbidities were illustrated as bar charts and analyzed using chi-square or Fisher's test whenever appropriate. Severity was defined by NIH criteria (1).

Long-term symptoms

The distributions of symptoms and symptoms classes over time were illustrated using stacked bar graphs. The co-occurrence between symptoms at month one, two, three and six after diagnosis were estimated by Jaccard similarity index. Specifically, the index between two symptoms was defined as the number of participants who reported both symptoms divided by the number of participants who reported either symptom. Generalized linear mixed-effects model (GLMM) for the negative binomial family was fitted on the average number of symptoms over time as a function of age, gender, severity, race and ethnicity, diabetes, lung disease, hypertension, months after diagnosis, with a participant level random effect and a quadratic term of time. P values, odds ratio, and corresponding 95% confidence intervals were reported.

Associations between long-term symptoms

For each symptom class, we further applied GLMM to estimate its trajectory of associations with other symptom classes at diagnosis or at follow-up month. Specifically, we fitted a GLMM model with binomial family by regressing each of the other symptom classes, time of the visit, and interaction of them on the binary symptom outcome in a longitudinal dataset. A random intercept that accounted for the correlations within a patient was included in the model. A separate GLMM model was then performed to assess the associations between the same symptom classes at diagnosis and its corresponding follow up months. Different from the first set of models, the independent variable was the same as the dependent symptom class although it was measured at diagnosis and was repeated for each follow-up visit. Initial visits at diagnosis were not included here. Estimated marginal means (EMM) that assessed the associations at each time period were calculated from model using 'emmeans' package in R. Time of the visits was treated as a continuous measure for all the above analysis to assume linear trajectory of the associations with time. To summarize the results, we visualized the EMMs as log odds ratios, the corresponding standard error and p values over time with heatmaps.

Time to symptom resolution

Kaplan-Meier survival methods were used to assess the association between the time from COVID-19 diagnosis to symptom resolution with disease severity, comorbidities, race and ethnicity group, and CMV exposure status. Participants who did not reach the endpoints were censored at the last visit. P values from the log-rank test were reported to test whether time to symptom resolution differed across groups.

Antigen and antibody data

The linear mixed-effect model was fitted on the concentrations of antigens and antibodies over time as a function of severity, with a participant level random effect and a quadratic term of time. The associations between antigen N and anti-N IgG, and between antigen S and anti-S IgG were assessed by the Spearman's rank correlation test, and only antigen N and S that were above the detection thresholds were included, which was defined as mean mean + 3SD signals from 80 pre-pandemic samples (antigen N: 629fg/ml; antigen S: 1352 fg/ml). The log transformation was applied for the antigen and antibody data when appropriate. We further explored the effect of antigens and antibodies in the first week on the time to the first and sustained symptom resolution. Log transformation was applied for the continuous antigen and antibody data when analyzed in the Cox regression. The sensitivity analysis was further performed to explore whether there were optimal cutpoints that can create the largest survival separation between groups. Optimal cutpoints were determined using the maximally selected rank statistics from the 'maxstat' R package, which is an outcome-oriented method providing a value of a cutoff that corresponds to the most significant relation with outcome.

Complete case was used to handle missing data that only includes participants for which we have no missing data on the variables of interest. Tests were two-sided and conducted at the 0.05 level of significance. All analyses were conducted using R software v4·0·3.

Laboratory methods:

Meso Scale Discovery Electrochemiluminescence (MSD ECL) assays to detect SARS-CoV-2 Nucleocapsid and Spike Antigens

SARS-CoV-2 nucleocapsid and spike antigens were quantified using ultra-sensitive antigen

capture immunoassays, S-PLEX SARS-CoV-2 N Kit (catalog #K150ADHS, MSD) and S-PLEX SARS-CoV-2 Spike Kit (catalog #K150ADJS, MSD), performed according to manufacturer instructions. First, S-PLEX 96-well SECTOR plates were coated with biotinylated capture antibodies and incubated either for 1 hour at room temperature (RT) or overnight at 4°C. Immediately after plates were blocked using the MSD blocking solution, 25 µL of sample was added to each well. A 7-point calibration curve and negative control consisting of assay diluent was run in duplicate on each plate. Following sample incubation at RT for 1.5 hours, detection antibody was added (MSD TURBO-BOOST™ Detection Antibody) and incubated for 1 hour at RT, and an enhancement step was performed by using the MSD S-PLEX Enhance solution with a 30-minute incubation at RT. Subsequently, detection solution (TURBO-TAG™ Detection Solution) was added. After incubation for 1 hour at 27°C and addition of MSD GOLD™ Read Buffer B, the plates were read using a MESO QuickPlex SQ 120 reader. All incubations were performed in a plate shaker at 700 rotations per minute. Each incubation step was followed by a washing step, consisting of three washes in MSD Tris Wash Buffer using the BioTek 405 Select automated 96-well plate washer (BioTek, Winooski, VT). Raw signal was converted to a concentration based on linear regression to the 7-point calibration curve, which was run in duplicate on each plate.

Nucleocapsid antigen was interpreted as positive if the concentration exceeded 2.80 $\log_{10}$ fg/mL. This threshold represented the 99th percentile or mean+2.33*SD concentration of 80 pre-pandemic plasma samples from healthy blood donors. Spike antigen was interpreted as positive if the concentration exceeded 3.13 $\log_{10}$ fg/mL. This threshold represented the 99th percentile or mean+2.33*SD concentration of a separate set of 80 pre-pandemic plasma samples from healthy

blood donors. For samples with original concentrations above the upper limit of quantitation of 6 $\log_{10}$ fg/mL, the assay was repeated on a 1:100 dilution of the original sample in phosphate buffered saline.

Meso Scale Discovery Electrochemiluminescence (MSD ECL) assays to detect anti-SARS-CoV-2 IgG

Longitudinal plasma samples collected from COVID-19 patients were tested with MSD ECL MULTI-SPOT 96-well plate SARS-CoV-2 assays and instrumentation by following the manufacturer's recommendations. Briefly, V-PLEX Coronavirus Panel 4 kits were used to detect IgG plasma antibodies to SARS-CoV-2 spike, receptor-binding domain (RBD), and nucleocapsid antigens. Plasma samples were analyzed at a 1:5'000 dilution, detected with SULFO-TAG ECL-labelled anti-human IgG and read with a MESO QuickPlex SQ 120 instrument. 160 plasma samples were analyzed twice in two independent experiments to assess inter-assay variation. Each plate contained duplicates of a 7-point calibration curve with serial dilution of a reference standard (provided with the kit) and a blank control well containing diluent only. Calibration curves were used to calculate arbitrary unit concentrations of IgG antibodies (MSD AU/mL) by backfitting ECL signals measured for each sample to the calibration curve. The cutoff value for seropositivity was based on the mean + 3 standard deviation signals from 37 pre-pandemic samples tested in duplicate.

Nasal Swab Collection and Testing

For the initial diagnosis, a clinician-collected lower nasal swab (Puritan Sterile Foam Tipped Applicator; Puritan Medical Products) was used. The specimen was placed in viral transport medium (M4RT Transport; Remel MicroTest), stored in a cooler, and delivered to the Clinical

Laboratory Improvement Amendments (CLIA)-accredited Stanford Clinical Virology Laboratory for diagnostic testing.

A variety of nucleic acid amplification methods were used including 1) an emergency use authorized, laboratory-developed reverse transcription quantitative polymerase chain reaction (RT-qPCR) targeting the envelope gene on the Rotor-Gene Q (Qiagen, Germantown, MD) (2, 3); 2) Xpert Xpress SARS-CoV-2 (Cepheid, Sunnyvale, CA), a rapid RT-qPCR method targeting both envelope and nucleocapsid genes (4), 3) Panther Fusion SARS-CoV-2 (Hologic, Marlborough, MA), a high-throughput RT-qPCR method targeting two regions of open reading frame 1ab (ORF1ab) (5, 6). All assays were conducted according to manufacturer and emergency use authorization instructions.

For the longitudinal visits, nasal mid-turbinate self-swabs were collected in sterile 3 ml PBS or 1 ml 1X DNA/RNA shield (Zymo Research, Tustin, CA), and frozen down at -80°C until testing. On thawing prior to testing: (i) for the swabs collected in PBS, a 200 μl aliquot was taken and mixed with 200 μl of 2X DNA/RNA shield in a sterile 10 ml screw cap tubes (Sarstedt, Germany), whereas, (ii) for the swabs collected in 1X DNA/RNA shield a 400 μl aliquot was transferred into sterile 10 ml screw cap tubes.

The nasal swabs underwent RNA extraction and qRTPCR assays for the presence of SARS-CoV-2 in an emergency-use authorized and CLIA-approved COVID-19 testing facility. A detailed description of methods and SARS-CoV-2 detection assays have been previously described (7). Samples were treated as research-only and non-reportable.

Sequencing library preparation

Sequencing library preps were carried out for all samples that were COVID positive by qPCR assay (n = 13) and all samples that were COVID negative by qPCR assay, but showed $C_t < 33.33$ for host RNAseP control included in the qPCR assay (n= 260). For each library prep, 10 μL of the total RNA extracted from nasal swabs that remained after COVID qPCR assays was incubated with recombinant RNAse-free DNase (Qiagen, USA) for 15min at room temperature, purified with SPRI beads, eluted in 10 uL of nuclease-free H_2O, and then dried down in a 384 well plate. A set of 8 water samples and a duplicated 8-fold dilution series of total RNA extracted from Hela cells were included on the 384 well plate as negative and positive controls for the RNAseq library preparation, respectively.

A modified version of a high throughput miniaturized NEBNext Ultra II RNA sequencing library preparation method (8) was used to prepare RNAseq libraries. Briefly, the dehydrated RNAs were resuspended in a final volume of 1.1uL containing standard NEBNext Ultra II RNAseq library prep reagents (New England Biolabs, Ipswich MA) for RNA fragmentation, plus 25pg of external RNA consortium control spike-ins (ERCCs) (Invitrogen-Thermo Fisher, USA), and 0.1 uL a 1:10 dilution of FastSelect human rRNA depletion reagent (Qiagen, USA). This reaction mixture was first incubated for 4 min at 94°C to fragment the RNA, followed immediately by a series of additional incubation steps required for human rRNA depletion (2 min at 75°C, 2 min at 70°C, 2 min at 65°C, 2 min at 60°C, 2min at 55°C, 5 min at 37C, and 5min at 25°C). All subsequent downstream steps for sequencing library prep were performed as previously described (Mayday MY et al, 2019), using a 1:400 dilution of the NEBNext adapter reagent, and a total of 18 cycles of index PCR.

Prior to sequencing, individual library preps were spot-checked and quantified via gel electrophoresis with High Sensitivity DNA ScreenTape assays on an Agilent Tape Station

System. Based on failure to yield a detectable signal in this assay, 64 of the COVID negative longitudinal nasal swab sample libraries with qPCR C_t > 31 for host RNAseP transcript and 6 of the longitudinal COVID positive nasal swab sample libraries were excluded for further analysis. A final set of 203 individual library preps were pooled for further mNGS sequence analysis. Raw fastq.gz files for these 203 are deposited in NCBI Bioproject PRJNA817002.

Sequencing

To assess the yield and quality of sequence across the library preps, an equivolume pool containing 1 uL from each of the 203 individual libraries was sequenced on an Illumina MiSeq platform (paired-end, 2 x 146bp format). This yielded 14M reads to QC the yield of reads, sequence quality and complexity, and host rRNA depletion in each library prep (see below). Read counts for individual library preps from the MiSeq run were used to generate an approximately equimolar library pool for a subsequent paired-end 2 x 146bp sequencing run for mNGS analysis on an Illumina NextSeq2000 platform. This yielded a final dataset consisting of 792M reads (average of 2M reads per sample, range = 4K - 100M).

Bioinformatics Pipeline

The resulting 203 fastq files were first analyzed using the open source software FASTQC (9), to characterize raw sequence data quality. Fastq files were subsequently processed using an existing pipeline (CZID)(10), to filter low quality, low complexity, redundant and host sequences using Trimmomatic (11), PRICE (12), Lempel-Ziv-Welch (LZW) compression ratio, CD-HIT-DUP (13) and alignment to the human (Hg38) and chimpanzee (*Pan troglodytes*) using STAR (14) and Bowtie2 (15). Remaining reads were assigned to microbial taxa by querying the NCBI nucleotide

(NT) and nonredundant protein (NR) databases, with GSNAPL (16) and RAPSEARCH2 (17), respectively. Host reads were pseudoaligned to the human transcriptome using kallisto (18). A negative binomial model (NBM) was used to identify non-host reads that were present at significant levels over background signal detected in water and HeLa control samples, as described in Mick et al, 2020(19). Reads attributable to taxa with p values >0.1 in comparison to background were removed from further analysis; likewise reads attributable to taxa that had less than 10 hits by NT and 10 hits by NR, and those with alignment lengths of less than 70 bases were excluded from further analysis. A total of 65 samples were excluded for analysis if they contained >30% ERCC spike-in control RNA signaling low input (n=47), contained >50% ribosomal RNA reads signaling poor ribosomal depletion (n=1), were flagged as having poor per base sequence quality by the program FASTQC (n=6), or had no remaining microbial signal after removal of background non-host signal and low abundance taxa (<10 NT counts and <10 NR counts) (n = 11).

Anti-CMV and anti-EBV antibody Luminex assay:

Anti-CMV and anti-EBV IgG and IgM levels in participants' plasma were estimated using a beads-based Luminex immunoassay. Briefly, recombinant, His-tagged- human cytomegalovirus glycoprotein B and EBV glycoprotein 350 (Sino Biological, Houston, TX)- were individually coupled to barcoded MagPlex®-C Microsphere beads according to manufacturer's recommendations (Luminex Technology, Austin, TX). These antigen-coupled beads and Assay Chex control beads (Radix BioSolutions, Georgetown, TX) were added in each well of an assay plate. Participants' plasma samples were diluted 1:400 in PBS + 0.5% Triton X-100, and 25 μl of the diluted plasma was incubated with the antigen-coupled beads per well for 2 hours at RT on an orbital shaker. Following a wash step, the beads were incubated with a PE-conjugated

secondary antibody, viz., goat-anti Human IgG or goat anti-human IgM (Jackson ImmunoResearch Labs, West Grove, PA) for 30 minutes at RT on an orbital shaker. Following a wash, the assay plate was read on a Luminex Flex 3D instrument. Each output csv file was analyzed using MasterPlex QT Software (MiraiBio, Hitachi), and antibody estimates were reported as Mean Fluorescence Intensity (MFI) values. A raw MFI cut-off value of 1000 and 200 was empirically determined for high versus low anti-CMV IgG and anti-EBV IgM respectively based on the distribution of cumulative readouts for the entire sample set tested.

Supplemental Acknowledgements

the Stanford COVID-19 Biobank Study Group[‡] (Rebecca Osborne, Amrita Herkal, Tiffany Nguyen, Melissa Salazar, Rex Capulong, James V. Quinn, Jennifer A. Newberry, Nikhil Ram-Mohan, David Kim, Anita Visweswaran, Thanmayi Ranganath, Jonasel Roque, Komal Kumar, Kathryn Jee, Elizabeth Jordan Zudock, Rosen Mann, Brigit Noon, Jill Anderson, Bethany Fay, Donald Schreiber, Nancy Zhao, Rosemary Vergara, Julia McKechnie, Aaron Wilk, Lauren de la Parte, Kathleen Whittle Dantzler, Maureen Ty, Nimish Kathale, Arjun Rustagi, Giovanny Martinez-Colon, Geoff Ivison, Ruoxi Pi, Maddie Lee, Rachel Brewer, Taylor Hollis, Andrea Baird, Michele Ugur, Drina Bogusch, Georgie Nahass, Kazim Haider, Kim Quyen Thi Tran, Laura Simpson, Michal Tal, James Krempski, Shu-Chen Lyu) for their help in processing and storing samples, We would also like to thank the CLIAHUB Consortium †(Emily D. Crawford, Irene Acosta, Vida Ahyong, Erika C. Anderson, Shaun Arevalo, Daniel Asarnow, Shannon Axelrod, Patrick Ayscue, Camillia S. Azimi, Caleigh M. Azumaya, Stefanie Bachl, Iris Bachmutsky, Aparna Bhaduri, Jeremy Bancroft Brown, Joshua Batson, Astrid Behnert, Ryan M. Boileau, Saumya R. Bollam, Alain R. Bonny, David Booth, Michael Jerico B. Borja, David

Brown, Bryan Buie, Cassandra E. Burnett, Lauren E. Byrnes, Katelyn A. Cabral, Joana P. Cabrera, Saharai Caldera, Gabriela Canales, Gloria R. Castañeda, Agnes Protacio Chan, Christopher R. Chang, Arthur Charles-Orszag, Carly Cheung, Unseng Chio, Eric D. Chow, Y. Rose Citron, Allison Cohen, Lillian B. Cohn, Charles Chiu, Mitchel A. Cole, Daniel N. Conrad, Angela Constantino, Andrew Cote, Tre'Jon Crayton-Hall, Spyros Darmanis, Angela M. Detweiler, Rebekah L. Dial, Shen Dong, Elias M. Duarte, David Dynerman, Rebecca Egger, Alison Fanton, Stacey M. Frumm, Becky Xu Hua Fu, Valentina E. Garcia, Julie Garcia, Christina Gladkova, Miriam Goldman, Rafael Gomez-Sjoberg, M. Grace Gordon, James C. R. Grove, Shweta Gupta, Alexis Haddjeri-Hopkins, Pierce Hadley, John Haliburton, Samantha L. Hao, George Hartoularos, Nadia Herrera, Melissa Hilberg, Kit Ying E. Ho, Nicholas Hoppe, Shayan Hosseinzadeh, Conor J. Howard, Jeffrey A. Hussmann, Elizabeth Hwang, Danielle Ingebrigtsen, Julia R. Jackson, Ziad M. Jowhar, Danielle Kain, James Y. S. Kim, Amy Kistler, Oriana Kreutzfeld, Jessie Kulsuptrakul, Andrew F. Kung, Charles Langelier, Matthew T. Laurie, Lena Lee, Kun Leng, Kristoffer E. Leon, Manuel D. Leonetti, Sophia R. Levan, Sam Li, Aileen W. Li, Jamin Liu, Heidi S. Lubin, Amy Lyden, Jennifer Mann, Sabrina Mann, Gorica Margulis, Diana M. Marquez, Bryan P. Marsh, Calla Martyn, Elizabeth E. McCarthy, Aaron McGeever, Alexander F. Merriman, Lauren K. Meyer, Steve Miller, Megan K. Moore, Cody T. Mowery, Tanzila Mukhtar, Lusajo L. Mwakibete, Noelle Narez, Norma F. Neff, Lindsay A. Osso, Diter Oviedo, Suping Peng, Maira Phelps, Kiet Phong, Peter Picard, Lindsey M. Pieper, Neha Pincha, Angela Oliveira Pisco, Angela Pogson, Sergei Pourmal, Robert R. Puccinelli, Andreas S. Puschnik, Elze Rackaityte, Preethi Raghavan, Madhura Raghavan, James Reese, Joseph M. Replogle, Hanna Retallack, Helen Reyes, Donald Rose, Marci F. Rosenberg, Estella Sanchez-Guerrero, Sydney M. Sattler, Laura Savy, Stephanie K. See, Kristin K. Sellers, Paula Hayakawa

Serpa, Maureen Sheehy, Jonathan Sheu, Sukrit Silas, Jessica A. Streithorst, Jack Strickland, Doug Stryke, Sara Sunshine, Peter Suslow, Renaldo Sutanto, Serena Tamura, Michelle Tan, Jiongyi Tan, Alice Tang, Cristina M. Tato, Jack C. Taylor, Iliana Tenvooren, Erin M. Thompson, Edward C. Thornborrow, Eric Tse, Tony Tung, Marc L. Turner, Victoria S. Turner, Rigney E. Turnham, Mary J. Turocy, Trisha V. Vaidyanathan, Ilia D. Vainchtein, Manu Vanaerschot, Sara E. Vazquez, Anica M. Wandler, Anne Wapniarski, James T. Webber, Zara Y. Weinberg, Alexandra Westbrook, Allison W. Wong, Emily Wong, Gajus Worthington, Fang Xie, Albert Xu, Terrina Yamamoto, Ying Yang, Fauna Yarza, Yefim Zaltsman, Tina Zheng, Joseph L. DeRisi) for sample analysis,

REFERENCES

1. Health NIo. Clinical Spectrum of SARS-CoV-2 Infection. https://www.covid19treatmentguidelines.nih.gov/overview/clinical-spectrum/. Accessed August 1, 2021.
2. Bulterys PL, Garamani N, Stevens B, Sahoo MK, Huang C, Hogan CA, et al. Comparison of a laboratory-developed test targeting the envelope gene with three nucleic acid amplification tests for detection of SARS-CoV-2. *J Clin Virol.* 2020;129:104427.
3. U.S. Food and Drug Administration. Stanford Health Care Clinical Virology Laboratory SARS-CoV-2 test EUA Summary. https://www.fda.gov/media/136818/download. Accessed April 10 2020.
4. U.S. Food and Drug Administration. Xpert® Xpress SARS-CoV-2. https://www.fda.gov/media/136314/download. Accessed May 27 2020.
5. U.S. Food and Drug Administration. Panther Fusion SARS-CoV-2 Emergency Use Authorization. https://www.fda.gov/media/136153/download. Accessed April 4 2020.
6. Hogan CA, Sahoo MK, Huang C, Garamani N, Stevens B, Zehnder J, et al. Comparison of the Panther Fusion and a laboratory-developed test targeting the envelope gene for detection of SARS-CoV-2. *J Clin Virol.* 2020;127:104383.
7. Crawford ED, Acosta I, Ahyong V, Anderson EC, Arevalo S, Asarnow D, et al. Rapid deployment of SARS-CoV-2 testing: The CLIAHUB. *PLoS Pathog.* 2020;16(10):e1008966.
8. Mayday MY, Khan LM, Chow ED, Zinter MS, and DeRisi JL. Miniaturization and optimization of 384-well compatible RNA sequencing library preparation. *PLoS One.* 2019;14(1):e0206194.

9. Andrews S. FastQC: a quality control tool for high throughput sequence data. http://www.bioinformatics.babraham.ac.uk/projects/fastqc. Accessed March 3, 2022.

10. Kalantar KL, Carvalho T, de Bourcy CFA, Dimitrov B, Dingle G, Egger R, et al. IDseq-An open source cloud-based pipeline and analysis service for metagenomic pathogen detection and monitoring. *Gigascience.* 2020;9(10).

11. Bolger AM, Lohse M, and Usadel B. Trimmomatic: a flexible trimmer for Illumina sequence data. *Bioinformatics.* 2014;30(15):2114-20.

12. Ruby JG, Bellare P, and Derisi JL. PRICE: software for the targeted assembly of components of (Meta) genomic sequence data. *G3 (Bethesda).* 2013;3(5):865-80.

13. Li W, and Godzik A. Cd-hit: a fast program for clustering and comparing large sets of protein or nucleotide sequences. *Bioinformatics.* 2006;22(13):1658-9.

14. Dobin A, Davis CA, Schlesinger F, Drenkow J, Zaleski C, Jha S, et al. STAR: ultrafast universal RNA-seq aligner. *Bioinformatics.* 2013;29(1):15-21.

15. Langmead B, and Salzberg SL. Fast gapped-read alignment with Bowtie 2. *Nat Methods.* 2012;9(4):357-9.

16. Wu TD, and Nacu S. Fast and SNP-tolerant detection of complex variants and splicing in short reads. *Bioinformatics.* 2010;26(7):873-81.

17. Zhao Y, Tang H, and Ye Y. RAPSearch2: a fast and memory-efficient protein similarity search tool for next-generation sequencing data. *Bioinformatics.* 2012;28(1):125-6.

18. Bray NL, Pimentel H, Melsted P, and Pachter L. Near-optimal probabilistic RNA-seq quantification. *Nat Biotechnol.* 2016;34(5):525-7.

19. Mick E, Kamm J, Pisco AO, Ratnasiri K, Babik JM, Castañeda G, et al. Upper airway gene expression reveals suppressed immune responses to SARS-CoV-2 compared with other respiratory viruses. *Nat Commun.* 2020;11(1):5854.
