# Supplementary material for: Anti-nucleocapsid antibody levels and pulmonary comorbid conditions are linked to post–COVID-19 syndrome
Source: JCI Insight. 2022 Jul 8;7(13):e156713. doi: 10.1172/jci.insight.156713 (PMC9310538; doi:10.1172/jci.insight.156713)
Supplement: Supplemental table 4 [file jciinsight-7-156713-s083.pdf]

```
COVIDqPCR,,,,,,,,,
,Record.ID,Initial_COVID_diagnosis,Initial_COVID_symptoms,Nasal.swab_I
D,Nasal.swab__COVID_call,target_genes_above_background,target_genes_ra
w_Ct,CZB_lib_IDS,control
1,55689-0001,positive,mild,55689-0001 Month 2
(EC001),negative,RNaseP,35.81,RR067e_00116,0
2,55689-0001,positive,mild,55689-0001
V7,negative,RNaseP,35.61,RR067e_00635,0
3,55689-0001,positive,mild,55689-0001-
Month3,negative,RNaseP,34.57,RR067e_00168,0
4,55689-0003,positive,moderate,55689-0003 Month
2,negative,RNaseP,31.11,RR067e_00121,0
5,55689-0003,positive,moderate,55689-0003
V6,negative,RNaseP,27.49,RR067e_00606,0
6,55689-0004,positive,mild,55689-0004 Month
2,negative,RNaseP,31.66,RR067e_00112,0
7,55689-0004,positive,mild,55689-0004-
Month3,negative,RNaseP,29.04,RR067e_00169,0
8,55689-0004,positive,mild,55689-0004-
V8,negative,RNaseP,28.67,RR067e_00743,0
9,55689-0005,positive,mild,55689-0005-
Month3,negative,RNaseP,32.24,RR067e_00176,0
10,55689-0005,positive,mild,55689-0005-
V8,negative,RNaseP,26.95,RR067e_00744,0
11,55689-0006,positive,moderate,55689-0006 Month
2,negative,RNaseP,32.26,RR067e_00108,0
12,55689-0007,positive,mild,55689-0007 Month
2,negative,RNaseP,33.89,RR067e_00123,0
13,55689-0007,positive,mild,55689-0007
V6,negative,RNaseP,28.18,RR067e_00604,0
14,55689-0007,positive,mild,55689-0007-
V9,negative,RNaseP,27.9,RR067e_00731,0
15,55689-0008,positive,mild,55689-0008 Month
2,negative,RNaseP,25.07,RR067e_00124,0
16,55689-0008,positive,mild,55689-0008
V7,negative,RNaseP,26.9,RR067e_00627,0
17,55689-0009,positive,severe,55689-0009 Month
2,negative,RNaseP,29.84,RR067e_00122,0
18,55689-0009,positive,severe,55689-0009
V7,negative,RNaseP,24.8,RR067e_00628,0
19,55689-0010,positive,mild,55689-0010
V6,negative,RNaseP,26.7,RR067e_00603,0
20,55689-0010,positive,mild,55689-0010-
Month2,negative,RNaseP,28.67,RR067e_00194,0
21,55689-0011,positive,mild,55689-0011 Month
2,negative,RNaseP,30.45,RR067e_00113,0
22,55689-0011,positive,mild,55689-0011
V6,negative,RNaseP,28.32,RR067e_00607,0
23,55689-0011,positive,mild,55689-0011-
V9,negative,RNaseP,25.88,RR067e_00719,0
```

24,55689-0012,positive,mild,55689-0012 Month
2,negative,RNaseP,31.22,RR067e_00118,0
25,55689-0012,positive,mild,55689-0012
V6,negative,RNaseP,26.31,RR067e_00605,0
26,55689-0013,positive,moderate,55689-0013 Month
2,negative,RNaseP,30.36,RR067e_00114,0
27,55689-0013,positive,moderate,55689-0013-
Month3,negative,RNaseP,30.87,RR067e_00171,0
28,55689-0013,positive,moderate,55689-0013-
V8,negative,RNaseP,29.14,RR067e_00709,0
29,55689-0013,positive,moderate,55689-0013-
V9,negative,RNaseP,25.88,RR067e_00723,0
30,55689-0015,positive,moderate,55689-0015-
D30,negative,RNaseP,34.85,RR067e_00015,0
31,55689-0017,positive,mild,55689-0017
V8,negative,RNaseP,27.35,RR067e_00653,0
32,55689-0017,positive,mild,55689-0017-
Month3,negative,RNaseP,28.64,RR067e_00179,0
33,55689-0017,positive,mild,55689-0017-
Month4,negative,RNaseP,25.84,RR067e_00198,0
34,55689-0017,positive,mild,55689-0017-
V9,negative,RNaseP,27.35,RR067e_00695,0
35,55689-0018,positive,mild,55689-0018-
Month4,negative,RNaseP,30.75,RR067e_00196,0
36,55689-0019,positive,mild,55689-0019 Month
2,negative,RNaseP,32.8,RR067e_00131,0
37,55689-0022,positive,mild,55689-0022 Month
2,negative,RNaseP,26.67,RR067e_00138,0
38,55689-0022,positive,mild,55689-0022
V6,negative,RNaseP,27.24,RR067e_00614,0
39,55689-0022,positive,mild,55689-0022-
D0,negative,RNaseP,32.48,RR067e_00016,0
40,55689-0023,positive,mild,55689-0023 Month
2,negative,RNaseP,28.34,RR067e_00139,0
41,55689-0023,positive,mild,55689-0023-
D0,negative,RNaseP,29.33,RR067e_00025,0
42,55689-0024,positive,mild,55689-0024 Month
2,negative,RNaseP,27.88,RR067e_00140,0
43,55689-0024,positive,mild,55689-0024
V6,negative,RNaseP,25.22,RR067e_00617,0
44,55689-0024,positive,mild,55689-0024-
D30,positive,"RNaseP,E","30.06,36.81",RR067e_00049,0
45,55689-0024,positive,mild,55689-0024-
Month3,negative,RNaseP,30.11,RR067e_00184,0
46,55689-0024,positive,mild,55689-0024-
Month4,positive,"RNaseP,E","23.93,37.1",RR067e_00203,0
47,55689-0024,positive,mild,55689-0024-
V8,negative,RNaseP,26.07,RR067e_00699,0
48,55689-0026,positive,mild,55689-0026 Month
2,negative,RNaseP,28.61,RR067e_00132,0

```
49,55689-0026,positive,mild,55689-0026
V7,negative,RNaseP,27.94,RR067e_00641,0
50,55689-0026,positive,mild,55689-0026-
D0,negative,RNaseP,32.82,RR067e_00034,0
51,55689-0026,positive,mild,55689-0026-
D30,negative,RNaseP,31.36,RR067e_00066,0
52,55689-0026,positive,mild,55689-0026-
Month3,negative,RNaseP,31.14,RR067e_00183,0
53,55689-0027,positive,mild,55689-0027-
D30,negative,RNaseP,32.75,RR067e_00023,0
54,55689-0028,positive,mild,55689-0028-
D0,negative,RNaseP,32.66,RR067e_00043,0
55,55689-0029,positive,mild,55689-0029
V7,negative,RNaseP,28.66,RR067e_00654,0
56,55689-0029,positive,mild,55689-0029-
D0,negative,RNaseP,31.35,RR067e_00052,0
57,55689-0029,positive,mild,55689-0029-
Month2,negative,RNaseP,30.67,RR067e_00185,0
58,55689-0030,positive,mild,55689-0030-
D0,negative,RNaseP,31.08,RR067e_00001,0
59,55689-0031,positive,mild,55689-0031 Month
2,negative,RNaseP,29.66,RR067e_00144,0
60,55689-0031,positive,mild,55689-0031-
D0,negative,RNaseP,29.39,RR067e_00035,0
61,55689-0031,positive,mild,55689-0031-
D30,negative,RNaseP,34.16,RR067e_00006,0
62,55689-0032,positive,mild,55689-0032 Month
2,negative,RNaseP,33.18,RR067e_00134,0
63,55689-0032,positive,mild,55689-0032
V6,negative,RNaseP,26.25,RR067e_00609,0
64,55689-0032,positive,mild,55689-0032-
D0,negative,RNaseP,34.3,RR067e_00008,0
65,55689-0033,positive,mild,55689-0033 Month
2,negative,RNaseP,29.79,RR067e_00137,0
66,55689-0033,positive,mild,55689-0033
V6,negative,RNaseP,27.81,RR067e_00745,0
67,55689-0033,positive,mild,55689-0033-
D0,negative,RNaseP,30.13,RR067e_00026,0
68,55689-0033,positive,mild,55689-0033-
Month4,negative,RNaseP,27.99,RR067e_00195,0
69,55689-0034,positive,mild,55689-0034 Month
2,negative,RNaseP,36.64,RR067e_00135,0
70,55689-0034,positive,mild,55689-0034
V6,negative,RNaseP,32.36,RR067e_00746,0
71,55689-0034,positive,mild,55689-0034-
D0,negative,RNaseP,30.61,RR067e_00044,0
72,55689-0035,positive,mild,55689-0035
V7,negative,RNaseP,27.38,RR067e_00650,0
73,55689-0035,positive,mild,55689-0035-
D0,negative,RNaseP,25.71,RR067e_00053,0
```

74,55689-0035,positive,mild,55689-0035-
D30,negative,RNaseP,29.32,RR067e_00021,0
75,55689-0035,positive,mild,55689-0035-
Month3,negative,RNaseP,27.5,RR067e_00193,0
76,55689-0036,positive,mild,55689-0036-
D0,negative,RNaseP,31.01,RR067e_00060,0
77,55689-0036,positive,mild,55689-0036-
D30,negative,RNaseP,35.79,RR067e_00014,0
78,55689-0037,positive,mild,55689-0037
V5,negative,RNaseP,25.78,RR067e_00601,0
79,55689-0037,positive,mild,55689-0037-
D0,negative,RNaseP,28.15,RR067e_00009,0
80,55689-0037,positive,mild,55689-0037-
D30,negative,RNaseP,33.41,RR067e_00063,0
81,55689-0037,positive,mild,55689-0037-
V8,negative,RNaseP,27.55,RR067e_00740,0
82,55689-0038,positive,mild,55689-0038-
D0,negative,RNaseP,29.14,RR067e_00054,0
83,55689-0039,positive,mild,55689-0039-
D0,negative,RNaseP,30.43,RR067e_00017,0
84,55689-0039,positive,mild,55689-0039-
D30,negative,RNaseP,33.94,RR067e_00074,0
85,55689-0039,positive,mild,55689-0039-
Month2,negative,RNaseP,29.06,RR067e_00189,0
86,55689-0040,positive,mild,55689-0040
V5,negative,RNaseP,25.52,RR067e_00588,0
87,55689-0040,positive,mild,55689-0040
V7,negative,RNaseP,26.41,RR067e_00640,0
88,55689-0040,positive,mild,55689-0040-
D0,negative,RNaseP,28.14,RR067e_00018,0
89,55689-0040,positive,mild,55689-0040-
D30,negative,RNaseP,29.21,RR067e_00005,0
90,55689-0040,positive,mild,55689-0040-
Month4,negative,RNaseP,24.8,RR067e_00207,0
91,55689-0041,positive,moderate,55689-0041-
D0,negative,RNaseP,31.17,RR067e_00027,0
92,55689-0042,positive,mild,55689-0042-
D0,negative,RNaseP,31.16,RR067e_00036,0
93,55689-0042,positive,mild,55689-0042-
D30,negative,RNaseP,30.46,RR067e_00073,0
94,55689-0042,positive,mild,55689-0042
V7,Invalid,None,NA,RR067e_00597,0
95,55689-0042,positive,mild,55689-0042-
V8,negative,RNaseP,26.41,RR067e_00672,0
96,55689-0043,positive,mild,55689-0043
V5,negative,RNaseP,28.75,RR067e_00590,0
97,55689-0043,positive,mild,55689-0043-
D0,negative,RNaseP,32.1,RR067e_00045,0
98,55689-0043,positive,mild,55689-0043-
D30,negative,RNaseP,32.17,RR067e_00078,0

99,55689-0043,positive,mild,55689-0043-
Month4,negative,RNaseP,28.11,RR067e_00209,0
100,55689-0044,positive,mild,55689-0044
V7,negative,RNaseP,25.9,RR067e_00656,0
101,55689-0044,positive,mild,55689-0044-
D0,negative,RNaseP,26.81,RR067e_00002,0
102,55689-0044,positive,mild,55689-0044-
D30,negative,RNaseP,29.97,RR067e_00075,0
103,55689-0044,positive,mild,55689-0044-
Month3,negative,RNaseP,29.01,RR067e_00187,0
104,55689-0045,positive,mild,55689-0045
V5,negative,RNaseP,33.58,RR067e_00587,0
105,55689-0045,positive,mild,55689-0045-
D0,positive,"RNaseP,E","32.06,36.75",RR067e_00061,0
106,55689-0045,positive,mild,55689-0045-
D30,negative,RNaseP,37.36,RR067e_00070,0
107,55689-0045,positive,mild,55689-0045-
Month4,negative,RNaseP,33.09,RR067e_00206,0
108,55689-0046,positive,mild,55689-0046
V7,negative,RNaseP,27.6,RR067e_00662,0
109,55689-0046,positive,mild,55689-0046-
D0,negative,RNaseP,26.66,RR067e_00003,0
110,55689-0046,positive,mild,55689-0046-
Month3,negative,RNaseP,26,RR067e_00192,0
111,55689-0047,positive,mild,55689-0047
V5,negative,RNaseP,28.26,RR067e_00591,0
112,55689-0047,positive,mild,55689-0047-
D0,negative,RNaseP,29.09,RR067e_00010,0
113,55689-0047,positive,mild,55689-0047-
D30,negative,RNaseP,31.11,RR067e_00067,0
114,55689-0047,positive,mild,55689-0047-
Month4,negative,RNaseP,27.84,RR067e_00210,0
115,55689-0047,positive,mild,55689-0047-
V8,negative,RNaseP,27.71,RR067e_00705,0
116,55689-0049,positive,moderate,55689-0049
V6,negative,RNaseP,27.01,RR067e_00620,0
117,55689-0050,positive,mild,55689-0050
V7,negative,RNaseP,29.05,RR067e_00648,0
118,55689-0050,positive,mild,55689-0050-
D30,negative,RNaseP,32.44,RR067e_00080,0
119,55689-0050,positive,mild,55689-0050-
Month2,negative,RNaseP,34.1,RR067e_00177,0
120,55689-0051,positive,mild,55689-0051
V7,negative,RNaseP,28.95,RR067e_00634,0
121,55689-0051,positive,mild,55689-0051-
D0,negative,RNaseP,34.07,RR067e_00028,0
122,55689-0051,positive,mild,55689-0051-
D30,negative,RNaseP,29.88,RR067e_00081,0
123,55689-0051,positive,mild,55689-0051-
Month3,negative,RNaseP,25.59,RR067e_00204,0

124,55689-0051,positive,mild,55689-0051-
V8,negative,RNaseP,26.29,RR067e_00729,0
125,55689-0052,positive,mild,55689-0052-
D0,negative,RNaseP,30.92,RR067e_00037,0
126,55689-0052,positive,mild,55689-0052-
D30,negative,RNaseP,33.11,RR067e_00071,0
127,55689-0053,positive,mild,55689-0053-
D0,negative,RNaseP,34.29,RR067e_00046,0
128,55689-0053,positive,mild,55689-0053-
D30,negative,RNaseP,36.59,RR067e_00069,0
129,55689-0054,positive,mild,55689-0054 Month
2,negative,RNaseP,35.34,RR067e_00141,0
130,55689-0054,positive,mild,55689-0054-
D0,negative,RNaseP,30.7,RR067e_00055,0
131,55689-0056,positive,mild,55689-0056 Month
2,negative,RNaseP,31.48,RR067e_00147,0
132,55689-0056,positive,mild,55689-0056
V6,negative,RNaseP,28.54,RR067e_00624,0
133,55689-0056,positive,mild,55689-0056
V7,negative,RNaseP,30.01,RR067e_00655,0
134,55689-0057,positive,moderate,55689-0057
V5,negative,RNaseP,32.11,RR067e_00592,0
135,55689-0057,positive,moderate,55689-0057-
D0,negative,RNaseP,28.47,RR067e_00062,0
136,55689-0057,positive,moderate,55689-0057-
D30,negative,RNaseP,35.04,RR067e_00084,0
137,55689-0057,positive,moderate,55689-0057-
V8,negative,RNaseP,30.23,RR067e_00694,0
138,55689-0058,positive,moderate,55689-0058 Month
2,negative,RNaseP,28.73,RR067e_00152,0
139,55689-0058,positive,moderate,55689-0058
V7,negative,RNaseP,26.83,RR067e_00660,0
140,55689-0058,positive,moderate,55689-0058-
D0,negative,RNaseP,31.19,RR067e_00029,0
141,55689-0058,positive,moderate,55689-0058-
D30,negative,RNaseP,27.94,RR067e_00085,0
142,55689-0059,positive,mild,55689-0059-
D0,negative,RNaseP,29.69,RR067e_00038,0
143,55689-0060,positive,mild,55689-0060-
D0,negative,RNaseP,31.8,RR067e_00004,0
144,55689-0060,negative,NA,55689-0060-
D30,negative,RNaseP,30.46,RR067e_00120,0
145,55689-0062,negative,NA,55689-0062
V5,negative,RNaseP,24.47,RR067e_00589,1
146,55689-0062,negative,NA,55689-0062-
D0,negative,RNaseP,27.77,RR067e_00020,1
147,55689-0062,negative,NA,55689-0062-
D30,negative,RNaseP,27.71,RR067e_00077,1
148,55689-0062,negative,NA,55689-0062-
Month4,negative,RNaseP,24.13,RR067e_00208,1

149,55689-0063,negative,NA,55689-0063-
D0,negative,RNaseP,31.18,RR067e_00047,1
150,55689-0063,negative,NA,55689-0063-
D30,negative,RNaseP,31.64,RR067e_00089,1
151,55689-0065,positive,moderate,55689-0065-
D0,positive,"RNaseP,E,N","31,34.8,37.03",RR067e_00030,0
152,55689-0065,positive,moderate,55689-0065-
D30,negative,RNaseP,28.41,RR067e_00106,0
153,55689-0066,positive,mild,55689-0066-
D0,negative,RNaseP,29.94,RR067e_00048,0
154,55689-0066,positive,mild,55689-0066-
D30,negative,RNaseP,31.16,RR067e_00087,0
155,55689-0067,positive,mild,55689-0067-
D0,negative,RNaseP,29.89,RR067e_00041,0
156,55689-0067,positive,mild,55689-0067-
D30,negative,RNaseP,29,RR067e_00088,0
157,55689-0072,positive,mild,55689-0072
V7,negative,RNaseP,27.22,RR067e_00599,0
158,55689-0072,positive,mild,55689-0072-
D0,negative,RNaseP,32.08,RR067e_00057,0
159,55689-0072,positive,mild,55689-0072-
D30,negative,RNaseP,31.9,RR067e_00091,0
160,55689-0072,positive,mild,55689-0072-
V8,negative,RNaseP,27.18,RR067e_00687,0
161,55689-0073,positive,mild,55689-0073
V6,negative,RNaseP,27.77,RR067e_00626,0
162,55689-0073,positive,mild,55689-0073
V7,negative,RNaseP,27.34,RR067e_00636,0
163,55689-0073,positive,mild,55689-0073-
D0,negative,RNaseP,30.82,RR067e_00064,0
164,55689-0073,positive,mild,55689-0073-
D30,negative,RNaseP,28.97,RR067e_00092,0
165,55689-0073,positive,mild,55689-0073-
Month2,negative,RNaseP,31.07,RR067e_00178,0
166,55689-0074,positive,mild,55689-0074
V6,negative,RNaseP,27.47,RR067e_00631,0
167,55689-0074,positive,mild,55689-0074-
D0,negative,RNaseP,29.75,RR067e_00059,0
168,55689-0074,positive,mild,55689-0074-
D30,negative,RNaseP,29.38,RR067e_00093,0
169,55689-0074,positive,mild,55689-0074-
Month3,negative,RNaseP,30.48,RR067e_00170,0
170,55689-0076,positive,mild,55689-0076
V7,negative,RNaseP,29.23,RR067e_00659,0
171,55689-0076,positive,mild,55689-0076-
D30,negative,RNaseP,30.37,RR067e_00082,0
172,55689-0076,positive,mild,55689-0076-
Month2,negative,RNaseP,33.51,RR067e_00174,0
173,55689-0081,positive,mild,55689-0081
V6,negative,RNaseP,27.87,RR067e_00595,0

174,55689-0081,positive,mild,55689-0081-
D30,negative,RNaseP,32.24,RR067e_00083,0
175,55689-0081,positive,mild,55689-0081-
V7,positive,"E,RNaseP","35.7,28.09",RR067e_00670,0
176,55689-0082,positive,mild,55689-0082
V6,negative,RNaseP,29.65,RR067e_00625,0
177,55689-0082,positive,mild,55689-0082-
D30,negative,RNaseP,30.31,RR067e_00129,0
178,55689-0082,positive,mild,55689-0082-
Month2,negative,RNaseP,30.32,RR067e_00167,0
179,55689-0083,positive,mild,55689-0083
V5,negative,RNaseP,32.14,RR067e_00623,0
180,55689-0083,positive,mild,55689-0083-
D30,negative,RNaseP,32.9,RR067e_00119,0
181,55689-0085,positive,severe,55689-0085
V5,negative,RNaseP,28.12,RR067e_00612,0
182,55689-0086,positive,mild,55689-0086-
D30,negative,RNaseP,32.65,RR067e_00117,0
183,55689-0086,positive,mild,55689-0086-
Month2,negative,RNaseP,31.72,RR067e_00181,0
184,55689-0087,positive,moderate,55689-0087-
D30,negative,RNaseP,35.42,RR067e_00145,0
185,55689-0087,positive,moderate,55689-0087-
Month2,negative,RNaseP,35.64,RR067e_00180,0
186,55689-0090,positive,asymptomatic,55689-0090-
D30,negative,RNaseP,30.27,RR067e_00125,0
187,55689-0091,positive,asymptomatic,55689-0091
V6,negative,RNaseP,26.26,RR067e_00637,0
188,55689-0093,positive,mild,55689-0093
V5,negative,RNaseP,29.68,RR067e_00608,0
189,55689-0093,positive,mild,55689-0093-
D0,negative,RNaseP,32.36,RR067e_00056,0
190,55689-0093,positive,mild,55689-0093-
D30,negative,RNaseP,31.68,RR067e_00142,0
191,55689-0093,positive,mild,55689-0093-
Month2,negative,RNaseP,27.47,RR067e_00190,0
192,55689-0093,positive,mild,55689-0093-
Month3,negative,RNaseP,28.55,RR067e_00201,0
193,55689-0093,positive,mild,55689-0093-
V8,negative,RNaseP,29.98,RR067e_00724,0
194,55689-0094,positive,mild,55689-0094-
D0,negative,RNaseP,31.65,RR067e_00024,0
195,55689-0094,positive,mild,55689-0094-
Month3,negative,RNaseP,30.23,RR067e_00205,0
196,55689-0095,positive,mild,55689-0095-
Month2,negative,RNaseP,25.04,RR067e_00186,0
197,55689-0099,positive,moderate,55689-0099
V5,negative,RNaseP,28.19,RR067e_00616,0
198,55689-0099,positive,moderate,55689-0099-
D7,negative,RNaseP,33.21,RR067e_00068,0

199,55689-0100,positive,moderate,55689-0100-
D0,negative,RNaseP,31.53,RR067e_00076,0
200,55689-0101,positive,critical,55689-0101
V5,negative,RNaseP,26.98,RR067e_00618,0
201,55689-0102,positive,mild,55689-0102-
D0,negative,RNaseP,31.1,RR067e_00079,0
202,55689-0102,positive,mild,55689-0102-
D30,negative,RNaseP,33.36,RR067e_00172,0
203,55689-0103,positive,mild,55689-0103
V6,negative,RNaseP,28.1,RR067e_00596,0
204,55689-0104,positive,severe,55689-0104-
D0,negative,RNaseP,32.04,RR067e_00086,0
205,55689-0104,positive,severe,55689-0104-
D30,negative,RNaseP,32.18,RR067e_00133,0
206,55689-0104,positive,severe,55689-0104-
V6,negative,RNaseP,32.82,RR067e_00739,0
207,55689-0105,positive,critical,55689-0105
V6,negative,RNaseP,29.03,RR067e_00649,0
208,55689-0105,positive,critical,55689-0105-
D30,negative,RNaseP,35.05,RR067e_00109,0
209,55689-0106,positive,moderate,55689-0106-
D7,negative,RNaseP,31.47,RR067e_00094,0
210,55689-0114,positive,mild,55689-0114
V5,negative,RNaseP,30.34,RR067e_00642,0
211,55689-0114,positive,mild,55689-0114-
D0,negative,RNaseP,31.18,RR067e_00111,0
212,55689-0115,positive,moderate,55689-0115--
Month2,negative,RNaseP,24.08,RR067e_00202,0
213,55689-0115,positive,moderate,55689-0115-
D0,positive,"N,E,RNaseP","35.91,36.17,29.17",RR067e_00126,0
214,55689-0118,positive,critical,55689-0118
V4,negative,RNaseP,27.58,RR067e_00615,0
215,55689-0118,positive,critical,55689-0118-
D0,negative,RNaseP,32.83,RR067e_00130,0
216,55689-0120,positive,NA,55689-0120
V4,negative,RNaseP,30.26,RR067e_00622,1
217,55689-0120,positive,NA,55689-0120-
D0,negative,RNaseP,31.9,RR067e_00148,1
218,55689-0122,positive,severe,55689-0122-
Month4,negative,RNaseP,33.11,RR067e_00730,0
219,55689-0126,negative,NA,55689-0126
V3,negative,RNaseP,35.12,RR067e_00610,1
220,55689-0126,negative,NA,55689-0126-
D0,negative,RNaseP,34.79,RR067e_00191,1
221,55689-0126,negative,NA,55689-0126-
V6,negative,RNaseP,34.28,RR067e_00692,1
222,55689-0132,positive,mild,55689-0132
V4,negative,RNaseP,31.02,RR067e_00658,0
223,55689-0132,positive,mild,55689-0132-
D0,negative,RNaseP,28.4,RR067e_00197,0

224,55689-0138,positive,severe,55689-0138
V3,negative,RNaseP,29.09,RR067e_00638,0
225,55689-0141,positive,moderate,55689-0141
V3,negative,RNaseP,28.87,RR067e_00643,0
226,55689-0150,negative,NA,55689-0150
V1,negative,RNaseP,26.78,RR067e_00593,1
227,55689-0151,negative,NA,55689-0151
V1,negative,RNaseP,27.47,RR067e_00594,1
228,55689-0155,positive,critical,55689-0155
V2,negative,RNaseP,32.84,RR067e_00619,0
229,55689-0161,positive,critical,55689-0161
V2,negative,RNaseP,30.08,RR067e_00629,0
230,55689-0172,positive,critical,55689-0172-
D7,negative,RNaseP,30.81,RR067e_00710,0
231,55689-0173,positive,mild,55689-0173
V1,negative,RNaseP,31.52,RR067e_00630,0
232,55689-0177,positive,mild,55689-0177-
Month2,negative,RNaseP,27.22,RR067e_00706,0
233,55689-0180,positive,moderate,55689-0180
V1,negative,RNaseP,28.13,RR067e_00645,0
234,55689-0181,positive,mild,55689-0181
V1,negative,RNaseP,28.27,RR067e_00647,0
235,55689-0181,positive,mild,55689-0181-
D30,negative,RNaseP,26.31,RR067e_00685,0
236,55689-0181,positive,mild,55689-0181-
Month2,negative,RNaseP,27.54,RR067e_00691,0
237,55689-0182,positive,mild,55689-0182
V1,negative,RNaseP,27.61,RR067e_00652,0
238,55689-0183,positive,asymptomatic,55689-0183
V1,negative,RNaseP,27.69,RR067e_00651,0
239,55689-0184,positive,mild,55689-0184
V1,negative,RNaseP,30.26,RR067e_00644,0
240,55689-0185,positive,mild,55689-0185
V1,negative,RNaseP,27.6,RR067e_00646,0
241,55689-0185,positive,mild,55689-0185-
Month3,positive,"RNaseP,E,N","25.84,31.58,33.6",RR067e_00678,0
242,55689-0187,positive,mild,55689-0187
V1,negative,RNaseP,26.55,RR067e_00663,0
243,55689-0189,positive,mild,55689-0189
V1,negative,RNaseP,31.9,RR067e_00665,0
244,55689-0190,positive,mild,55689-0190
V1,negative,RNaseP,28.37,RR067e_00666,0
245,55689-0191,positive,mild,55689-0191
V1,negative,RNaseP,27.73,RR067e_00664,0
246,55689-0192,positive,mild,55689-0192-
D0,negative,RNaseP,27.07,RR067e_00737,0
247,55689-0196,positive,mild,55689-0196-
D0,negative,RNaseP,29.1,RR067e_00674,0
248,55689-0198,positive,mild,55689-0198-
D0,negative,RNaseP,33.01,RR067e_00736,0

```
249,55689-0200,positive,mild,55689-0200-
D0,negative,RNaseP,29,RR067e_00677,0
250,55689-0201,positive,mild,55689-0201-
D0,negative,RNaseP,30.28,RR067e_00732,0
251,55689-0203,positive,mild,55689-0203-
D0,negative,RNaseP,26,RR067e_00698,0
252,55689-0205,positive,mild,55689-0205-
D0,negative,RNaseP,24.83,RR067e_00708,0
253,55689-0207,positive,mild,55689-0207-
D0,negative,RNaseP,27.55,RR067e_00688,0
254,55689-0208,negative,NA,55689-0208-
D0,negative,RNaseP,28.68,RR067e_00676,1
255,55689-0209,positive,mild,55689-0209-
D0,negative,RNaseP,31.27,RR067e_00716,0
256,55689-0210,positive,mild,55689-0210-
D0,negative,RNaseP,27.29,RR067e_00689,0
257,55689-0211,positive,mild,55689-0211-
D0,negative,RNaseP,26.5,RR067e_00718,0
258,55689-0212,positive,mild,55689-0212-
D0,positive,"E,RNaseP","37.31,28.05",RR067e_00684,0
259,55689-0213,positive,mild,55689-0213-
D0,negative,RNaseP,27.4,RR067e_00683,0
260,55689-0215,positive,critical,55689-0215-
D0,negative,RNaseP,28.14,RR067e_00727,0
261,55689-0217,positive,mild,55689-0217-
D0,positive,"RNaseP,N","25.18,44.15",RR067e_00693,0
262,55689-0218,positive,mild,55689-0218-
D0,positive,"RNaseP,N,E","29.35,37.23,36.28",RR067e_00726,0
263,55689-0219,positive,mild,55689-0219-
D0,positive,"E,RNaseP","37.21,25.85",RR067e_00682,0
264,55689-0220,positive,moderate,55689-0220-
D0,negative,RNaseP,27.52,RR067e_00703,0
265,55689-0221,positive,asymptomatic,55689-0221-
D0,negative,RNaseP,26.17,RR067e_00712,0
266,55689-0222,positive,mild,55689-0222-
D0,negative,RNaseP,25.45,RR067e_00717,0
267,55689-0222,positive,mild,55689-0222-
Month2,positive,"E,RNaseP","37.21,26.02",RR067e_00679,0
268,55689-0223,positive,mild,55689-0223-
D0,negative,RNaseP,25.93,RR067e_00714,0
269,55689-0224,positive,mild,55689-0224-
D0,negative,RNaseP,24.39,RR067e_00725,0
270,55689-0225,positive,mild,55689-0225-
D0,negative,RNaseP,27.33,RR067e_00681,0
271,55689-0226,positive,mild,55689-0226-
D0,negative,RNaseP,27.15,RR067e_00713,0
272,55689-0231,positive,mild,55689-0231-
D0,negative,RNaseP,29.79,RR067e_00690,0
273,55689-0232,positive,mild,55689-0232-
D0,negative,RNaseP,30.32,RR067e_00667,0
```

274,55689-0235,positive,mild,55689-0235-
D0,negative,RNaseP,29.41,RR067e_00701,0
275,55689-0247,positive,mild,55689-0247-
D0,positive,"E,N,RNaseP","37.35,35.06,26.23",RR067e_00707,0
276,OHC-010,?,?,58356-0008-
Baseline,negative,RNaseP,33.37,RR067e_00738,0
277,55689-0199,?,?,58356-0016-
Baseline,negative,RNaseP,26.68,RR067e_00700,0
278,55689-0120,?,?,FLU-58356-0012-
Baseline,negative,RNaseP,32.76,RR067e_00734,1
279,OHC-001,positive,mild,OHC-001-
V02,negative,RNaseP,29.5,RR067e_00031,0
280,OHC-001,positive,mild,OHC-001-
V03,negative,RNaseP,28.59,RR067e_00095,0
281,OHC-001,positive,mild,OHC-001-
V04,negative,RNaseP,29.84,RR067e_00164,0
282,OHC-001,positive,mild,OHC-001-
V05,negative,RNaseP,27.66,RR067e_00759,0
283,OHC-003,positive,mild,OHC-003-
V02,negative,RNaseP,30.97,RR067e_00013,0
284,OHC-003,positive,mild,OHC-003-
V03,negative,RNaseP,31.16,RR067e_00100,0
285,OHC-003,positive,mild,OHC-003-
V04,negative,RNaseP,31.55,RR067e_00163,0
286,OHC-003,positive,mild,OHC-003-
V05,negative,RNaseP,31.64,RR067e_00214,0
287,OHC-003,positive,mild,OHC-003-
V06,negative,RNaseP,28.02,RR067e_00749,0
288,OHC-003,positive,mild,OHC-003-
V08,negative,RNaseP,29.36,RR067e_00765,0
289,OHC-004,positive,mild,OHC-004-
V02,negative,RNaseP,26.37,RR067e_00758,0
290,OHC-007,positive,mild,OHC-007-
V02,negative,RNaseP,29.58,RR067e_00032,0
291,OHC-007,positive,mild,OHC-007-
V03,negative,RNaseP,26.96,RR067e_00099,0
292,OHC-007,positive,mild,OHC-007-
V04,negative,RNaseP,31.29,RR067e_00218,0
293,OHC-007,positive,mild,OHC-007-
V06,negative,RNaseP,27.18,RR067e_00762,0
294,OHC-008,positive,mild,OHC-008-
V02,negative,RNaseP,33.2,RR067e_00033,0
295,OHC-008,positive,mild,OHC-008-
V03,negative,RNaseP,30.86,RR067e_00101,0
296,OHC-010,positive,mild,OHC-010-
V02,negative,RNaseP,38.01,RR067e_00042,0
297,OHC-010,positive,mild,OHC-010-
V03,negative,RNaseP,36.74,RR067e_00157,0
298,OHC-010,positive,mild,OHC-010-
V04,negative,RNaseP,36.03,RR067e_00212,0

299,OHC-010,positive,mild,OHC-010-
V05,negative,RNaseP,34.99,RR067e_00760,0
300,OHC-014,positive,mild,OHC-014-V03,Invalid,None,NA,RR067e_00158,0
301,OHC-015,positive,mild,OHC-015-
V03,negative,RNaseP,31.5,RR067e_00161,0
302,OHC-018,positive,mild,OHC-018-
V05,negative,RNaseP,27.51,RR067e_00217,0
303,OHC-018,positive,mild,OHC-018-
V09,negative,RNaseP,28.16,RR067e_00768,0
304,OHC-019,positive,mild,OHC-019-
V06,negative,RNaseP,27.37,RR067e_00216,0
305,OHC-019,positive,mild,OHC-019-
V09,negative,RNaseP,27.94,RR067e_00769,0
306,OHC-028,positive,mild,OHC-028-
V0,negative,RNaseP,30.24,RR067e_00756,0
307,OHC-028,positive,mild,OHC-028-
V02,negative,RNaseP,31.67,RR067e_00156,0
308,OHC-030,positive,mild,OHC-030-
V01,negative,RNaseP,29.43,RR067e_00103,0
309,OHC-030,positive,mild,OHC-030-
V06,negative,RNaseP,28.4,RR067e_00755,0
