# Supplementary material for: Anti-nucleocapsid antibody levels and pulmonary comorbid conditions are linked to post–COVID-19 syndrome
Source: JCI Insight. 2022 Jul 8;7(13):e156713. doi: 10.1172/jci.insight.156713 (PMC9310538; doi:10.1172/jci.insight.156713)
Supplement: Supplemental table 5 [file jciinsight-7-156713-s084.pdf]

NexSeq_QC,,,,,,,,,,,,,,,,,,,,,,,,,,,,,,,,,
,stanford_id,Record.ID,CZ.Biohub.ID,Total.Reads,Non.host.Reads,Non.host.Reads..,Total.ERCC.Reads,ERCC.Reads..,X..Pass.CZID.QC,Compression.Ratio,Reads.after.STAR,Reads.after.Trimmomatic,Reads.after.PRICE,Reads.after.De.duplication,host_organism,Insert.Size.Min,Insert.Size.Max,Insert.Size.Mean,Insert.Size.ST.DEV.,Sample.Type,Pseudoaligned.Read.Pairs,Pseudoaligned..,Exon.Read.Pairs,Exon...of.Pseudoaligned,rRNA.Read.Pairs,rRNA...of.Pseudoaligned,ERCC.Read.Pairs,ERCC...of.Pseudoaligned,Flagged.by.FastQC,High.ERCC..30...,High.human.rRNA..50...,Removed.after.background.subtraction,QC.Pass.Fail,control
1,55689-0003 V6,55689-0003,RR067e_00606_W-R310_E1_S98,15989270,18042,0.113,37376,0.23,83.936,3.91,1881492,1706910,1579242,404196,Human,79,14178,214.489,74.7291,Nasopharyngeal Swab,3577544,44.75,3.00E+06,95.4,142224,3.98,21575,0.6,no,no,no,no,pass,0
2,55689-0004-Month3,55689-0004,RR067e_00169_W-R310_A12_S181,112002,590,0.527,16568,14.79,24.558,1.56,56096,21606,13776,8818,Human,90,881,220.58,79.0262,Nasopharyngeal Swab,14912,26.63,5243,35.16,482,3.23,9181,61.57,no,yes,no,no,fail,0
3,55689-0004-V8,55689-0004,RR067e_00743_W-R310_B10_S166,291120,5024,1.726,4430,1.52,59.853,1.97,69216,56290,41428,20980,Human,85,7792,214.184,76.4059,Nasopharyngeal Swab,71342,49.01,65691,92.08,2895,4.06,2747,3.85,no,no,no,no,pass,0
4,55689-0005-V8,55689-0005,RR067e_00744_W-R309_A9_S64,2275180,37514,1.649,10794,0.47,71.52,2.78,406650,369340,290838,104542,Human,80,14208,217.415,76.7476,Nasopharyngeal Swab,529334,46.53,502389,94.91,20427.4,3.86,6359,1.2,no,no,no,no,pass,0
5,55689-0007 V6,55689-0007,RR067e_00604_W-R310_A7_S142,4894,54,1.103,0,0,69.362,2.99,940,728,652,218,Human,90,311,188.901,50.168,Nasopharyngeal Swab,985,40.25,851,86.4,130,13.2,0,0,no,no,no,yes,fail,0
6,55689-0007-V9,55689-0007,RR067e_00731_W-R310_A5_S126,531244,2212,0.416,5570,1.05,63.719,1.94,123614,115606,78766,40516,Human,82,8561,206.591,68.282,Nasopharyngeal Swab,131251,49.41,122454,93.3,5344.82,4.07,3381,2.58,no,no,no,no,pass,0
7,55689-0008 Month 2,55689-0008,RR067e_00124_W-R309_F2_S14,5110908,1684,0.033,32292,0.63,0.493,2.92,5013320,113954,24692,8460,Human,85,4699,222.749,83.5313,Nasopharyngeal Swab,33882,1.33,14442.9,42.63,460.103,1.36,18968,55.98,no,yes,no,no,fail,0
8,55689-0008 V7,55689-0008,RR067e_00627_W-R309_H8_S63,3821192,3150,0.082,8970,0.23,40.623,3.21,1667252,1396414,677292,211002,Human,82,5175,207.86,73.5451,Nasopharyngeal Swab,710962,37.21,680241,95.68,23445.2,3.3,6938,0.98,no,no,no,no,pass,0
9,55689-0009 Month 2,55689-0009,RR067e_00122_W-R311_B6_S228,119066,1686,1.416,7628,6.41,34.458,2.09,89668,59630,30898,14772,Human,82,516,197.724,64.448,Nasopharyngeal

Swab,8187,13.75,2884.63,35.23,45.3726,0.55,5256,64.2,no,yes,no,no,fail
,0
10,55689-0009 V7,55689-0009,RR067e_00628_W-
R309_B2_S10,2775996,88504,3.188,21370,0.77,24.833,2.89,1250362,424380,
310504,107362,Human,82,7288,225.518,82.2976,Nasopharyngeal
Swab,389516,28.06,358719,92.09,18231.7,4.68,12448,3.2,no,no,no,no,pass
,0
11,55689-0010 V6,55689-0010,RR067e_00603_W-
R309_A8_S57,2907170,306572,10.545,9410,0.32,75.459,2.95,931130,881262,
702622,238250,Human,77,9429,204.928,70.304,Nasopharyngeal
Swab,567548,39.04,522775,92.11,38506.5,6.78,6063,1.07,no,no,no,no,pass
,0
12,55689-0010-Month2,55689-0010,RR067e_00194_W-
R310_A10_S165,315530,80588,25.541,5050,1.6,55.776,2.83,187226,119602,1
04428,36958,Human,83,5648,210.985,74.7802,Nasopharyngeal
Swab,36111,22.89,32309,89.47,793,2.2,3009,8.33,no,no,no,no,pass,0
13,55689-0011 Month 2,55689-0011,RR067e_00113_W-
R311_B10_S260,1135788,1832,0.161,23742,2.09,2.458,2.61,1075496,44042,2
6440,10136,Human,85,526,216.677,76.3116,Nasopharyngeal
Swab,19363,3.41,5042,26.04,156,0.81,14165,73.15,yes,yes,no,no,fail,0
14,55689-0011 V6,55689-0011,RR067e_00607_W-
R310_F7_S146,21030,944,4.489,154,0.73,40.185,1.35,6500,2700,2612,1938,
Human,84,9095,207.195,66.3985,Nasopharyngeal
Swab,4269,40.6,3825,89.6,359,8.41,83,1.94,no,no,no,no,pass,0
15,55689-0011-V9,55689-0011,RR067e_00719_W-
R309_F4_S30,194350,5072,2.61,464,0.24,50.469,1.9,83734,66686,42260,222
94,Human,85,6737,189.054,60.8002,Nasopharyngeal
Swab,37545,38.64,36253.7,96.56,928.323,2.47,321,0.85,no,no,no,no,pass,
0
16,55689-0012 V6,55689-0012,RR067e_00605_W-
R309_D6_S44,2578596,43288,1.679,7948,0.31,46.771,4.24,561686,303154,26
2704,61992,Human,78,9515,219.705,77.271,Nasopharyngeal
Swab,526097,40.8,504711,95.93,16766,3.19,4563,0.87,no,no,no,no,pass,0
17,55689-0013 Month 2,55689-0013,RR067e_00114_W-
R311_G9_S257,597986,2104,0.352,75312,12.59,16.345,2.83,389210,112964,6
3616,22492,Human,86,2369,207.99,68.5881,Nasopharyngeal
Swab,77115,25.79,31568,40.94,566,0.73,44969,58.31,no,yes,no,no,fail,0
18,55689-0013-Month3,55689-0013,RR067e_00171_W-
R311_F11_S272,333040,2422,0.727,38676,11.61,5.373,2.8,280374,23740,150
64,5372,Human,86,645,213.209,74.7577,Nasopharyngeal
Swab,26862,16.13,3934,14.65,218,0.81,22679,84.43,no,yes,no,no,fail,0
19,55689-0013-V8,55689-0013,RR067e_00709_W-
R311_G1_S193,2375960,21016,0.885,39428,1.66,60.473,2.79,690184,678536,
417374,149614,Human,79,6735,208.456,71.9535,Nasopharyngeal
Swab,559335,47.08,463224,82.82,71374.9,12.76,23075,4.13,no,no,no,no,pa
ss,0
20,55689-0013-V9,55689-0013,RR067e_00723_W-
R309_G4_S31,3494320,8032,0.23,18056,0.52,54.59,4.57,757960,558924,4137
74,90520,Human,79,7759,215.116,78.3999,Nasopharyngeal
Swab,721764,41.31,696395,96.49,15527.7,2.15,9735,1.35,no,no,no,no,pass

,0
21,55689-0017 V8,55689-0017,RR067e_00653_W-
R309_F11_S85,5235712,5146,0.098,13486,0.26,12.199,2.44,3277632,637294,
399834,163596,Human,80,9402,211.738,75.037,Nasopharyngeal
Swab,539500,20.61,509517,94.44,21451.4,3.98,8345,1.55,no,no,no,no,pass
,0
22,55689-0017-Month3,55689-0017,RR067e_00179_W-
R310_F9_S162,13372322,3824,0.029,9128,0.07,0.178,2.9,13336932,166254,2
3752,8180,Human,87,544,195.181,43.677,Nasopharyngeal
Swab,11397,0.17,5773,50.65,83,0.73,5541,48.62,no,yes,no,no,fail,0
23,55689-0017-Month4,55689-0017,RR067e_00198_W-
R309_D4_S28,3355150,10082,0.3,6966,0.21,67.327,2.58,672724,643644,4529
22,175300,Human,78,12963,213.125,74.8264,Nasopharyngeal
Swab,620793,37.01,583530,94,32851.4,5.29,4097,0.66,no,no,no,no,pass,0
24,55689-0017-V9,55689-0017,RR067e_00695_W-
R309_G11_S86,6374998,1860,0.029,3884,0.06,3.474,2.25,5282080,282790,18
3518,81458,Human,83,6870,214.031,76.2868,Nasopharyngeal
Swab,312889,9.82,297486,95.08,12837,4.1,2454,0.78,no,no,no,no,pass,0
25,55689-0018-Month4,55689-0018,RR067e_00196_W-
R311_B11_S268,245410,2172,0.885,9038,3.68,5.686,1.7,232800,27340,13236
,7802,Human,89,741,212.686,74.0268,Nasopharyngeal
Swab,7174,5.85,1106,15.42,13,0.18,6053,84.37,yes,yes,no,no,fail,0
26,55689-0022 Month 2,55689-0022,RR067e_00138_W-
R309_G7_S55,2345672,2226054,94.9,7416,0.32,96.71,5.1,2320086,2310528,2
243754,439978,Human,84,798,205.206,67.106,Nasopharyngeal
Swab,8582,0.73,4243,49.44,41,0.48,4280,49.87,no,yes,no,no,fail,0
27,55689-0022 V6,55689-0022,RR067e_00614_W-
R309_G10_S78,4467578,3820,0.086,4004,0.09,3.559,2.44,3837342,208770,13
6558,56040,Human,83,8020,210.865,73.1009,Nasopharyngeal
Swab,203730,9.12,187182,91.88,14120.7,6.93,2356,1.16,no,no,no,no,pass,
0
28,55689-0023 Month 2,55689-0023,RR067e_00139_W-
R310_G7_S147,3070780,2726370,88.784,3216,0.1,89.3,5.28,3065358,2805084
,2737374,518304,Human,90,492,227.577,73.2299,Nasopharyngeal
Swab,2339,0.15,556,23.77,14,0.6,1769,75.63,no,yes,no,no,fail,0
29,55689-0023-D0,55689-0023,RR067e_00025_W-
R311_B3_S204,642472,11434,1.78,5642,0.88,15.926,2.47,299538,57624,4770
4,19332,Human,82,6839,223.452,80.867,Nasopharyngeal
Swab,104080,32.4,94970,91.25,5885,5.65,3215,3.09,no,no,no,no,pass,0
30,55689-0024 Month 2,55689-0024,RR067e_00140_W-
R310_H4_S125,2600384,2442246,93.919,4728,0.18,95.436,4.74,2587554,2562
514,2469452,520652,Human,92,615,212.919,74.6152,Nasopharyngeal
Swab,5055,0.39,2357,46.63,49,0.97,2644,52.3,no,yes,no,no,fail,0
31,55689-0024 V6,55689-0024,RR067e_00617_W-
R309_A3_S17,3836086,5698,0.149,1782,0.05,69.96,1.68,880684,858908,6161
30,365892,Human,80,14178,204.544,69.6301,Nasopharyngeal
Swab,801288,41.78,752905,93.96,47064.6,5.87,1067,0.13,no,no,no,no,pass
,0
32,55689-0024-D30,55689-0024,RR067e_00049_W-
R311_E7_S239,3418774,5174,0.151,20576,0.6,0.999,3.25,3306254,197056,33

042,10164,Human,89,490,205.623,71.106,Nasopharyngeal
Swab,42094,2.46,28687.2,68.15,1305.76,3.1,12084,28.71,no,no,no,yes,fai
l,0
33,55689-0024-Month3,55689-0024,RR067e_00184_W-
R311_A8_S243,314054,2254,0.718,34680,11.04,17.888,3.18,259380,102446,4
6398,14568,Human,88,451,218.039,72.1871,Nasopharyngeal
Swab,32100,20.44,5536,17.25,24.0042,0.07,26540,82.68,no,yes,no,no,fail
,0
34,55689-0024-Month4,55689-0024,RR067e_00203_W-
R309_A1_S1,6028836,4810,0.08,1412,0.02,35.226,2.4,2963328,2015250,1043
870,434428,Human,79,12408,206.653,71.0751,Nasopharyngeal
Swab,877768,29.12,830564,94.62,45787.2,5.22,1080,0.12,no,no,no,no,pass
,0
35,55689-0024-V8,55689-0024,RR067e_00699_W-
R309_E5_S37,4660408,18562,0.398,5684,0.12,61.53,2.62,1433942,1413274,8
82310,337316,Human,78,9360,208.746,72.2047,Nasopharyngeal
Swab,1105771,47.45,1.00E+06,94.47,57591.7,5.21,3495,0.32,no,no,no,no,p
ass,0
36,55689-0026 Month 2,55689-0026,RR067e_00132_W-
R310_D9_S160,140166,368,0.263,18446,13.16,8.717,1.67,107084,16524,9334
,5606,Human,88,586,225.37,71.6677,Nasopharyngeal
Swab,13900,19.83,3454,24.85,141,1.01,10305,74.14,yes,yes,no,no,fail,0
37,55689-0026 V7,55689-0026,RR067e_00641_W-
R310_D5_S129,111450,1166,1.046,1456,1.31,49.221,1.71,51498,46738,25348
,14854,Human,84,1263,194.62,62.1154,Nasopharyngeal
Swab,24905,44.69,21817,87.6,2041.98,8.2,1038,4.17,no,no,no,no,pass,0
38,55689-0029 V7,55689-0029,RR067e_00654_W-
R310_G9_S163,447198,5352,1.197,2254,0.5,5.998,2.27,339592,27610,20368,
8968,Human,86,5224,214.098,75.4514,Nasopharyngeal
Swab,30529,13.65,27652,90.58,1531,5.01,1332,4.36,no,no,no,no,pass,0
39,55689-0029-Month2,55689-0029,RR067e_00185_W-
R311_G10_S265,4413442,966,0.022,10238,0.23,0.297,4.49,4397196,18660,13
074,2912,Human,89,535,233.81,86.5455,Nasopharyngeal
Swab,10203,0.46,3895,38.18,62,0.61,6246,61.22,yes,yes,no,no,fail,0
40,55689-0031 Month 2,55689-0031,RR067e_00144_W-
R311_A5_S219,198856,5378,2.704,23160,11.65,9.04,3.38,147456,15790,1333
0,3946,Human,86,573,213.31,71.8318,Nasopharyngeal
Swab,17586,17.69,4414,25.1,157,0.89,13009,73.97,no,yes,no,no,fail,0
41,55689-0031-D0,55689-0031,RR067e_00035_W-
R311_H3_S210,97458,1844,1.892,7916,8.12,46.721,1.94,33488,22440,15646,
8076,Human,87,2517,215.673,78.4898,Nasopharyngeal
Swab,16582,34.03,11191,67.49,842,5.08,4516,27.23,no,no,no,no,pass,0
42,55689-0032 V6,55689-0032,RR067e_00609_W-
R309_A6_S41,2690072,67890,2.524,43194,1.61,60.086,3.2,514220,338066,30
8976,96520,Human,82,8475,213.734,73.2465,Nasopharyngeal
Swab,621975,46.24,556153,89.42,41424.6,6.66,24172,3.89,no,no,no,no,pas
s,0
43,55689-0033 Month 2,55689-0033,RR067e_00137_W-
R311_G5_S225,103890,268,0.258,26550,25.56,5.877,5.57,77278,6634,4542,8
16,Human,92,638,196.984,57.6578,Nasopharyngeal

Swab,15629,30.09,410,2.62,1,0.01,15218,97.37,no,yes,no,no,fail,0
44,55689-0033 V6,55689-0033,RR067e_00745_W-
R310_C4_S120,1173184,153090,13.049,15668,1.34,21.228,6.67,927650,21854
6,196918,29516,Human,85,11288,218.807,79.6523,Nasopharyngeal
Swab,94060,16.03,72649,77.24,11888,12.64,9441,10.04,no,no,no,no,pass,0
45,55689-0033-D0,55689-0033,RR067e_00026_W-
R311_B8_S244,2619246,1122,0.043,578,0.02,0.159,1.87,2616086,61940,4162
,2230,Human,91,638,201.884,68.8284,Nasopharyngeal
Swab,1656,0.13,1144,69.08,96,5.8,416,25.12,no,no,no,no,pass,0
46,55689-0033-Month4,55689-0033,RR067e_00195_W-
R310_G5_S132,241768,20192,8.352,2310,0.96,90.208,2.02,46182,44968,4166
0,20600,Human,83,7348,217.382,74.0054,Nasopharyngeal
Swab,50273,41.59,45197,89.9,3751,7.46,1307,2.6,no,no,no,no,pass,0
47,55689-0034-D0,55689-0034,RR067e_00044_W-
R311_F10_S264,554146,11696,2.111,37328,6.74,14.517,3.23,384414,85312,5
5806,17276,Human,83,4584,200.034,63.488,Nasopharyngeal
Swab,60796,21.94,31857,52.4,7597.95,12.5,21290,35.02,no,yes,no,no,fail
,0
48,55689-0035 V7,55689-0035,RR067e_00650_W-
R309_A12_S88,1642560,10702,0.652,6422,0.39,77.532,3.16,254380,243516,1
97226,62426,Human,81,9519,216.737,77.2873,Nasopharyngeal
Swab,350421,42.67,325428,92.87,21015.4,6,3776,1.08,no,no,no,no,pass,0
49,55689-0035-D0,55689-0035,RR067e_00053_W-
R309_H3_S24,2368680,81332,3.434,27110,1.14,54.142,3.38,600566,478106,3
25160,96190,Human,80,6731,230.223,85.8888,Nasopharyngeal
Swab,374370,31.61,340633,90.99,18108.2,4.84,15601,4.17,no,no,no,no,pas
s,0
50,55689-0035-D30,55689-0035,RR067e_00021_W-
R311_A3_S203,469960,226,0.048,11330,2.41,42.544,1.64,232636,209088,989
72,60304,Human,83,8614,221.208,78.7059,Nasopharyngeal
Swab,56933,24.23,48414.6,85.04,1284.37,2.26,7185,12.62,no,no,no,yes,fa
il,0
51,55689-0035-Month3,55689-0035,RR067e_00193_W-
R310_F1_S99,11881906,1108722,9.331,112358,0.95,55.832,7.9,3977134,2644
650,2220494,281190,Human,78,12412,215.784,74.9062,Nasopharyngeal
Swab,2194284,36.93,2.00E+06,90.15,150079,6.84,63965,2.92,no,no,no,no,p
ass,0
52,55689-0037 V5,55689-0037,RR067e_00601_W-
R309_B4_S26,5110230,252936,4.95,61410,1.2,17.761,4.9,3407438,747866,60
5190,123568,Human,81,7813,209.549,72.4803,Nasopharyngeal
Swab,422593,16.54,359420,85.05,26783.4,6.34,35955,8.51,no,no,no,no,pas
s,0
53,55689-0037-D0,55689-0037,RR067e_00009_W-
R310_G6_S140,243888,31468,12.903,8348,3.42,71.406,2.5,87718,80756,6263
6,25020,Human,83,4695,219.957,76.1152,Nasopharyngeal
Swab,46622,38.23,37709.2,80.88,4086.76,8.77,4788,10.27,no,no,no,no,pas
s,0
54,55689-0037-V8,55689-0037,RR067e_00740_W-
R310_C2_S104,206886,23174,11.201,2010,0.97,21.779,2.65,142594,32982,31
056,11714,Human,84,6241,217.865,76.9279,Nasopharyngeal

Swab,16721,16.16,14685,87.82,933,5.58,1102,6.59,no,no,no,no,pass,0
55,55689-0038-D0,55689-0038,RR067e_00054_W-
R311_F1_S192,1516496,9980,0.658,30106,1.99,31.019,3.25,650388,311486,2
01742,61992,Human,83,6156,207.507,72.0923,Nasopharyngeal
Swab,262431,34.61,219798,83.75,24117.9,9.19,18412,7.02,no,no,no,no,pas
s,0
56,55689-0039-D0,55689-0039,RR067e_00017_W-
R311_A10_S259,451974,26088,5.772,45052,9.97,24.047,3.55,305546,115374,
73476,20724,Human,88,494,211.944,72.5871,Nasopharyngeal
Swab,46447,20.55,17634,37.97,1678,3.61,27105,58.36,no,yes,no,no,fail,0
57,55689-0039-Month2,55689-0039,RR067e_00189_W-
R311_A1_S187,195868,78214,39.932,8284,4.23,73.021,2.64,132920,109418,9
7060,36736,Human,85,1773,204.745,73.679,Nasopharyngeal
Swab,16929,17.29,10302,60.85,1689,9.98,4928,29.11,no,no,no,no,pass,0
58,55689-0040 V5,55689-0040,RR067e_00588_W-
R309_E3_S21,3590316,11002,0.306,2360,0.07,79.457,2.44,614342,589818,48
8136,199928,Human,81,14181,200.748,68.5136,Nasopharyngeal
Swab,638956,35.59,620036,97.04,17346.6,2.71,1468,0.23,no,no,no,no,pass
,0
59,55689-0040 V7,55689-0040,RR067e_00640_W-
R309_H6_S48,3783488,24288,0.642,10316,0.27,43.502,2.5,1496390,1082728,
650964,260042,Human,79,9858,209.387,73.5583,Nasopharyngeal
Swab,760301,40.19,675475,88.84,77579.3,10.2,6868,0.9,no,no,no,no,pass,
0
60,55689-0040-D0,55689-0040,RR067e_00018_W-
R310_E6_S138,64274,618,0.962,1152,1.79,31.512,1.55,41470,27574,13068,8
418,Human,90,6046,212.162,75.6482,Nasopharyngeal
Swab,6467,20.12,5350,82.73,319,4.93,795,12.29,no,no,no,yes,fail,0
61,55689-0040-D30,55689-0040,RR067e_00005_W-
R311_E2_S199,1362722,858,0.063,25712,1.89,46.65,2.77,680612,656144,317
508,114666,Human,85,2944,214.508,73.8456,Nasopharyngeal
Swab,216530,31.78,193780,89.49,5307.85,2.45,17231,7.96,no,no,no,no,pas
s,0
62,55689-0042-D30,55689-0042,RR067e_00073_W-
R311_C10_S261,110566,100,0.09,1628,1.47,0.609,2.3,107364,788,654,284,H
uman,90,481,210.734,71.3152,Nasopharyngeal
Swab,1160,2.1,202,17.41,11,0.95,947,81.64,no,yes,no,no,fail,0
63,55689-0042-V8,55689-0042,RR067e_00672_W-
R309_A7_S49,4446428,117880,2.651,21296,0.48,22.1,3.6,2871670,1259596,6
34634,176146,Human,82,2964,224.395,80.8007,Nasopharyngeal
Swab,402655,18.11,365807,90.85,21477.1,5.33,15289,3.8,no,no,no,no,pass
,0
64,55689-0043 V5,55689-0043,RR067e_00590_W-
R310_E10_S169,360448,80194,22.248,8114,2.25,55.719,3.35,183810,108874,
102418,30606,Human,83,8286,218.68,77.7701,Nasopharyngeal
Swab,39244,21.78,32789.1,83.55,1757.9,4.48,4644,11.83,no,no,no,no,pass
,0
65,55689-0044 V7,55689-0044,RR067e_00656_W-
R309_H4_S32,1920686,2798,0.146,2050,0.11,85.049,1.62,236052,233130,200
760,124142,Human,82,8174,219.524,76.0133,Nasopharyngeal

Swab,513077,53.43,480763,93.7,31060.2,6.05,1150,0.22,no,no,no,no,pass,
0
66,55689-0044-D0,55689-0044,RR067e_00002_W-
R309_D8_S60,4353164,11360,0.261,11886,0.27,23.112,3.78,2199422,675670,
508330,134328,Human,80,8910,195.341,64.5263,Nasopharyngeal
Swab,628520,28.88,584812,93.05,35621.9,5.67,7542,1.2,no,no,no,no,pass,
0
67,55689-0044-D30,55689-0044,RR067e_00075_W-
R311_H6_S234,108028,152,0.141,3258,3.02,23.045,1.72,42014,14372,9682,5
634,Human,83,888,220.7,79.5683,Nasopharyngeal
Swab,16663,30.85,14011.1,84.09,658.912,3.95,1956,11.74,no,no,no,yes,fa
il,0
68,55689-0044-Month3,55689-0044,RR067e_00187_W-
R310_G11_S179,146824,234,0.159,6246,4.25,3.977,1.67,103508,5000,4116,2
460,Human,84,954,233.539,83.6388,Nasopharyngeal
Swab,18119,24.68,14325,79.06,413,2.28,3369,18.59,no,no,no,yes,fail,0
69,55689-0046 V7,55689-0046,RR067e_00662_W-
R310_G2_S108,1383282,154472,11.167,21148,1.53,44.324,5.51,546620,25170
8,242286,43934,Human,80,6815,222.566,80.5052,Nasopharyngeal
Swab,257147,37.18,230187,89.52,15098.5,5.87,11834,4.6,no,no,no,no,pass
,0
70,55689-0046-D0,55689-0046,RR067e_00003_W-
R309_F7_S54,45316,4442,9.802,836,1.84,72.404,1.48,12154,9122,8800,5946
,Human,88,6746,208.165,68.4245,Nasopharyngeal
Swab,8902,39.29,8005,89.92,402,4.52,494,5.55,no,no,no,no,pass,0
71,55689-0046-Month3,55689-0046,RR067e_00192_W-
R309_C5_S35,4632786,153190,3.307,19636,0.42,49.916,3.37,1963786,184042
2,980244,290604,Human,80,8634,217.246,79.4468,Nasopharyngeal
Swab,652545,28.17,603923,92.55,35205.1,5.4,13363,2.05,no,no,no,no,pass
,0
72,55689-0047 V5,55689-0047,RR067e_00591_W-
R310_D7_S144,155386,4068,2.618,2656,1.71,44.974,1.74,79334,69286,35680
,20560,Human,82,9115,228.132,85.9053,Nasopharyngeal
Swab,21140,27.21,18859,89.21,546,2.58,1717,8.12,no,no,no,no,pass,0
73,55689-0047-D0,55689-0047,RR067e_00010_W-
R311_B1_S188,1574346,15862,1.008,28148,1.79,5.694,5.89,1115990,71300,6
3550,10798,Human,81,1053,230.73,85.0471,Nasopharyngeal
Swab,100928,12.82,80662.9,79.92,4498.11,4.46,15767,15.62,no,no,no,no,p
ass,0
74,55689-0047-V8,55689-0047,RR067e_00705_W-
R310_G3_S116,422210,22844,5.411,3854,0.91,40.257,1.74,213968,149444,86
138,49538,Human,84,3016,224.332,81.7789,Nasopharyngeal
Swab,67523,31.99,60974,90.3,4097,6.07,2419,3.58,no,no,no,no,pass,0
75,55689-0049 V6,55689-0049,RR067e_00620_W-
R309_D9_S67,107708,3336,3.097,1350,1.25,59.588,1.76,32268,28334,19228,
10934,Human,84,7129,210.292,70.9182,Nasopharyngeal
Swab,20224,37.55,18451,91.23,959,4.74,796,3.94,no,no,no,no,pass,0
76,55689-0050 V7,55689-0050,RR067e_00648_W-
R310_C12_S183,354038,62746,17.723,12940,3.65,73.641,2.55,138034,131524
,101650,39880,Human,82,6756,221.845,79.9093,Nasopharyngeal

Swab,81020,45.77,68547.2,84.61,4220.82,5.21,8113,10.01,no,no,no,no,pas
s,0
77,55689-0051 V7,55689-0051,RR067e_00634_W-
R310_A11_S173,347686,220,0.063,1744,0.5,33.118,2.01,91130,37542,30180,
14992,Human,81,6862,219.342,77.4119,Nasopharyngeal
Swab,76650,44.09,73006.1,95.25,2659.91,3.47,979,1.28,no,no,no,yes,fail
,0
78,55689-0051-D30,55689-0051,RR067e_00081_W-
R311_D6_S230,167302,96,0.057,6064,3.62,17.19,2.66,86994,22560,14954,56
20,Human,90,462,199.952,65.6922,Nasopharyngeal
Swab,16736,20.01,12957,77.42,437,2.61,3342,19.97,no,no,no,no,pass,0
79,55689-0051-Month3,55689-0051,RR067e_00204_W-
R309_G3_S23,2898666,220,0.008,3576,0.12,66.552,2.25,498122,379484,3315
08,147324,Human,77,11183,205.471,70.5709,Nasopharyngeal
Swab,535650,36.96,513008,95.77,20376.5,3.8,2167,0.4,no,no,no,yes,fail,
0
80,55689-0051-V8,55689-0051,RR067e_00729_W-
R309_C6_S43,4814728,582,0.012,7732,0.16,53.662,3,1316756,1010332,70660
2,235190,Human,79,8597,203.152,67.5016,Nasopharyngeal
Swab,1139148,47.32,1100000,96.4,35875.1,3.15,4938,0.43,no,no,no,no,pas
s,0
81,55689-0052-D0,55689-0052,RR067e_00037_W-
R311_H11_S274,451478,3582,0.793,67666,14.99,33.584,2.98,316766,240412,
106384,35742,Human,88,909,224.508,79.5382,Nasopharyngeal
Swab,51141,22.65,8362,16.35,472,0.92,42307,82.73,no,yes,no,no,fail,0
82,55689-0054-D0,55689-0054,RR067e_00055_W-
R311_A11_S267,2714256,7932,0.292,43294,1.6,2.719,3.52,2375014,84294,64
584,18366,Human,80,6514,214.672,77.4431,Nasopharyngeal
Swab,98310,7.24,63272,64.36,10395,10.57,24586,25.01,yes,no,no,no,fail,
0
83,55689-0056 V6,55689-0056,RR067e_00624_W-
R310_G8_S155,3131042,321388,10.265,38584,1.23,20.19,5.36,2281478,57909
4,460630,85960,Human,84,6407,233.311,88.5635,Nasopharyngeal
Swab,243305,15.54,213538,87.77,7719.25,3.17,22042,9.06,no,no,no,no,pas
s,0
84,55689-0056 V7,55689-0056,RR067e_00655_W-
R311_D7_S238,53646,7928,14.778,1390,2.59,19.18,2.64,47216,10212,9056,3
432,Human,91,737,218.734,79.5162,Nasopharyngeal
Swab,1505,5.61,623,41.4,25,1.66,857,56.94,no,yes,no,no,fail,0
85,55689-0057-D0,55689-0057,RR067e_00062_W-
R310_F8_S154,6386076,83994,1.315,73810,1.16,69.462,5.4,1410266,1250156
,979592,181502,Human,78,8185,205.006,69.5804,Nasopharyngeal
Swab,1698266,53.19,2.00E+06,91.93,92694.5,5.46,44171,2.6,no,no,no,no,p
ass,0
86,55689-0057-V8,55689-0057,RR067e_00694_W-
R311_E8_S247,62266,2070,3.324,1470,2.36,42.266,1.66,27800,18576,11750,
7072,Human,82,1941,205.164,71.1762,Nasopharyngeal
Swab,11909,38.25,10026,84.19,955,8.02,927,7.78,no,no,no,no,pass,0
87,55689-0058 Month 2,55689-0058,RR067e_00152_W-
R310_D10_S168,52240,40,0.077,2374,4.54,3.941,2.03,38878,2174,1532,754,

Human,86,602,216.853,67.2722,Nasopharyngeal
Swab,4095,15.68,2737,66.84,22,0.54,1336,32.63,yes,yes,no,no,fail,0
88,55689-0058 V7,55689-0058,RR067e_00660_W-
R309_E8_S61,2778356,22386,0.806,11452,0.41,13.184,3.14,1726746,292918,
227656,72532,Human,79,7055,202.185,67.5409,Nasopharyngeal
Swab,395478,28.47,363705,91.97,24440,6.18,7148,1.81,no,no,no,no,pass,0
89,55689-0058-D30,55689-0058,RR067e_00085_W-
R310_C5_S128,2355470,2123614,90.157,25534,1.08,93.03,5.38,2314302,2247
900,2153006,399950,Human,88,715,222.563,79.0271,Nasopharyngeal
Swab,17575,1.49,2890.1,16.44,157.899,0.9,14527,82.66,no,yes,no,no,fail
,0
90,55689-0059-D0,55689-0059,RR067e_00038_W-
R311_C5_S221,156498,1564,0.999,2926,1.87,39.864,1.81,92358,76200,36818
,20320,Human,87,2182,214.052,79.9651,Nasopharyngeal
Swab,17076,21.82,14467,84.72,433,2.54,2162,12.66,no,no,no,no,pass,0
91,55689-0060-D30,55689-0060,RR067e_00120_W-
R311_D10_S262,3960,2,0.051,868,21.92,11.028,1.64,2412,420,266,162,Huma
n,103,706,214.897,73.1776,Nasopharyngeal
Swab,540,27.27,48,8.89,0,0,492,91.11,no,yes,no,no,fail,0
92,55689-0062 V5,55689-0062,RR067e_00589_W-
R309_G1_S7,3734044,241456,6.466,11918,0.32,63.21,4.41,1006508,844686,6
36210,144148,Human,82,4319,220.511,79.4313,Nasopharyngeal
Swab,575969,30.85,555315,96.41,13660.8,2.37,6922,1.2,no,no,no,no,pass,
1
93,55689-0062-D0,55689-0062,RR067e_00020_W-
R310_A4_S118,548962,32282,5.881,1588,0.29,7.179,3.08,537770,48616,3860
4,12552,Human,96,627,227.94,80.5832,Nasopharyngeal
Swab,2196,0.8,1127,51.32,26,1.18,1043,47.5,warning,yes,no,no,fail,1
94,55689-0062-D30,55689-0062,RR067e_00077_W-
R310_F3_S115,196144,83028,42.33,2156,1.1,77.978,2.56,151404,149872,118
062,46096,Human,83,1216,208.26,69.2066,Nasopharyngeal
Swab,8906,9.08,7389.78,82.98,124.219,1.39,1386,15.56,no,no,no,no,pass,
1
95,55689-0065-D0,55689-0065,RR067e_00030_W-
R311_B12_S276,50308,238,0.473,3896,7.74,5.832,1.84,38612,4358,2252,122
2,Human,90,2886,203.895,69.9684,Nasopharyngeal
Swab,4412,17.54,1915,43.4,203,4.6,2294,51.99,no,yes,no,no,fail,0
96,55689-0065-D30,55689-0065,RR067e_00106_W-
R310_C8_S151,9027574,1184,0.013,61484,0.68,24.222,4.49,3826458,1388198
,926844,206236,Human,81,9606,208.784,68.59,Nasopharyngeal
Swab,1224722,27.13,1.00E+06,93.83,37782.2,3.08,37221,3.04,no,no,no,yes
,fail,0
97,55689-0066-D0,55689-0066,RR067e_00048_W-
R311_F6_S232,269340,324,0.12,1512,0.56,1.165,1.55,263606,8384,3072,198
4,Human,89,356,209.378,66.3385,Nasopharyngeal
Swab,1815,1.35,902,49.7,25,1.38,888,48.93,yes,yes,no,no,fail,0
98,55689-0067-D0,55689-0067,RR067e_00041_W-
R311_E6_S231,4784,902,18.855,20,0.42,19.074,1.26,4750,942,906,720,Huma
n,175,348,241.1,60.1525,Nasopharyngeal
Swab,22,0.92,12,54.55,0,0,10,45.45,warning,yes,no,no,fail,0

99,55689-0067-D30,55689-0067,RR067e_00088_W-R310_E11_S177,416510,205158,49.256,47556,11.42,74.89,3.47,314996,262796,235902,68030,Human,82,849,215.252,72.7888,Nasopharyngeal Swab,31813,15.28,4476.52,14.07,171.482,0.54,27162,85.38,no,yes,no,no,fail,0
100,55689-0072 V7,55689-0072,RR067e_00599_W-R309_C10_S74,6280744,649812,10.346,35324,0.56,13.45,6.89,5915860,922796,795690,115414,Human,83,1053,202.412,70.4181,Nasopharyngeal Swab,131911,4.2,104033,78.87,6383.78,4.84,21344,16.18,no,no,no,no,pass,0
101,55689-0072-V8,55689-0072,RR067e_00687_W-R309_A10_S72,4024180,157070,3.903,77944,1.94,50.417,7.06,1215758,710326,612944,86784,Human,79,8205,192.764,60.951,Nasopharyngeal Swab,841198,41.81,725634,86.26,71150.9,8.46,43687,5.19,no,no,no,no,pass,0
102,55689-0073 V6,55689-0073,RR067e_00626_W-R310_B4_S119,512190,14384,2.808,3974,0.78,69.433,2.03,133634,129020,92786,45782,Human,80,6883,211.541,71.9289,Nasopharyngeal Swab,108724,42.45,97218,89.42,8954.99,8.24,2500,2.3,no,no,no,no,pass,0
103,55689-0073 V7,55689-0073,RR067e_00636_W-R309_E11_S84,10228,36,0.352,5692,55.65,5.74,2.6,4530,350,260,100,Human,107,649,248.957,80.0391,Nasopharyngeal Swab,2965,57.98,8,0.27,0,0,2957,99.73,no,yes,no,no,fail,0
104,55689-0073-D0,55689-0073,RR067e_00064_W-R311_D11_S270,2717146,7936,0.292,61268,2.25,7.395,3.27,2510324,411238,185650,56710,Human,86,1272,209.085,74.3093,Nasopharyngeal Swab,80410,5.92,34490.6,42.89,2841.42,3.53,43050,53.54,no,yes,no,no,fail,0
105,55689-0073-D30,55689-0073,RR067e_00092_W-R310_D11_S176,151672,90,0.059,3076,2.03,51.037,1.6,36828,34312,18796,11752,Human,82,6107,226.264,83.1939,Nasopharyngeal Swab,28839,38.03,25589,88.73,1399.95,4.85,1823,6.32,no,no,no,yes,fail,0
106,55689-0074 V6,55689-0074,RR067e_00631_W-R310_C1_S96,355860,7026,1.974,3828,1.08,50.181,1.79,134398,125104,67442,37590,Human,84,6925,218.628,75.5471,Nasopharyngeal Swab,69667,39.15,62779,90.11,4416,6.34,2439,3.5,no,no,no,no,pass,0
107,55689-0074-D0,55689-0074,RR067e_00059_W-R311_E5_S223,1344468,1514,0.113,40234,2.99,10.366,2.9,1226314,287048,127116,43792,Human,88,468,210.259,57.6521,Nasopharyngeal Swab,38945,5.79,13496,34.65,310.027,0.8,25138,64.55,no,yes,no,no,fail,0
108,55689-0074-D30,55689-0074,RR067e_00093_W-R311_F3_S208,4140426,7254,0.175,191796,4.63,20.016,3.75,2276128,850616,455598,121434,Human,83,6731,216.429,72.767,Nasopharyngeal Swab,489685,23.65,359836,73.48,15434.6,3.15,113828,23.25,no,no,no,no,pass,0
109,55689-0074-Month3,55689-0074,RR067e_00170_W-R311_E10_S263,1010708,5316,0.526,59172,5.85,4.396,3.46,890772,65162,39158,11326,Human,94,507,206.742,69.813,Nasopharyngeal

Swab,44173,8.74,6583.23,14.9,209.766,0.47,37380,84.62,yes,yes,no,no,fa
il,0
110,55689-0076 V7,55689-0076,RR067e_00659_W-
R311_G2_S201,272692,9108,3.34,3174,1.16,56.867,2.59,50328,33128,28620,
11062,Human,85,6849,229.516,84.5921,Nasopharyngeal
Swab,72915,53.48,67855,93.06,3254,4.46,1794,2.46,no,no,no,no,pass,0
111,55689-0076-D30,55689-0076,RR067e_00082_W-
R311_H9_S258,37718,186,0.493,4766,12.64,4.602,1.87,31206,3132,1436,768
,Human,89,631,220.328,76.9912,Nasopharyngeal
Swab,2928,15.53,256.942,8.78,4.05812,0.14,2667,91.09,no,yes,no,no,fail
,0
112,55689-0081 V6,55689-0081,RR067e_00595_W-
R310_G4_S124,569404,3236,0.568,7310,1.28,55.182,1.96,113646,86380,6271
2,31960,Human,81,9453,222.246,81.6036,Nasopharyngeal
Swab,134254,47.16,122531,91.27,7384.44,5.5,4209,3.14,no,no,no,no,pass,
0
113,55689-0082 V6,55689-0082,RR067e_00625_W-
R311_H4_S218,73906,3138,4.246,3412,4.62,16.026,1.79,55586,13958,8908,4
968,Human,87,795,223.734,80.3979,Nasopharyngeal
Swab,5914,16,3680,62.23,179,3.03,2055,34.75,no,yes,no,no,fail,0
114,55689-0082-D30,55689-0082,RR067e_00129_W-
R311_C9_S253,439148,38522,8.772,36246,8.25,41.077,2.75,324260,242030,1
33196,48400,Human,87,536,216.064,75.2695,Nasopharyngeal
Swab,31548,14.37,8279.39,26.24,38.6122,0.12,23226,73.62,no,yes,no,no,f
ail,0
115,55689-0082-Month2,55689-0082,RR067e_00167_W-
R311_D9_S254,35558,212,0.596,3156,8.88,8.18,1.48,30000,4302,2454,1654,
Human,88,550,207.808,80.9461,Nasopharyngeal
Swab,2174,12.23,213,9.8,0,0,1961,90.2,no,yes,no,no,fail,0
116,55689-0085 V5,55689-0085,RR067e_00612_W-
R310_C6_S136,2446352,117382,4.798,7228,0.3,10.476,3.97,2106922,345066,
220712,55610,Human,85,775,221.486,80.1287,Nasopharyngeal
Swab,59750,4.88,52039.3,87.1,3491.66,5.84,4219,7.06,warning,no,no,no,p
ass,0
117,55689-0090-D30,55689-0090,RR067e_00125_W-
R311_A9_S251,2719388,33212,1.221,63236,2.33,3.78,3.66,2514794,178230,9
5070,26002,Human,87,604,211.178,73.8462,Nasopharyngeal
Swab,58729,4.32,20258.8,34.5,743.17,1.27,37721,64.23,no,yes,no,no,fail
,0
118,55689-0091 V6,55689-0091,RR067e_00637_W-
R309_B6_S42,3286754,16722,0.509,8528,0.26,13.025,3.03,1708136,285310,2
22486,73312,Human,81,8108,216.866,77.4231,Nasopharyngeal
Swab,384022,23.37,361462,94.13,17249.9,4.49,5229,1.36,no,no,no,no,pass
,0
119,55689-0093 V5,55689-0093,RR067e_00608_W-
R311_B5_S220,4535196,197170,4.348,58324,1.29,44.96,4.84,1585116,834182
,712676,147204,Human,81,8645,213.036,75.5862,Nasopharyngeal
Swab,843229,37.19,703149,83.39,105905,12.56,33152,3.93,no,no,no,no,pas
s,0
120,55689-0093-Month2,55689-0093,RR067e_00190_W-

R310_A1_S94,100396386,3875134,3.86,452240,0.45,86.484,8.82,18230526,17
910542,15766450,1788416,Human,76,14209,206.806,72.3515,Nasopharyngeal
Swab,18360789,36.58,2.00E+07,94.31,775455,4.22,265429,1.45,no,no,no,no
,pass,0
121,55689-0093-Month3,55689-0093,RR067e_00201_W-
R310_A9_S157,345364,7756,2.246,3642,1.05,34.951,1.78,149302,86722,5218
2,29338,Human,79,6769,215.294,76.5644,Nasopharyngeal
Swab,52350,30.32,47523,90.78,2356,4.5,2471,4.72,no,no,no,no,pass,0
122,55689-0093-V8,55689-0093,RR067e_00724_W-
R311_B7_S236,999660,418,0.042,5324,0.53,4.22,2.51,952494,112812,40194,
16040,Human,87,463,202.219,70.4797,Nasopharyngeal
Swab,15601,3.12,11235,72.01,183,1.17,4180,26.79,no,no,no,yes,fail,0
123,55689-0094-Month3,55689-0094,RR067e_00205_W-
R311_D8_S246,60850,54,0.089,23052,37.88,47.409,4.42,13584,7056,6440,14
56,Human,84,1371,210.814,71.9115,Nasopharyngeal
Swab,22829,75.03,9186,40.24,204,0.89,13424,58.8,no,yes,no,no,fail,0
124,55689-0095-Month2,55689-0095,RR067e_00186_W-
R309_E2_S13,2836264,2664,0.094,4156,0.15,68.095,1.95,470940,446846,320
688,164862,Human,81,7128,215.839,77.3179,Nasopharyngeal
Swab,432676,30.51,420828,97.26,9343.69,2.16,2444,0.56,no,no,no,no,pass
,0
125,55689-0099 V5,55689-0099,RR067e_00616_W-
R310_B7_S143,502490,22990,4.575,2714,0.54,57.857,2.32,122056,88948,706
18,30450,Human,80,6871,215.627,76.431,Nasopharyngeal
Swab,92368,36.76,84665.2,91.66,6045.84,6.55,1572,1.7,no,no,no,no,pass,
0
126,55689-0101 V5,55689-0101,RR067e_00618_W-
R309_C9_S66,4340632,57640,1.328,25184,0.58,53.383,4.71,1284222,821486,
685562,145662,Human,79,7477,197.46,67.044,Nasopharyngeal
Swab,937607,43.2,864824,92.24,57741.1,6.16,14557,1.55,no,no,no,no,pass
,0
127,55689-0103 V6,55689-0103,RR067e_00596_W-
R310_A6_S134,281904,16024,5.684,3738,1.33,17.837,1.91,206842,48894,368
94,19278,Human,84,2360,210.327,72.8297,Nasopharyngeal
Swab,22897,16.24,19238,84.02,1327,5.8,2332,10.18,no,no,no,no,pass,0
128,55689-0105 V6,55689-0105,RR067e_00649_W-
R310_H11_S180,190752,118,0.062,2764,1.45,36.482,1.66,45140,22960,16468
,9912,Human,81,7000,225.377,80.1968,Nasopharyngeal
Swab,47240,49.53,44112,93.38,1526,3.23,1598,3.38,no,no,no,no,pass,0
129,55689-0114 V5,55689-0114,RR067e_00642_W-
R311_F9_S256,3002728,125016,4.163,45662,1.52,7.099,5.62,2678200,221272
,190114,33824,Human,82,2575,206.923,70.4952,Nasopharyngeal
Swab,138106,9.2,104243,75.48,6446,4.67,27252,19.73,no,no,no,no,pass,0
130,55689-0115--Month2,55689-0115,RR067e_00202_W-
R309_B1_S2,1768352,4834,0.273,724,0.04,92.095,1.48,265008,263494,24406
0,165064,Human,81,11563,197.829,64.0582,Nasopharyngeal
Swab,372023,42.08,355570,95.58,15909.5,4.28,422,0.11,no,no,no,no,pass,
0
131,55689-0118 V4,55689-0118,RR067e_00615_W-
R310_E2_S106,323634,7920,2.447,958,0.3,61.014,1.85,94244,90734,57502,3

1074,Human,82,6345,219.965,78.0485,Nasopharyngeal
Swab,59976,37.06,55678,92.83,3696,6.16,592,0.99,no,no,no,no,pass,0
132,55689-0120 V4,55689-0120,RR067e_00622_W-
R311_G8_S249,5137896,3546932,69.035,44142,0.86,81.372,8,4973708,480536
2,4047218,505674,Human,82,768,216.281,73.1198,Nasopharyngeal
Swab,180241,7.02,22485,12.47,127008,70.47,30748,17.06,no,no,yes,no,fai
l,1
133,55689-0132-D0,55689-0132,RR067e_00197_W-
R310_A8_S149,593986,64010,10.776,8764,1.48,19.16,2.71,462208,103288,88
558,32648,Human,86,2795,214.893,76.9427,Nasopharyngeal
Swab,30629,10.31,23144.1,75.56,2395.93,7.82,5087,16.61,no,no,no,no,pas
s,0
134,55689-0138 V3,55689-0138,RR067e_00638_W-
R311_C1_S189,855994,43192,5.046,17936,2.1,46.248,3.77,564824,468858,26
1222,69256,Human,79,711,200.057,64.7148,Nasopharyngeal
Swab,128031,29.91,98609.1,77.02,16798.9,13.12,12553,9.8,no,no,no,no,pa
ss,0
135,55689-0141 V3,55689-0141,RR067e_00643_W-
R310_F10_S170,428870,26358,6.146,8818,2.06,71.459,2.34,92640,77088,662
00,28332,Human,81,6240,224.488,80.9716,Nasopharyngeal
Swab,103074,48.07,94944,92.11,3000,2.91,5107,4.95,no,no,no,no,pass,0
136,55689-0150 V1,55689-0150,RR067e_00593_W-
R309_B8_S58,1849152,23420,1.267,5000,0.27,72.259,1.98,297512,241736,21
4980,108310,Human,80,6897,211.523,72.968,Nasopharyngeal
Swab,365596,39.54,349492,95.6,13193.8,3.61,2866,0.78,no,no,no,no,pass,
1
137,55689-0151 V1,55689-0151,RR067e_00594_W-
R310_B1_S95,259490,28008,10.793,1580,0.61,57.125,2.18,132482,127144,75
680,34720,Human,83,1479,210.315,70.5731,Nasopharyngeal
Swab,42223,32.54,38801.1,91.9,2353.94,5.58,1049,2.48,no,no,no,no,pass,
1
138,55689-0161 V2,55689-0161,RR067e_00629_W-
R311_H7_S242,395354,150272,38.009,15000,3.79,83.329,6.09,211272,186094
,176050,28900,Human,82,1168,230.545,81.7727,Nasopharyngeal
Swab,72566,36.71,59971,82.64,4289,5.91,8306,11.45,no,no,no,no,pass,0
139,55689-0172-D7,55689-0172,RR067e_00710_W-
R311_C11_S269,2372658,1254364,52.867,66552,2.8,80.547,8.71,1848370,157
9956,1488812,170892,Human,83,4594,213.354,73.1531,Nasopharyngeal
Swab,250086,21.08,129575,51.81,80645,32.25,39624,15.84,no,no,no,no,pas
s,0
140,55689-0177-Month2,55689-0177,RR067e_00706_W-
R309_D10_S75,3806540,8934,0.235,9248,0.24,54.631,5.25,1280746,865670,6
99682,133350,Human,81,8530,182.367,57.0066,Nasopharyngeal
Swab,851763,44.75,827170,97.11,18584.7,2.18,5702,0.67,no,no,no,no,pass
,0
141,55689-0180 V1,55689-0180,RR067e_00645_W-
R310_D6_S137,585962,2326,0.397,2002,0.34,4.878,2.19,437310,25100,21332
,9728,Human,81,8310,212.874,74.9696,Nasopharyngeal
Swab,48985,16.72,45695.4,93.28,2096.65,4.28,1188,2.43,no,no,no,no,pass
,0

142,55689-0181 V1,55689-0181,RR067e_00647_W-
R310_E7_S145,880254,562,0.064,840,0.1,0.865,2.02,837386,14574,7246,359
6,Human,82,5850,222.14,76.5567,Nasopharyngeal
Swab,12359,2.81,11318,91.58,568,4.6,469,3.79,no,no,no,no,pass,0
143,55689-0181-D30,55689-0181,RR067e_00685_W-
R309_E6_S45,3257214,2070,0.064,10548,0.32,58.429,3.08,840654,719770,49
1182,159578,Human,81,9319,206.13,70.8685,Nasopharyngeal
Swab,651349,39.99,605565,92.97,39235,6.02,6313,0.97,no,no,no,no,pass,0
144,55689-0181-Month2,55689-0181,RR067e_00691_W-
R310_A2_S102,904058,1160,0.128,1578,0.17,7.766,2.1,603144,54244,46842,
22358,Human,81,6812,198.308,65.6954,Nasopharyngeal
Swab,80346,17.77,77337.1,96.26,2114.87,2.63,891,1.11,no,no,no,no,pass,
0
145,55689-0182 V1,55689-0182,RR067e_00652_W-
R310_H2_S109,342140,2562,0.749,1634,0.48,35.588,1.79,110498,51048,3932
4,21926,Human,82,6836,210.365,72.1402,Nasopharyngeal
Swab,55860,32.65,52143.3,93.35,2734.7,4.9,967,1.73,no,no,no,no,pass,0
146,55689-0183 V1,55689-0183,RR067e_00651_W-
R310_D3_S113,237996,3310,1.391,2206,0.93,47.742,1.93,74580,57616,35606
,18460,Human,84,7027,216.767,75.8795,Nasopharyngeal
Swab,44349,37.27,41286,93.09,1648,3.72,1384,3.12,no,no,no,no,pass,0
147,55689-0184 V1,55689-0184,RR067e_00644_W-
R311_H8_S250,1895848,159620,8.419,100482,5.3,18.422,7.54,1302074,28035
2,239872,31810,Human,85,1840,226.142,80.5644,Nasopharyngeal
Swab,208805,22.03,142094,68.05,9599,4.6,56996,27.3,no,no,no,no,pass,0
148,55689-0185 V1,55689-0185,RR067e_00646_W-
R310_F2_S107,11089568,29738,0.268,66110,0.6,45.564,4.87,2918952,182493
6,1329982,273052,Human,74,10642,213.839,75.2433,Nasopharyngeal
Swab,2867866,51.72,3.00E+06,93.21,155148,5.41,38401,1.34,no,no,no,no,p
ass,0
149,55689-0185-Month3,55689-0185,RR067e_00678_W-
R309_E4_S29,4054606,1160,0.029,6114,0.15,84.933,1.78,384458,369422,326
532,183502,Human,80,7946,216.762,71.446,Nasopharyngeal
Swab,1027082,50.66,996717,97.04,26908.3,2.62,3397,0.33,no,no,no,no,pas
s,0
150,55689-0187 V1,55689-0187,RR067e_00663_W-
R309_D7_S52,3591106,23046,0.642,70490,1.96,76.008,4.32,700246,660302,5
32240,123316,Human,78,7384,207.269,70.7875,Nasopharyngeal
Swab,974093,54.25,848386,87.09,82545.8,8.47,42278,4.34,no,no,no,no,pas
s,0
151,55689-0190 V1,55689-0190,RR067e_00666_W-
R310_H7_S148,489606,4462,0.911,7162,1.46,63.254,2.11,93566,71866,59184
,28062,Human,80,6748,215.255,75.8544,Nasopharyngeal
Swab,112270,45.86,101702,90.59,6412.99,5.71,4096,3.65,no,no,no,no,pass
,0
152,55689-0191 V1,55689-0191,RR067e_00664_W-
R310_H3_S117,1599244,3058,0.191,7536,0.47,81.072,2.22,187860,180674,15
2302,68746,Human,78,8684,218.008,75.9626,Nasopharyngeal
Swab,362730,45.36,341963,94.27,16407.1,4.52,4304,1.19,no,no,no,no,pass
,0

153,55689-0192-D0,55689-0192,RR067e_00737_W-
R309_E9_S68,2522210,28010,1.111,10152,0.4,85.774,2.25,236658,233282,20
2990,90388,Human,78,8024,230.976,81.7111,Nasopharyngeal
Swab,539967,42.82,510415,94.53,23967.9,4.44,5522,1.02,no,no,no,no,pass
,0
154,55689-0196-D0,55689-0196,RR067e_00674_W-
R311_D1_S190,823218,313614,38.096,13826,1.68,83.615,4.1,461512,435576,
385894,94186,Human,84,6553,217.753,79.8562,Nasopharyngeal
Swab,96778,23.51,82012.1,84.74,6830.93,7.06,7866,8.13,no,no,no,no,pass
,0
155,55689-0200-D0,55689-0200,RR067e_00677_W-
R310_F11_S178,1333704,15618,1.171,5720,0.43,6.628,2.2,951528,98574,630
72,28662,Human,81,6922,222.884,77.9099,Nasopharyngeal
Swab,127646,19.14,117444,92.01,6817.79,5.34,3311,2.59,no,no,no,no,pass
,0
156,55689-0201-D0,55689-0201,RR067e_00732_W-
R311_B9_S252,153504,3428,2.233,2610,1.7,15.855,1.92,94366,21284,14962,
7808,Human,84,1273,207.84,71.9341,Nasopharyngeal
Swab,17453,22.74,14624.2,83.79,1197.85,6.86,1601,9.17,no,no,no,no,pass
,0
157,55689-0203-D0,55689-0203,RR067e_00698_W-
R309_D5_S36,121114,1506,1.243,692,0.57,42.917,1.61,60204,52332,25838,1
6046,Human,84,1268,212.818,72.0712,Nasopharyngeal
Swab,19172,31.66,17798,92.83,955.96,4.99,416,2.17,no,no,no,no,pass,0
158,55689-0205-D0,55689-0205,RR067e_00708_W-
R309_C2_S11,6026622,4024,0.067,3502,0.06,68.731,2.5,1362318,1334804,93
6328,374236,Human,79,8034,206.934,71.1404,Nasopharyngeal
Swab,1366026,45.33,1.00E+06,95.7,56289.2,4.12,2288,0.17,no,no,no,no,pa
ss,0
159,55689-0207-D0,55689-0207,RR067e_00688_W-
R310_B2_S103,10359668,340506,3.287,44532,0.43,40.799,5.15,3790048,2040
648,1546288,300458,Human,80,13926,203.584,67.721,Nasopharyngeal
Swab,1823422,35.2,2.00E+06,92.25,114191,6.26,26396,1.45,no,no,no,no,pa
ss,0
160,55689-0208-D0,55689-0208,RR067e_00676_W-
R310_C10_S167,1267796,15654,1.235,6818,0.54,8.806,1.89,1052164,205942,
92656,48958,Human,82,4367,208.095,70.5199,Nasopharyngeal
Swab,70745,11.16,62616.1,88.51,3821.93,5.4,4250,6.01,no,no,no,no,pass,
1
161,55689-0210-D0,55689-0210,RR067e_00689_W-
R309_C11_S82,2187590,6552,0.3,5520,0.25,77.431,1.91,309436,302150,2396
00,125328,Human,81,9035,218.459,76.7264,Nasopharyngeal
Swab,553907,50.64,525260,94.83,25468.2,4.6,3114,0.56,no,no,no,no,pass,
0
162,55689-0211-D0,55689-0211,RR067e_00718_W-
R309_C7_S51,2228410,2402,0.108,4166,0.19,55.363,1.95,724808,696946,401
274,206294,Human,77,8591,211.491,72.8652,Nasopharyngeal
Swab,525154,47.13,500513,95.31,21716,4.14,2807,0.53,no,no,no,no,pass,0
163,55689-0212-D0,55689-0212,RR067e_00684_W-
R310_D12_S184,2196608,472,0.021,12396,0.56,46.808,2.01,680458,519570,3

18510,158590,Human,78,7338,213.433,75.1656,Nasopharyngeal
Swab,464442,42.29,418274,90.06,38198.3,8.22,7733,1.67,no,no,no,no,pass
,0
164,55689-0213-D0,55689-0213,RR067e_00683_W-
R309_B12_S89,3220216,90402,2.807,62880,1.95,50.049,4.18,1207754,102463
0,604470,144642,Human,76,5400,216.995,79.141,Nasopharyngeal
Swab,695252,43.18,594439,85.5,60556.6,8.71,40010,5.75,no,no,no,no,pass
,0
165,55689-0215-D0,55689-0215,RR067e_00727_W-
R310_F6_S139,156688,3752,2.395,2294,1.46,28.591,2.01,58004,23264,16584
,8234,Human,85,9676,227.768,80.6116,Nasopharyngeal
Swab,28631,36.55,26493,92.53,844,2.95,1291,4.51,no,no,no,no,pass,0
166,55689-0217-D0,55689-0217,RR067e_00693_W-
R309_H2_S16,2969004,1381816,46.541,94350,3.18,89.937,3.52,1741362,1695
302,1566124,444806,Human,81,6999,229.368,85.1112,Nasopharyngeal
Swab,331535,22.33,238600,71.97,39545.9,11.93,53207,16.05,no,no,no,no,p
ass,0
167,55689-0218-D0,55689-0218,RR067e_00726_W-
R311_D3_S206,3096736,1813264,58.554,77816,2.51,77.113,8.57,2790744,256
3508,2152020,251048,Human,87,802,212.867,73.1606,Nasopharyngeal
Swab,128215,8.28,61729.8,48.15,18000.2,14.04,48299,37.67,no,yes,no,no,
fail,0
168,55689-0220-D0,55689-0220,RR067e_00703_W-
R310_H1_S101,17601428,199486,1.133,76610,0.44,55.012,7.27,4024760,2438
956,2214098,304390,Human,75,13298,205.235,69.2162,Nasopharyngeal
Swab,4070003,46.25,4.00E+06,93.18,232268,5.71,44619,1.1,no,no,no,no,pa
ss,0
169,55689-0221-D0,55689-0221,RR067e_00712_W-
R309_H5_S40,6885664,24152,0.351,11856,0.17,2.223,3.19,6417766,231648,1
42674,44672,Human,83,1481,201.128,68.9598,Nasopharyngeal
Swab,134184,3.9,125101,93.23,1536.45,1.15,7541,5.62,yes,no,no,no,fail,
0
170,55689-0222-D0,55689-0222,RR067e_00717_W-
R309_C3_S19,4032592,5478,0.136,18324,0.45,64.208,4.46,1099378,1006712,
705892,158392,Human,78,7393,198.578,66.7598,Nasopharyngeal
Swab,769523,38.17,743511,96.62,14749,1.92,11051,1.44,no,no,no,no,pass,
0
171,55689-0222-Month2,55689-0222,RR067e_00679_W-
R311_D12_S277,2942450,11320,0.385,9974,0.34,86.886,2.74,447856,434398,
389122,142274,Human,79,9651,201.338,66.8145,Nasopharyngeal
Swab,652086,44.32,621339,95.28,24794.2,3.8,5810,0.89,no,no,no,no,pass,
0
172,55689-0223-D0,55689-0223,RR067e_00714_W-
R309_B5_S34,4119722,2512,0.061,7708,0.19,32.566,4.37,1400958,489950,45
6234,104404,Human,81,8127,191.188,61.6046,Nasopharyngeal
Swab,778948,37.82,762327,97.87,12067.5,1.55,4505,0.58,no,no,no,no,pass
,0
173,55689-0224-D0,55689-0224,RR067e_00725_W-
R309_E1_S5,3654536,12938,0.354,6306,0.17,53.733,4.65,778432,496714,418
272,89962,Human,78,6499,205.955,71.5816,Nasopharyngeal

Swab,611567,33.47,598636,97.89,9116.86,1.49,3794,0.62,no,no,no,no,pass
,0
174,55689-0225-D0,55689-0225,RR067e_00681_W-
R309_D11_S83,6211458,22910,0.369,4120,0.07,1.297,3.48,6037404,108348,7
8316,22484,Human,85,4469,187.822,58.7617,Nasopharyngeal
Swab,53925,1.74,50072.1,92.86,1165.85,2.16,2683,4.98,yes,no,no,no,fail
,0
175,55689-0226-D0,55689-0226,RR067e_00713_W-
R309_H9_S71,230662,300,0.13,1190,0.52,6.01,1.61,191684,20990,11520,715
0,Human,81,2825,199.968,64.499,Nasopharyngeal
Swab,10236,8.88,9244.55,90.31,225.454,2.2,766,7.48,yes,no,no,no,fail,0
176,55689-0231-D0,55689-0231,RR067e_00690_W-
R311_H5_S226,178404,32942,18.465,33714,18.9,64.617,5.28,75652,56310,48
884,9254,Human,85,2523,210.371,71.8516,Nasopharyngeal
Swab,39650,44.45,18422,46.46,1858.99,4.69,19369,48.85,no,yes,no,no,fai
l,0
177,55689-0232-D0,55689-0232,RR067e_00667_W-
R311_E9_S255,796280,7670,0.963,52374,6.58,29.044,3.38,497266,292316,14
4424,42702,Human,85,869,194.899,62.0031,Nasopharyngeal
Swab,106484,26.75,69924.1,65.67,2342.93,2.2,34179,32.1,no,yes,no,no,fa
il,0
178,55689-0235-D0,55689-0235,RR067e_00701_W-
R311_A4_S211,139526,3318,2.378,2980,2.14,5.435,1.98,116054,6818,6308,3
190,Human,87,749,211.6,75.0482,Nasopharyngeal
Swab,6562,9.41,4604,70.16,251,3.83,1697,25.86,no,no,no,no,pass,0
179,55689-0247-D0,55689-0247,RR067e_00707_W-
R310_E3_S114,12492776,48946,0.392,71262,0.57,44.813,4.58,5329296,44381
80,2388198,521660,Human,78,7363,204.692,69.2652,Nasopharyngeal
Swab,2260582,36.19,2.00E+06,94.44,78121.6,3.46,47154,2.09,no,no,no,no,
pass,0
180,OHC-001-V03,OHC-001,RR067e_00095_W-
R310_C9_S159,415124,2864,0.69,2406,0.58,3.962,2.07,364192,23548,14430,
6982,Human,83,5394,189.667,58.3025,Nasopharyngeal
Swab,17535,8.45,15220,86.8,852,4.86,1462,8.34,no,no,no,no,pass,0
181,OHC-001-V04,OHC-001,RR067e_00164_W-
R311_C6_S229,461592,413158,89.507,6616,1.43,92.141,3.62,452644,428180,
417072,115090,Human,80,540,204.779,68.0129,Nasopharyngeal
Swab,4769,2.07,897,18.81,18,0.38,3846,80.65,no,yes,no,no,fail,0
182,OHC-007-V03,OHC-007,RR067e_00099_W-
R309_B9_S65,4526298,69316,1.531,79202,1.75,5.056,4.71,3961750,382914,2
00288,42486,Human,82,3116,207.632,70.3267,Nasopharyngeal
Swab,192496,8.51,143069,74.32,3199.99,1.66,46199,24,no,no,no,no,pass,0
183,OHC-008-V03,OHC-008,RR067e_00101_W-
R311_E11_S271,758938,145046,19.112,38768,5.11,29.464,5.93,623556,24591
4,183726,30992,Human,87,515,215.875,74.9859,Nasopharyngeal
Swab,33245,8.76,10243,30.81,296.963,0.89,22705,68.3,no,yes,no,no,fail,
0
184,OHC-014-V03,OHC-014,RR067e_00158_W-
R311_H2_S202,527456,39160,7.424,15010,2.85,35.236,3.01,445242,359364,1
56884,52172,Human,89,589,204.775,73.8681,Nasopharyngeal

Swab,25154,9.54,12317,48.97,453,1.8,12375,49.2,no,yes,no,no,fail,0
185,OHC-030-V01,OHC-030,RR067e_00103_W-
R311_B4_S212,597846,6330,1.059,104892,17.54,12.344,4.17,351404,52404,4
3378,10414,Human,80,1709,215.035,73.4668,Nasopharyngeal
Swab,94733,31.69,34506,36.42,674,0.71,59315,62.61,no,yes,no,no,fail,0
186,OHC-OHC001-V02,OHC-001,RR067e_00031_W-
R311_E4_S215,127122,558,0.439,3732,2.94,60.305,1.8,28064,23198,16924,9
406,Human,85,8146,208.511,69.169,Nasopharyngeal
Swab,25016,39.36,21107,84.37,1648,6.59,2259,9.03,no,no,no,no,pass,0
187,OHC-OHC003-V02,OHC-003,RR067e_00013_W-
R311_A12_S275,82346,42,0.051,404,0.49,1.861,1.33,81262,3022,1512,1140,
Human,100,435,206.279,78.0945,Nasopharyngeal
Swab,2125,5.16,1787,84.09,13,0.61,325,15.29,yes,no,no,no,fail,0
188,OHC-OHC007-V02,OHC-007,RR067e_00032_W-
R311_G4_S217,191390,3060,1.599,23232,12.14,13.73,2.43,137702,32132,189
06,7772,Human,84,602,214.744,69.9897,Nasopharyngeal
Swab,19469,20.34,5568,28.6,580,2.98,13310,68.37,no,yes,no,no,fail,0
189,OHC001-V05,OHC-001,RR067e_00759_W-
R310_B3_S111,26325872,39290,0.149,64966,0.25,53.336,3.97,6896130,60221
28,3678114,926308,Human,74,9747,213.116,74.3998,Nasopharyngeal
Swab,6459281,49.07,6.00E+06,95.81,227014,3.51,42118,0.65,no,no,no,no,p
ass,0
190,OHC003-V06,OHC-003,RR067e_00749_W-
R310_H5_S133,755334,152850,20.236,7300,0.97,76.568,2.86,307190,271282,
235210,82358,Human,80,5866,207.822,72.869,Nasopharyngeal
Swab,142014,37.6,121813,85.78,15786,11.12,4343,3.06,no,no,no,no,pass,0
191,OHC003-V08,OHC-003,RR067e_00765_W-
R311_E3_S207,1145092,59540,5.2,25996,2.27,60.299,4.35,292976,233144,17
6662,40606,Human,79,2439,221.837,80.6362,Nasopharyngeal
Swab,270909,47.32,245177,90.5,10376,3.83,15314,5.65,no,no,no,no,pass,0
192,OHC004-V02,OHC-004,RR067e_00758_W-
R309_G6_S47,3745084,189756,5.067,10890,0.29,18.029,4.38,2064108,458460
,372134,85042,Human,80,7359,227.919,81.0213,Nasopharyngeal
Swab,471013,25.15,450705,95.69,13880.8,2.95,6322,1.34,no,no,no,no,pass
,0
193,OHC007-V06,OHC-007,RR067e_00762_W-
R309_B10_S73,3438828,148578,4.321,8946,0.26,38.159,2.39,1504576,868776
,574124,239732,Human,78,8022,212.36,74.3625,Nasopharyngeal
Swab,677512,39.4,636556,93.95,34736.4,5.13,5692,0.84,no,no,no,no,pass,
0
194,OHC018-V05,OHC-018,RR067e_00217_W-
R310_G1_S100,7342500,122280,1.665,49772,0.68,17.061,4.83,3252318,68657
2,554872,114866,Human,77,8157,213.98,72.3091,Nasopharyngeal
Swab,1246887,33.96,1.00E+06,91.92,72407.9,5.81,27740,2.22,no,no,no,no,
pass,0
195,OHC018-V09,OHC-018,RR067e_00768_W-
R310_H6_S141,134352,3568,2.656,4202,3.13,47.866,1.72,51632,42750,24714
,14376,Human,81,6822,222.624,81.5625,Nasopharyngeal
Swab,34762,51.75,28835.2,82.95,3301.8,9.5,2607,7.5,no,no,no,no,pass,0
196,OHC019-V06,OHC-019,RR067e_00216_W-

R309_H11_S87,4449546,50430,1.133,23222,0.52,21.408,2.88,2529256,994578,541472,187700,Human,83,6631,217.987,80.2459,Nasopharyngeal Swab,576925,25.93,541411,93.84,20356.8,3.53,14996,2.6,no,no,no,no,pass,0
197,OHC019-V09,OHC-019,RR067e_00769_W-R310_E5_S130,472434,4786,1.013,4516,0.96,31.258,1.67,232182,138590,72576,43478,Human,83,2963,217.113,76.7183,Nasopharyngeal Swab,76647,32.45,71419.3,93.18,2199.67,2.87,3023,3.94,no,no,no,no,pass,0
198,OHC028-V0,OHC-028,RR067e_00756_W-R311_F8_S248,10130490,23298,0.23,8324,0.08,0.525,7.22,10042554,163196,52710,7302,Human,82,5770,207.086,67.6234,Nasopharyngeal Swab,37986,0.75,31637,83.29,1373,3.61,4973,13.09,yes,no,no,no,fail,0
199,OHC030-V06,OHC-030,RR067e_00755_W-R310_B8_S150,365142,117152,32.084,2896,0.79,50.463,3.99,280322,158718,141458,35414,Human,83,1861,217.75,80.5048,Nasopharyngeal Swab,26167,14.33,21586,82.49,2734,10.45,1840,7.03,no,no,no,no,pass,0
200,55689-0040-Month4,55689-0040,RR067e_00207_W-R309_A2_S9,3374624,26378,0.782,3330,0.1,65.734,2.61,609376,434452,400568,153330,Human,77,10318,206.957,71.5088,Nasopharyngeal Swab,639264,37.89,604159,94.51,33171.2,5.19,1822,0.29,no,no,no,no,pass,0
201,55689-0062-Month4,55689-0062,RR067e_00208_W-R309_D1_S4,3669232,228442,6.226,5076,0.14,32.326,6.72,1383384,479038,447186,66592,Human,83,6849,224.494,81.6985,Nasopharyngeal Swab,459540,25.05,444125,96.65,12404,2.7,2925,0.64,no,no,no,no,pass,1
202,55689-0043-Month4,55689-0043,RR067e_00209_W-R310_B6_S135,564302,158798,28.141,8432,1.49,81.174,2.77,262344,248716,212956,76888,Human,78,4357,217.779,78.5772,Nasopharyngeal Swab,69023,24.46,60854.1,88.16,3222.93,4.67,4900,7.1,no,no,no,no,pass,0
203,55689-0047-Month4,55689-0047,RR067e_00210_W-R310_F4_S123,499672,21160,4.235,6820,1.36,44.506,2.02,139176,73634,61942,30740,Human,80,6842,234.273,86.7349,Nasopharyngeal Swab,80761,32.33,73508,91.02,3509,4.34,3731,4.62,no,no,no,no,pass,0
